# Targeting LGSN restores sensitivity to chemotherapy in gastric cancer stem cells by triggering pyroptosis

**DOI:** 10.1038/s41419-023-06081-8

**Published:** 2023-08-23

**Authors:** Yu-Ting Li, Xiang-Yu Tan, Li-Xiang Ma, Hua-Hui Li, Shu-Hong Zhang, Chui-Mian Zeng, Liu-Na Huang, Ji-Xian Xiong, Li Fu

**Affiliations:** 1 Guangdong Province Key Laboratory of Regional Immunity and Diseases, Department of Pharmacology and International Cancer Center, Shenzhen University Medical School, Shenzhen University, Shenzhen, Guangdong 518055 China; 2 Shenzhen University-Friedrich Schiller Universität Jena Joint PhD Program in Biomedical Sciences, Shenzhen University Medical School, Shenzhen, Guangdong 518055 China; 3 Department of Endocrinology and Diabetes Center, The First Affiliated Hospital of Sun Yat-Sen University, Guangzhou, China

**Keywords:** Cancer stem cells, Gastric cancer

## Abstract

Gastric cancer (GC) is notoriously resistant to current therapies due to tumor heterogeneity. Cancer stem cells (CSCs) possess infinite self-renewal potential and contribute to the inherent heterogeneity of GC. Despite its crucial role in chemoresistance, the mechanism of stemness maintenance of gastric cancer stem cells (GCSCs) remains largely unknown. Here, we present evidence that lengsin, lens protein with glutamine synthetase domain (LGSN), a vital cell fate determinant, is overexpressed in GCSCs and is highly correlated with malignant progression and poor survival in GC patients. Ectopic overexpression of LGSN in GCSC-derived differentiated cells facilitated their dedifferentiation and treatment resistance by interacting with vimentin and inducing an epithelial-to-mesenchymal transition. Notably, genetic interference of LGSN effectively suppressed tumor formation by inhibiting GCSC stemness maintenance and provoking gasdermin-D-mediated pyroptosis through vimentin degradation/NLRP3 signaling. Depletion of LGSN combined with the chemo-drugs 5-fluorouracil and oxaliplatin could offer a unique and promising approach to synergistically rendering this deadly cancer eradicable in vivo. Our data place focus on the role of LGSN in GCSC regeneration and emphasize the critical importance of pyroptosis in battling GCSC.

## Introduction

Gastric cancer (GC) is a malignancy with high incidence and mortality rates in the world [1]. Gastric carcinogenesis is a multi-step process [2] with epithelial cell origins [3]. Recently, growing attention has been given to the discovery that premalignant mutations of adult stem cells residing in the gastric epithelium might be a direct cause of cancer, given that these cells have multi-directional differentiation potential [2, 4]. Normal adult stem cells can be transformed into cancer stem cells (CSCs) and subsequently divide into tumor cells through oncogenic signal stimuli [4]. A population of pluripotent CSCs continues to exist in neoplastic tissue and maintains GC heterogeneity, which compromises the efficacy of chemotherapy by promoting the frequent emergence of intrinsic and acquired chemo-drug resistance. Although most cancer chemoresistance, metastasis, and relapse are attributed to the continuous regeneration of CSCs, little is known about the critical properties and exact mechanisms of CSC death evasion and potential intervenable targets [5].

Cell death signaling is not random and is dictated by intrinsic cell factors [6]. In normal physiological conditions, multiple components of the signal transduction cascade that regulate or participate in regulated cell death (RCD) are involved in the terminal differentiation of a variety of cell types [7], such as keratinized epithelial [8] and lens cells [9]. On unlocking the normally restricted capability of cells for phenotypic plasticity and their escape from a state of terminal differentiation, these signal components initiate cancer pathogenesis [10]. RCD serves as a natural barrier to cancer and has been implicated in mediating the pathology of CSCs [6]. Yet much remains unknown about the interplay between RCD signals of GCSCs and their dedifferentiation process or chemoresistance.

By comparing the gene-expression profiles between undifferentiated patient-derived CD44^+^/CD54^+^ GCSC spheroids and the corresponding GCSC-derived differentiated monolayer cells, we identified LGSN, the product of a nonfunctional pseudoexon and a member of the glutamine synthetase I superfamily [11], could help provide insights into this question. LGSN is a key intermediate filament (IF) terminal-differentiation-associated regulator in the lens [12]. Programmed cell death is a vital procedure in the lens fiber differentiation [9], and major efforts to understand LGSN have so far focused on lens [12] and cataract diseases [13]. However, other studies into cancer have identified LGSN as a tumor-associated antigen and revealed its essential role in lung carcinoma cell survival [11]. Recently, single-cell RNA-sequencing (scRNA-seq) analysis showed that LGSN is highly expressed in fetal stomach multipotent progenitor cells [14]. Yet, these studies did not consider the possible role of LGSN in CSCs, despite its correlation with poor prognosis and lens disease progression. Intriguingly, our RNA-seq efforts in the clinical fresh-isolated GCSCs have found that LGSN is more highly expressed in GCSC spheroids than in adherent cells. This implies there is an association between LGSN and GC initiation and stemness potential. However, it is unclear how the increased expression of LGSN in patients promotes the onset of GC, chemoresistance, and related defenses against cell death. In this study, we aimed to explore the contribution of LGSN to stemness and tumorigenesis and its role in modulating GCSC responses to chemotherapy during GC progression.

## Materials and methods

Additional detailed descriptions of the reagents and antibodies, in vitro function assays, molecular or cellular biochemistry assays, and statistical studies are provided in *Supplementary information*.

### Cell lines

GC patients-derived CD44 + /CD54+ gastric CSCs (GCSC1 and GCSC2) were kind gifts from Dr. Xianming Mo (Sichuan University) and were cultured in vitro according to a previous description [15]. The human normal gastric epithelial cell line GES-1 and GC cell lines (AGS, HGC-27 and MGC-803) were obtained from Cobioer Biosciences. 293 T cell line was obtained from the American Type Culture Collection. GES-1 and 293 T cells were cultured in DMEM (Gibco) while GC cell lines were cultured in RPMI-1640 (Gibco), respectively. All DMEM/RPMI-1640 media contained 10% fetal bovine serum (FBS; Gibco) and antibiotics (100 U/mL penicillin and 100 μg/ml streptomycin; Gibco) and incubated at 37 °C in a 5% CO_2_ incubator. All cell lines were recently authenticated by STR and mycoplasma- and chlamydia-free.

### RNA-seq analysis

Total RNA from spheroid GCSCs and GCSC-derived monolayer cells treated with 10% FBS were extracted and purified using TRIzol reagent (Invitrogen) according to the manufacturer’s instructions. RNA quality control was performed using a Bioanalyzer 2100 (Agilent Technologies). For high-throughput sequencing, the construction of stranded RNA-seq libraries was carried out on an Illumina Novaseq 6000. Then, quality control and adapter trimming of base sequencing was performed using FASTQC (version 0.11.2; http://www.bioinformatics.babraham.ac.uk/ projects/fastqc/). The RNA-seq reads were mapped to the GRCh37 reference genome by STAR (version 2.4.2a). The unique mapped reads summarized for each gene were processed in RSEM (version 1.2.29). To identify DEGs, the R/Bioconductor package edgeR (version 3.2.4) was used. Statistical significance was calculated using Student’s *t* test.

### Tissue microarray (TMA) and immunohistochemical (IHC) staining

A human gastric cancer TMA (HStmA180Su19) of 92 patients (including 84 pairs of GC tissue samples matched to their adjacent samples) was purchased from Shanghai Outdo Biotech Co. Ltd (China). All human subjects provided informed consent, and Shenzhen University and Shanghai Outdo Biotech. Co. Ltd. Institutional Review Board approval was acquired for this study. The follow-up information included age, gender, tumor grade, number of lymph node metastases, and time to recurrence and death. The TMA was incubated with the primary antibody rabbit polyclonal anti-human LGSN (1:100, Sigma) and detected with the EnVision+ detection system (Dako) according to the manufacturer’s instructions. Images were taken using Aperio ScanScope XT (Leica Microsystems). LGSN immunostaining of microarrays was independently assessed by three pathologists, and the final immunoreactive score was calculated as previously reported [16]. If the total score was larger than the median score, the case was considered as a high LGSN expression.

### Animal studies

All mouse procedures were approved by and performed in accordance with the Animal Ethical and Welfare Committee of Shenzhen University and followed ARRIVE guidelines. Male BALB/C nude mice (aged 4–6 weeks, 20.0 ± 2.0 g) were purchased from Charles River Laboratories and housed under specific pathogen-free conditions. For the combination of chemotherapy drugs and LGSN depletion therapeutic models, sh*LGSN-* or shNTC-transfected GCSCs (2 × 10^5^ cells in 200 µL PBS per mouse) were injected subcutaneously into the right lower backs of mice. Mice were randomized to treatment groups (five mice per group) when tumor volume reached 50 mm^3^. 5-FU (10 mg/kg/mouse; MCE) and L-OHP (5 mg/kg/mouse; MCE) were injected into the tail vein every 3 days for 2 weeks. The negative control group was injected with the same volume of saline solution. For systemic toxicity assessment models, GCSCs (2 × 10^5^ cells in 200 µL PBS per mouse) were inoculated subcutaneously into the right flank of nude mice. Mice were randomly assigned to treatment groups when tumor volumes reached ~50 mm^3^ 22 days post-inoculation. Adeno-associated virus serotype 9 (AAV9) vector that expressed the GFP-tagged LGSN-shRNA (AAV-sh*LGSN*) or the control shRNA (AAV-shNTC) was directly injected via the tail vein (2.5 × 10^11^ vg/mouse). AAV9 vector was produced by ViGene Biosciences (Shandong, China). The diameters of tumors were measured with a vernier caliper and the body weights of mice were recorded three times a week (combination therapeutic models) or every 5 days (toxicity assessment models). Tumor volume (mm^3^) was calculated using the formula: volume = length × width^2^ × 0.5. At the endpoint of this experiment, mouse tumors and normal vital organs (heart, lung, liver, spleen, kidney and eye) were excised and weighed. Paraffin-embedded organ sections were deparaffinized and examined by H&E staining, immunohistochemistry, and TUNEL assay. The green fluorescence was detected under fresh OCT-embedded tissue sections. The investigator was blinded to the genotypes of animals when assessing the outcome and no animals were excluded from the analyses during the study.

### Statistical analysis

The exact sample size (n) and biological replicates for each of the experiments were described in figure legends. Sample sizes applied in this study were estimated empirically. GraphPad Prism Software (version 9.0) was used for statistical analyses. Two-tailed unpaired Student’s *t* tests were performed for the analysis of two groups and one-way or two-way ANOVA with post-hoc test was performed for multiple comparisons. Fisher’s Exact Test was used to comparisons of categorical variables. Kaplan–Meier plots and log-rank tests were used for the survival analysis. The Wilcoxon test was executed for statistical comparison of any two non-normal distribution groups. The Dunn-Kruskal-Wallis test was adjusted for multiple comparisons of non-normal distribution groups. All quantitative data are shown as mean ± the standard error of the mean (SEM) of three independent experiments. *P < 0.05 was considered significant statistically.

## Results

### LGSN is markedly associated with stemness and poor outcomes in gastric cancer patients

**Fig. 1:**
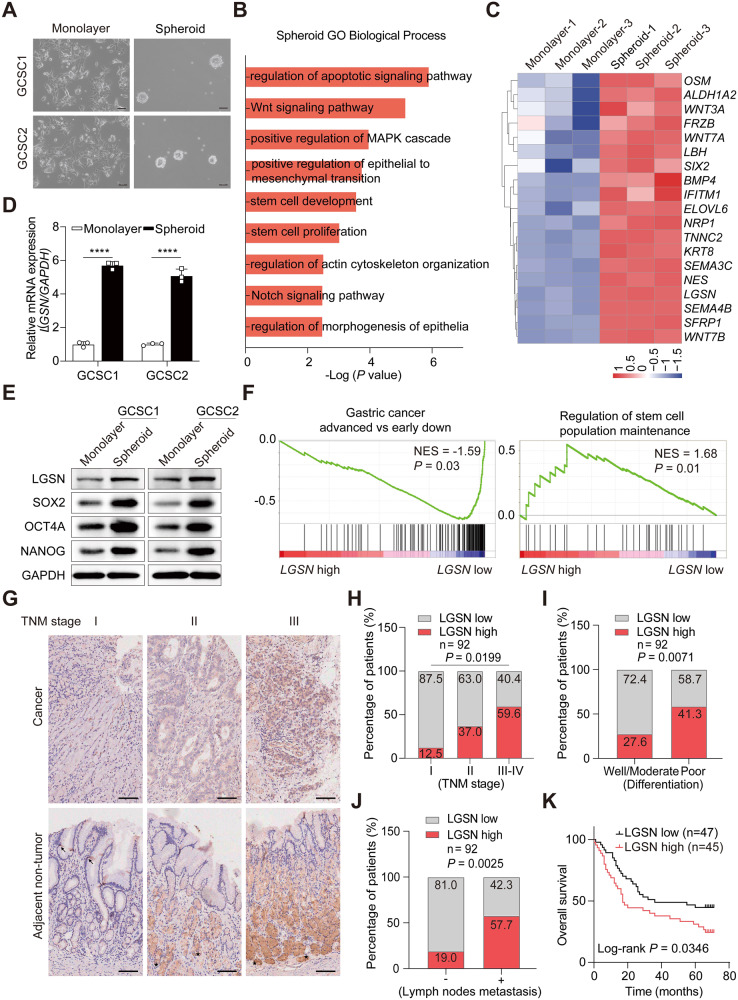
LGSN is predominantly expressed in GCSCs and is correlated with a poor prognosis in GC patients. **A** Representative images of GCSCs in spheroid and differentiated adherent statuses; scale bars, 100 μm. **B** GO enrichment analysis of differentially expressed genes in GCSC spheroids compared with the corresponding differentiated adherent statuses, as determined by RNA-seq analysis. **C** Heatmap representing fold-change in mRNA expression of differentially expressed genes involved in stemness from the RNA-seq profile. **D**, **E** Validation of up-regulation of LGSN mRNA and protein and stemness-related protein expression levels between GCSC spheroids and the corresponding differentiated monolayer GC cells by qRT-PCR (n = 3) (**D**) and western blot (**E**). **F** GSEA plot showing differentially expressed genes in high-*LGSN*-expressing GC from TCGA dataset. **G** IHC staining of LGSN in TMA sections from representative TNM stage tumors. Representative images of tissue samples show low (left), intermediate (middle), and high (right) LGSN expression; scale bars, 100 μm. **H–J** Bar chart summary of LGSN expression significantly associated with advanced tumor stage (**H**), tumor differentiation (**I**), and metastasis (**J**) (Fisher’s Exact Test). The median LGSN expression is used as a cutoff. **K** Kaplan–Meier survival analysis showed that a high level of LGSN staining (*n* = 45) of TMA indicated a worse survival outcome in GC patients. Median LGSN expression was used as the cutoff. NES, normalized enrichment score; FDR, false-discovery rate. ****P < 0.0001; error bars show mean ± SD.

To investigate the key molecules involved in GCSC-specific characteristics, we first examined gene expression by comparing the RNA-seq profiles between patient-derived CD44^+^/CD54^+^ GCSCs growing as undifferentiated spheroids in serum-free GCSC-completed media and growing as differentiated gastric epithelial-like monolayer cells in serum-containing media (Fig. 1A; Supplementary Fig. S1A). Based on Gene Ontology (GO) analysis of our RNA-seq data, 2995 of these differentially expressed genes (DEGs) (Supplementary Table S1) were significantly enriched in pathways related to “stem cell development”, “epithelial-to-mesenchymal transition (EMT)”, and “regulation of apoptotic death”, etc. (Fig. 1B*;* Supplementary Table S2). We then used a list of well-established stemness-regulated gene sets from the Molecular Signatures Database (MSigDB) (https://www.gsea-msigdb.org/gsea/msigdb/index.jsp) to filter the 1111 up-regulated DEGs enriched in GCSC spheroids and identified 18 up-regulated stemness genes (Fig. 1C). Among these genes that are essential for the normal function of vital organs (such as *WNT3A, ELOVL6, WNT7B*, etc), *LGSN* is generally not present in human normal tissues, except for the lens [12]. LGSN is highly expressed in various types of cancers, including lung cancer. Previous study has indicated that targeting LGSN could present a novel therapeutic approach for treating lung cancer [11]. We thus focused on the novel gene *LGSN* in the present study. *LGSN* was up-regulated in spheroids and may govern GCSC stemness, as validated by increased stemness-associated marker expression (Fig. 1C–E; Supplementary Fig. S1B).

To explore the correlation between LGSN expression levels and clinical GC progression, the gene set enrichment analysis (GSEA) analysis using the TCGA dataset was utilized, which further revealed that genes significantly up-regulated along with high-*LGSN* expression were related to “gastric advanced progression” and “regulation of stem cell population maintenance” in GC cells (Fig. 1F). This raised the question of whether the expression and distribution of LGSN correlate with malignant progression in GC patients. We assessed LGSN protein expression on a tissue microarray (TMA) comprising 84 pairs of GC tissues and 8 GC samples without adjacent normal tissues (Supplementary Table S3) and found a stepwise LGSN up-regulation in cancer tissue (Fig. 1G top; Supplementary Fig. S2A). Immunohistochemical (IHC) staining of the TMA also revealed significantly different and relatively high LGSN expression in advanced T stage and TNM stage, poorly differentiated, and lymph node metastasis samples (Fig. 1H–J; Supplementary Fig. S2B; Supplementary Table S4). Based on the GSE29272 dataset, we found that *LGSN* was significantly up-regulated in GC compared with normal gastric tissues (Supplementary Fig. S3A). Importantly, high LGSN expression was significantly correlated with a decreased median survival time in the TMA and Kaplan-Meier Plotter database (Fig. 1K; Supplementary Fig. S3B–D). The above results suggest that LGSN overexpression is an important potential prognostic factor for GC patients.

Of note, the number of LGSN-positive cells from stem-cell residual zones of gland bases increased and their distribution expanded into the isthmus with the development of the TNM stage compared to the paired adjacent non-tumor sections, suggesting that LGSN-positive-cell zone expansion is a unique feature associated with aberrant stem cell proliferation (Fig. 1G, bottom). We further observed that *LGSN* expression was increased in an approximately linear manner, and tumor tissues had higher mRNA/DNA expression-based stemness indexes (mRNAsi/mDNAsi) than adjacent normal tissues from TCGA GC patients (Supplementary Fig. S3E–G). We then found that LGSN overexpression was present in HGC-27 and MGC-823 (Supplementary Fig. S4A). While in spheroid formation conditions, LGSN, EMT-, and stemness-associated protein expression was strikingly increased in all GC and GES-1 cells compared to those cultured in adherent conditions (Supplementary Fig. S4B, C). In addition, we confirmed that protein levels of two dedifferentiation-associated markers, cytokeratin 18 (CK18) and Gastrin, were significantly increased along with the *LGSN* knockdown in GCSCs (Supplementary Fig. S4D). Taken together, these findings indicated that LGSN drives dedifferentiation in both GCSC and non-GCSC cells.

### LGSN promotes EMT and the acquisition of CSC phenotypes in gastric epithelial cells

**Fig. 2:**
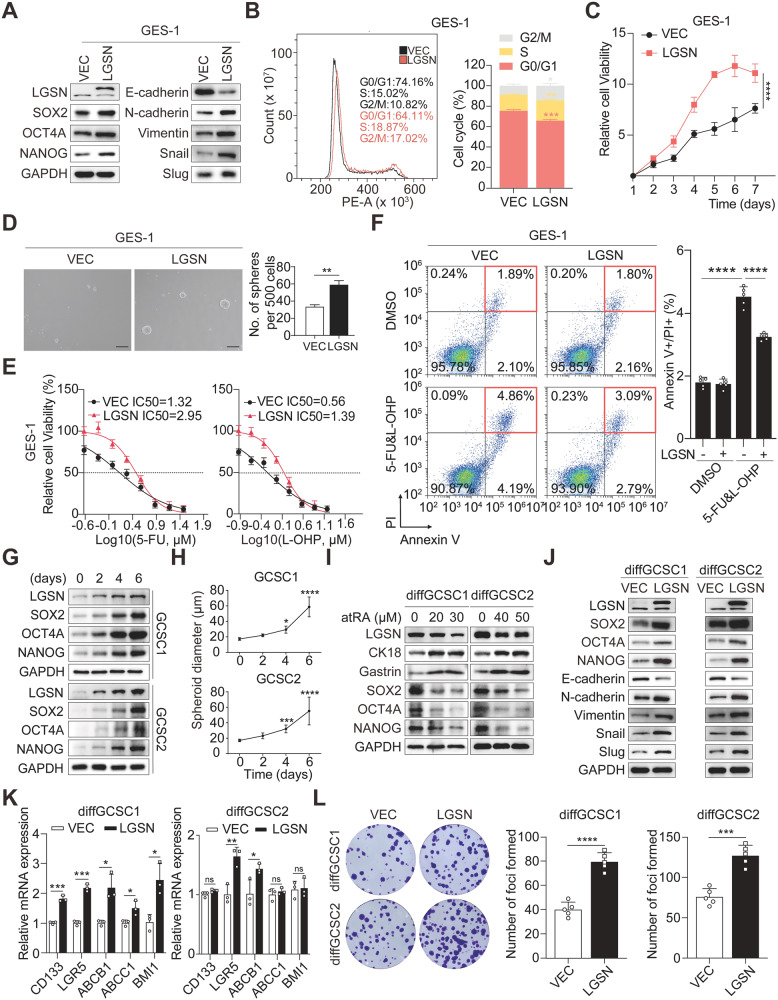
LGSN overexpression induces stemness in GES-1 cells and GCSCs. **A** Expression of LGSN and stemness- and EMT-related markers, as examined by western blot, in VEC (vector) and *LGSN*-overexpressing GES-1 cells. **B** Representative flow cytometric histograms and quantification of cell cycle distribution in VEC and *LGSN*-overexpressing GES-1 cells. **C** Cell proliferation CCK-8 assay of VEC and *LGSN*-overexpressing GES-1 cells. **D** Tumor spheroid formation assay showing the renewal potential of VEC and *LGSN*-overexpressing GES-1 cells (n = 3); scale bars, 200 μm. **E** CCK-8 measurement of IC50 for 5-FU & L-OHP treatment of VEC and *LGSN*-overexpressing GES-1 cells at the indicated concentrations (μM) (Log10) (n = 3). **F** Representative scatter diagram and quantification ratio for VEC and *LGSN*-overexpressing GES-1 cells compared by flow cytometry with Annexin-V-fluorescein isothiocyanate and PI double-staining after treatment with DMSO or 1 μM 5-FU & 0.5 μM L-OHP for 12 h (n = 3). Time-course analysis of expression level of LGSN and stemness-related proteins (SOX2, OCT4A and NANOG) (**G**) and quantification of spheroid diameter (**H**) in GCSCs on days 0, 2, 4, and 6 (n = 3). **I** Western blot of LGSN, CK18, and stemness-related proteins in differentiated GCSCs (diffGCSCs) treated with indicated concentrations of atRA for 5 days. **J** Expression levels of LGSN and EMT- and stemness-related proteins in VEC and *LGSN*-overexpressing diffGCSCs. **K** Relative mRNA levels of stemness and drug-resistance-related genes in VEC and *LGSN*-overexpressing diffGCSC1 by real-time PCR (RT-qPCR) (n = 3). **L** Cell proliferation ability, as determined by colony formation assay, of VEC and *LGSN*-overexpressing diffGCSCs (n = 3). *P < 0.05; **P < 0.01; ***P < 0.001; ****P < 0.0001; error bars show mean ± SD.

After discovering the unexpected potency of LGSN’s influence on adult gastric epithelial cell dysfunction, we further observed that LGSN prominently increased the expression of EMT- and stemness-associated proteins (Fig. 2A). Recent studies suggested that the majority (66%) of gastric adenocarcinomas have at least one alteration to their cell cycle regulators [17]. As expected, *LGSN*-overexpressed GES-1 cells showed a significant increase in the percentage of G0/G1 and S-phase cells and, therefore, remarkably increased cell proliferation (Fig. 2B, C). We next examined LGSN-overexpression in GES-1 and found it promoted spheroid formation; cell migration; and resistance to 5-fluorouracil (5-FU), oxaliplatin (L-OHP), and cisplatin (DDP) therapy and decreased the percentage of annexin V+ and/or propidium iodide (PI)+ cells (Fig. 2D–F; Supplementary Fig. S5A, B). Taken together, these findings suggest that the overexpression of *LGSN* promotes the normal gastric epithelial cells to acquire an abnormal CSC-like phenotype, along with increased proliferation and migration abilities.

### LGSN promotes the reacquisition of stemness characteristics in GCSC-derived differentiated GC cells

We next sought to extend our investigation into the potential role of LGSN in GCSC maintenance. We found that the LGSN protein gradually accumulated during the GSCS self-renewal process, in which spheroid size was observed to increase in a time-dependent manner, accompanied by the up-regulation of self-renewal transcription factors (Fig. 2G, H; Supplementary Fig. S6). These findings indicate that the continuously accumulating LGSN controls GCSC stemness maintenance.

Several recent studies have demonstrated that non-CSCs also acquire stemness and revert back to CSCs to facilitate their adaption to tumor niche stress under certain conditions [18]. To investigate the reacquisition of the characteristic stemness differentiation potential of GCSCs, western blot analysis was performed to show that all-trans retinoic acid (ATRA) treatment effectively induced the differentiation of GCSCs with increasing the levels of CK18 and Gastrin, and decreasing those of stemness-associated proteins, which was accompanied by LGSN protein degradation (Fig. 2I). This data demonstrated that LGSN positive GCSCs have strongly differentiation potential. After that, we kept atRA treatment for a month and gained two GCSC-derived differentiated GC cells (defined here as diffGCSC1 and diffGCSC2). Further observation revealed that *LGSN* overexpression led to the re-augmentation of EMT- (E-cadherin, N-cadherin, Vimentin, Snail and Slug) and stemness-associated indicators (SOX2, OCT4A, NANOG, CD133 and LGR5), drug efflux proteins ATP-binding cassette (ABCB1 and ABCC1), and hedgehog signal marker (BMI1) expression (Fig. 2J, K). In addition, exogenous expression of *LGSN* in diffGCSCs exerted dramatic promotional effects on cell proliferation and migration (Fig. 2L; Supplementary Fig. S7A, B). Thus, aberrant *LGSN* overexpression is considered to be responsible for the EMT process and the maintenance of GCSC stemness.

### Vimentin interacts with LGSN and promotes LGSN-induced tumorigenesis

**Fig. 3:**
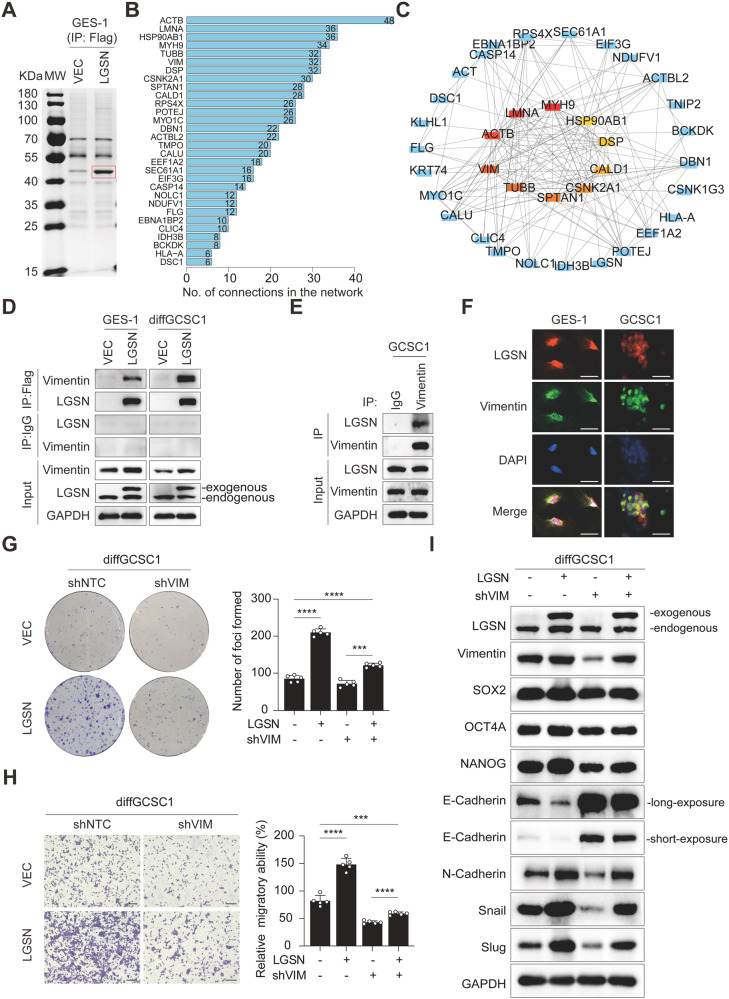
LGSN interacts with vimentin to promote tumorigenicity and EMT in GCSCs. **A** Coomassie blue staining showing Flag-immunoprecipitated proteins from *LGSN*-overexpressed GES-1 cell lysates. The protein indicated by the red rectangle was further analyzed by protein mass spectrometry. Parallel immunoprecipitation using VEC-overexpressing GES-1 cells was performed as a negative control. **B** Bar plot shows the top 30 most interconnected differentially expressed proteins (presented as corresponding gene names) identified in (**A**). **C** The hub proteins (presented as corresponding gene names) are among those identified to interact with LGSN. **D** Interaction between Flag-tagged LGSN and vimentin, as determined by Flag-pull-down and western blot. **E** Co-immunoprecipitation of endogenous LGSN and vimentin pull-down from GCSC1. **F** Representative dual-color immunofluorescent analysis of cells from GES-1 and GCSC1 spheroids showing the colocalization of vimentin (green) and LGSN (red); scale bars, 25 μm. Rescue experiments showing cell proliferation (*n* = 3) (**G**) and migration ability (*n* = 3) (**H**) of *LGSN*-overexpressing differentiated GCSC1 cells transfected with or without sh*VIM* plasmids; scale bars, 200 μm. **I** Western blot of indicated proteins in *LGSN*-overexpressing diffGCSC1 cells transfected with or without sh*VIM* plasmids (72 h). ***P < 0.001; ****P < 0.0001; error bars show mean ± SD.

To look for the molecular mechanisms underlying the effects on cell stemness and EMT conferred by LGSN, we initially performed co-immunoprecipitation (Co-IP) and Flag-tagged pull-down assay to identify LGSN-targeted proteins, which has a molecular weight of approximately 40–55 kDa (differential binding in red rectangle) (Fig. 3A; Supplementary Fig. S8A, B). We found a regulatory network with key enriched molecular functions of “cell adhesion molecular binding” and “structural constituent of cytoskeleton”, and a number of putative protein-protein (converted into indicated gene names) interactions for the 40 DEGs were predicted using the STRING database (Fig. 3B; Supplementary Fig. S8C, D, Table S5). The top 10 hub genes among these included *VIM* (gene name of vimentin), which was a clear overlapping prediction of both STRING and Cytoscape (Fig. 3C; Supplementary Fig. S8E). Previous work showed that LGSN may act as a component of the cytoskeleton itself [13]. Meanwhile, vimentin is also one of the key factors contributing to the actin cytoskeleton reorganization in invasive/metastatic GC cells [19] and is significantly associated with poor overall survival, first progression, and progression-free survival in GC patients (Supplementary Fig. S8F), indicating vimentin could be the most likely functional LGSN-binding candidate involved in cell stemness maintenance and the most suitable for further investigation.

Initially, Co-IP assays confirmed the direct binding of vimentin to LGSN in GES-1 or *LGSN-*overexpressing diffGCSC1 cells (Fig. 3D, E). Moreover, the immunofluorescence co-staining results showed LGSN and vimentin colocalized primarily at the cell cytoplasm in GES-1 and GCSC cells (Fig. 3F). To further understand the interplay between LGSN and vimentin expression, we found that *VIM* expression was consistently and significantly up-regulated with *LGSN* expression in gastric tumor tissues from dataset GSE29272 (Supplementary Fig. S8G, H). According to the intracellular signal transduction cascade effect, we proposed that vimentin may be a downstream molecule of LGSN in the process of carcinogenesis. We next tested if tumorigenesis, proliferation, or cell migration would be impeded by *VIM* depletion; in contrast, overexpressing *LGSN* in *VIM*-depleted cells rescued proliferation (Fig. 3G, Supplementary Fig. S9A) and promoted cell migration (Fig. 3G, H; Supplementary Fig. S9A, B). In line with the *VIM* knockdown verification, migration ability was dramatically reduced in diffGCSC1 following treatment with Withaferin A (WFA), a vimentin filament fragmentation inducer [20], whereas overexpressing *LGSN* partially reversed the inhibitory effects of WFA treatment (Supplementary Fig. S9C). We also obtained evidence that vimentin may first need to be phosphorylated at S56 before it responds to LGSN (Supplementary Fig. S9D, E). Furthermore, *VIM* downregulation impaired the EMT-promotion effects of *LGSN* overexpression (Fig. 3I), whereas overexpression of *LGSN* in *VIM*-downregulated cells had little effect on the recovery of stemness-associated proteins in the early stages of cellular response, indicating that EMT precedes stemness in LGSN-mediated biological process. Collectively, these results demonstrated that LGSN promotes vimentin-mediated EMT and stemness maintenance, which are directly associated with vimentin binding.

### LGSN interference attenuates GCSC carcinogenesis

**Fig. 4:**
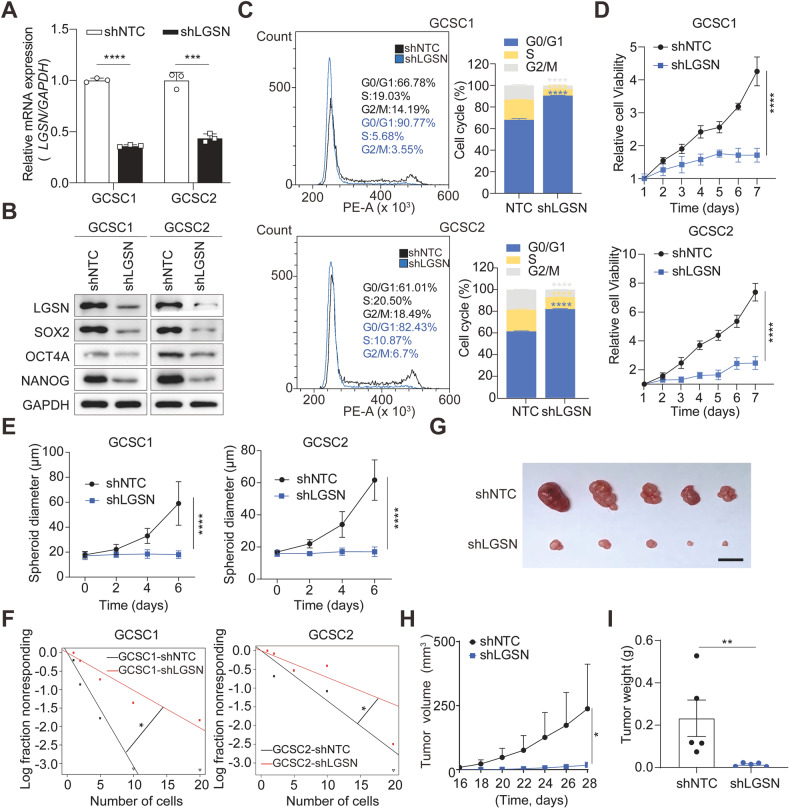
*LGSN* knockdown inhibits the growth of GCSCs in vitro and in vivo. **A** Relative mRNA levels of *LGSN* in shNTC (scrambled shRNA nontarget control) and sh*LGSN (LGSN*-silenced) GCSCs, as detected by RT-qPCR (*n* = 3). **B** Expression levels of LGSN and stemness-related proteins in shNTC and sh*LGSN* GCSCs detected by western blot. **C** Flow cytometry assay showing cell cycle in shNTC and sh*LGSN* GCSCs (*n* = 3). **D** CCK-8 determination of cell viability in LGSN-knockdown GCSCs (*n* = 3). **E** Tumor spheroid formation assay results for the spheroid diameter of shNTC and sh*LGSN* GCSCs on days 0, 2, 4, and 6 (*n* = 3). **F** In vitro limiting dilution assay results for the frequency of shNTC and sh*LGSN* GCSCs (*n* = 12). **G–I** Subcutaneous in vivo xenograft formation in nude mice injected with GCSCs transfected with shNTC or sh*LGSN*. Representative images are shown; scale bar, 1 cm (**G**). Tumor size was monitored every 2 days (*n* = 5) (**H**). Tumor weight detected at the end of the experiment (*n* = 5) (**I**). *P < 0.05; **P < 0.01; ***P < 0.001; ****P < 0.0001; error bars show mean ± SD.

LGSN expression is almost completely silenced in normal adult human tissues [11]. Thus, we speculated that the dynamic emergence of the trans-differentiation process of GCSCs is regulated by LGSN, and this was supported by our observation that stemness proteins SOX2, OCT4A and NANOG were significantly decreased in *LGSN*-knockdown GCSCs by western blot analysis (Fig. 4A, B). We then discovered that LGSN-knockdown distinctly induced cell cycle arrest at the G1/S phase and diminished G2/M phase transition, thereby constraining the proliferation of GCSCs cells (Fig. 4C, D). Similarly, LGSN-silenced GSCSs displayed markedly weakened sphere formation and self-renewal abilities (Fig. 4B; E, F; Supplementary Fig. S10). Next, we confirmed in vivo that mice bearing LGSN-silenced GCSC cells had significantly slower tumor growth (Fig. 4G–I, Supplementary Fig. S11). These data indicated that LGSN holds the possibility as a therapeutic target.

### LGSN interference triggered pyroptotic cell death by vimentin depletion in GCSCs

**Fig. 5:**
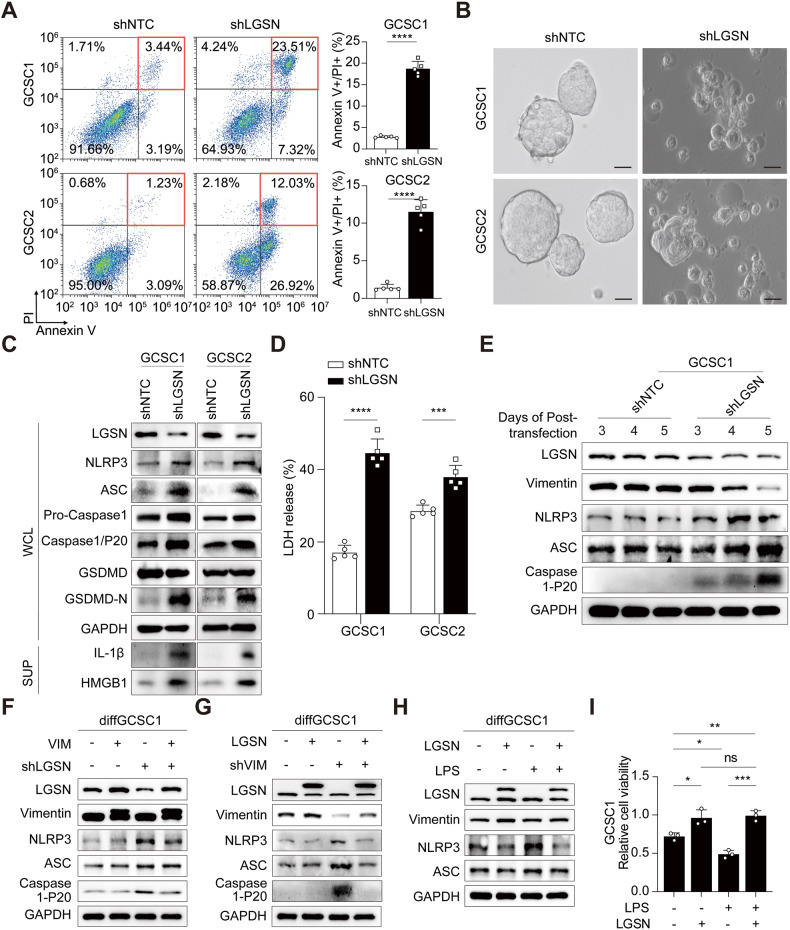
LGSN expression regulates pyroptosis in GCSCs by mediating NLRP3/ACS/caspase1. **A** Flow cytometry analysis of the Annexin V/PI double-positive subpopulations of shNTC and sh*LGSN* GCSCs. **B** Representative images of the morphology of shNTC and sh*LGSN* GCSCs; scale bar, 25 μm. **C** Western blot showing indicated protein levels in whole cell lysate (WCL) and supernatant protein lysate (SUP) products of shNTC and sh*LGSN* GCSCs. **D** LDH-release assay of shNTC and sh*LGSN* GCSCs (*n* = 3). **E** Time-course analysis of indicated protein levels in shNTC and sh*LGSN* GCSC1. **F** Western blot showing indicated protein levels in *VIM*-overexpressing diffGCSC1 cells transfected with or without sh*LGSN* plasmids. **G** Indicated protein levels of *LGSN*-overexpressing diffGCSC1 transfected with or without sh*VIM* plasmids on western blot. **H** Cell viability detection by CCK-8 in VEC and *LGSN*-overexpressing GCSC1 treated with LPS (1 μg/ml; 6 h) (*n* = 3). **I** Western blot showing indicated protein levels in LPS-treated (1 μg/ml; 24 h) *LGSN*-overexpressing GCSC1. ns, not significant; *P < 0.05; **P < 0.01; ***P < 0.001; ****P < 0.0001; error bars show mean ± SD.

To understand why the down-regulation of LGSN affected the self-renewal and tumorigenicity of GCSCs, we used *LGSN* interference, which significantly increased GCSC apoptosis (Fig. 5A). The dying cells exhibited evident swelling, with characteristic large bubble-like protrusions appearing on their plasma membranes, strongly indicating they experienced a morphological change characterized as pyroptosis (Fig. 5B) [21].

Qualitatively distinct from apoptosis, pyroptosis involves the inflammasome [22, 23]. Indeed, we found that *LGSN* knockdown promoted NACHT, LRR, and PYD domain-containing protein 3 (NLRP3) and apoptosis-associated speck-like protein containing a CARD (ASC) protein expression, along with pro-caspase1 accumulation, caspase1-P20 activation, and gasdermin D N-terminal (GSDMD-N) increase, as well as the consequent release of interleukin (IL)-1β, high mobility group box 1 (HMGB1), and lactate dehydrogenase (LDH) from the cell membrane (Fig. 5C, D). Intriguingly, time-course analysis of LGSN knockdown in GCSC cells showed gradually increased NLRP3, ASC, and caspase1-P20 protein levels along with vimentin degradation (Fig. 5E), and vimentin reconstitution reversed the inhibitory effect of LGSN repression (Fig. 5F). *LGSN* down-regulation also impaired tumorigenesis, cell proliferation, and migration, which were associated with a decrease in endogenous vimentin expression, while ectopic vimentin expression reversed the inhibitory effects of LGSN repression (Supplementary Fig. S12A–C). Similarly, pyroptosis-like swelling with membrane bubbles was seen in GCSCs that underwent WFA treatment (Supplementary Fig. S12D). Furthermore, the depletion of vimentin induced NLRP3 expression (Fig. 5G), suggests that vimentin is essential for LGSN-interference-induced pyroptosis.

To further analyze these phenomena, *LGSN*-overexpressed diffGCSCs were subjected to treatment with lipopolysaccharide (LPS), a known NLRP3 inflammasome activator (32, 33). As expected, LPS stimulation upregulated the expression of NLRP3 and ASC in differentiated GCSCs while *LGSN* overexpression attenuated LPS-induced pyroptosis signals and promoted the malignant proliferation of GCSCs (Fig. 5H, I). Collectively, our data suggested that LGSN acts as a powerful repressor of pyroptosis in GCSCs via upregulating vimentin.

### Loss of LGSN sensitized GCSCs to the cytotoxic effects of chemotherapy by expediting pyroptotic cell death

**Fig. 6:**
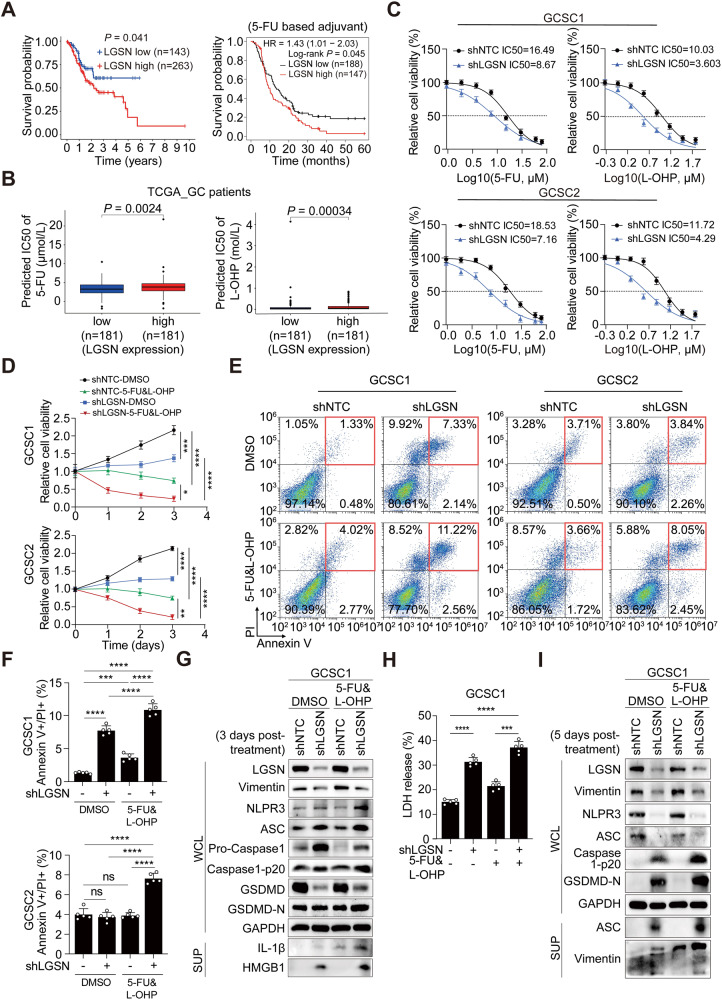
*LGSN* interference causes chemotherapeutic sensitivity by inducing pyroptosis in GCSCs. **A** Overall survival (OS) of GC patients with high and low LGSN expression according to a maximally selected rank combination of algorithms after clinical pharmaceutical therapy in the TCGA cohort (left) and post-5-FU treatment in Kaplan-Meier Plotter database (right). **B** Boxplots of IC50 indicating the predicted clinical 5-FU (left)- and L-OHP (right)-sensitivity of high- (*n* = 181) and low- (*n* = 181) LGSN-expressing GC patients in the TCGA cohort. **C** CCK-8 assay of IC50 values for 5-FU (left) and L-OHP (right) for shNTC and sh*LGSN* GCSC1 (top) and GCSC2 (bottom) (μM) (Log10) (*n* = 3). **D** Cell viability, as detected by CCK-8, of shNTC and sh*LGSN* GCSCs treated with 2.5 μM 5-FU plus 1 μM L-OHP for 24 h (*n* = 3). **E–F** Percentage of Annexin V/PI double-positive subpopulation, as detected by flow cytometry analysis of shNTC and sh*LGSN* GCSCs treated with 2.5 μM 5-FU plus 1 μM L-OHP for 12 h (*n* = 3). **G** Immunoblot of indicated protein levels in whole-protein lysis and supernatant protein lysis products of shNTC and sh*LGSN* GCSCs (after transfection for 24 h) treated with 5-FU plus 2.5 μM 5-FU plus 1 μM L-OHP for 3 days. **H** LDH activity characterization of *LGSN*-knockdown GCSCs treated with 5-FU plus L-OHP (*n* = 3). **I** Immunoblot of indicated protein levels in WCL and SUP products of shNTC and sh*LGSN* GCSCs (after transfection for 24 h) treated with 2.5 μM 5-FU plus 1 μM L-OHP for 5 days. ns, not significant; *P < 0.05; **P < 0.01; ***P < 0.001; ****P < 0.0001; error bars show mean ± SD.

We next questioned whether repressing LGSN in GCSCs could confer new therapeutic vulnerabilities. We first attempted to rule out the possibility of cell death in normal cells by silencing *LGSN*. The impact of LGSN interference on the cell death of normal gastric epithelial cells (GES-1) and normal intestinal epithelial cells (NCM460) was evaluated, respectively. Interestingly, we did not observe any substantial influence on cell death upon *LGSN* depletion (Supplementary Fig. S13A, B), indicating that *LGSN* is a safe and potential therapeutic target for GC patients. Using the maximally selected rank (MSR) algorithm from the “maxstat” R package, we calculated that high*-LGSN-*expressing patients had a generally worse prognosis under pharmaceutical therapy, which indicated that LGSN promotes the resistance of GCSCs to chemotherapeutic regimes such as 5-FU and radiation therapy (Fig. 6A; Supplementary Fig. S14A). In particular, we determined the IC50 values of 5-FU (Fig. 6B; Supplementary Fig. S14B), cisplatin (Supplementary Fig. S14C), L-OHP (Fig. 6B), and Metformin (Supplementary Fig. S14D) (clinically approved drugs may induce pyroptosis in cancers [24]) in LGSN-high and LGSN-low gastric cancer patients in TCGA database. This prediction indicated that high levels of LGSN are associated with poor survival in GC patients given pharmaceutical therapy, possibly due to LGSN overexpression preventing pyroptosis, resulting in chemoresistance. Therefore, there is an urgent and unmet need for more effective LGSN-targeting GC treatments in the clinical arena.

We thus investigated whether the pyroptotic membrane pores that emerged in GCSCs after LGSN expression was targeted could enhance chemo drug uptake or flux. We found that LGSN silencing sensitized the chemotherapy response and suppressed the proliferation ability of GCSCs and increased the percentage of annexin V+ and/or PI+ cells (Fig. 6C–E; Supplementary Fig. S14E–S15). Mechanically, the silencing of LGSN expression combined with treatment with 5-FU and L-OHP also attenuated vimentin expression and promoted the protein expression levels of caspase1-P20 and GSDMD-N, and LDH releases by GCSCs (Fig. 6F–H). Whereas single 5-FU and L-OHP treatments did not trigger similar pyroptotic signals. Interestingly, both ACS and vimentin protein levels were significantly increased in the supernatant of *LGSN*-knockdown cells with prolonged chemo-drugs treatment compared with those cells without combined chemotherapy (Fig. 6I), indicating that cell rupture occurred a long time after LGSN knockdown followed by vimentin disruption, and this effect could be accelerated by combined chemotherapy treatments.

### LGSN interference improved chemotherapy via pyroptosis induction in vivo

Given the crucial role of LGSN in regulating 5-FU and L-OHP resistance, we reasoned that genetically manipulating *LGSN* might push cancer cells toward the pyroptotic threshold, allowing cumulative lethal damage by chemotherapy and eventually the killing of cancers consisting of CSCs. Therefore, we designed xenograft-tumor-bearing mouse therapeutic models to evaluate GCSC susceptibility to pharmaceutical intervention after *LGSN*-knockdown and the lessening of cancer severity in vivo.

**Fig. 7:**
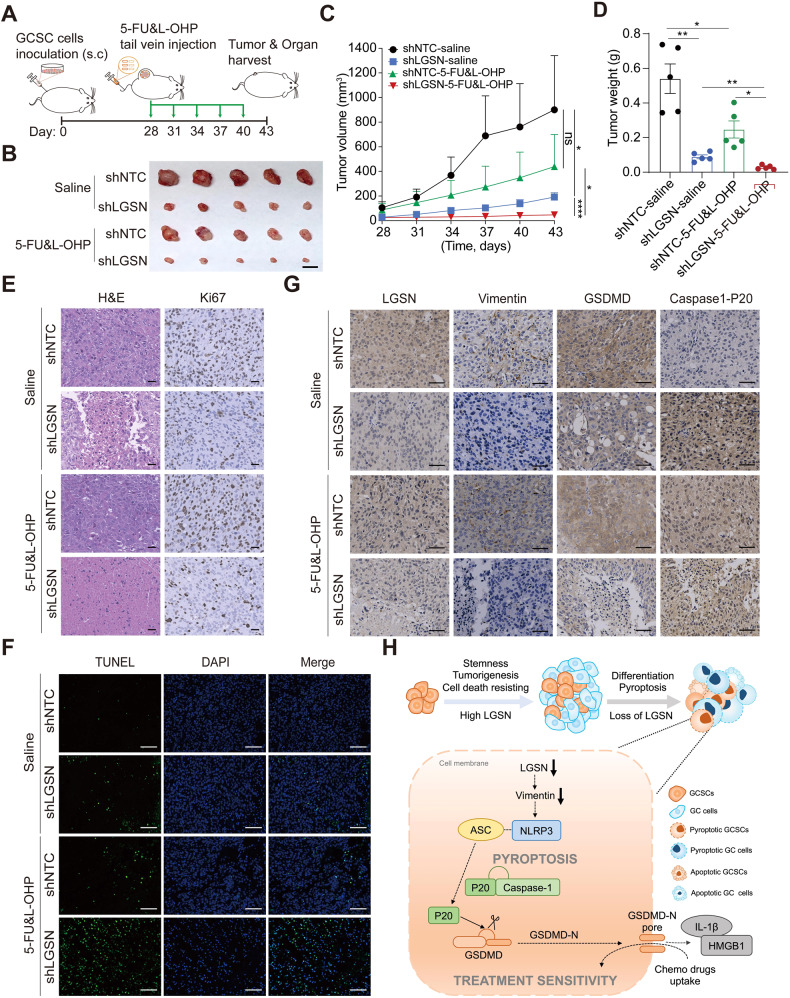
LGSN knockdown in GCSC markedly enhanced chemosensitivity in an in vivo model. **A** Schematic of the chemo-drug intervention program with subcutaneously implanted xenografts of shNTC and sh*LGSN* GCSC1 (after transfection for 36 h) in nude mice. shNTC and sh*LGSN* GCSC1 cells were subcutaneously injected into mice. After 28 days, the mouse groups (shNTC and sh*LGSN*) (*n* = 5) were injected intraperitoneally with saline or 5-FU (10 mg/kg/mouse) & L-OHP (5 mg/kg/mouse) every 3 days. Fifteen days after injection, the mice were euthanized, and the growth of the tumor was evaluated. **B** Photograph of shNTC and sh*LGSN* GCSC1 xenograft tumors from mice given indicated treatment; scale bar, 1 cm. **C** Tumor growth curves show development of xenografts of shNTC and sh*LGSN* GCSC1 treated with chemotherapeutic drugs (*n* = 5). **D** Tumor weight detected at the end of the experiment (*n* = 5). **E** Representative H&E staining images of tumors confirming malignant phenotype, and Ki67 labelling showing tumor proliferation; scale bars, 50 μm. **F** Micrograph images of TUNEL assay showing apoptosis of xenograft tumors in mice; scale bars, 100 μm. **G** Representative IHC staining images for LGSN, Vimentin, GSDMD, and Caspase1-P20 in mouse xenograft tumors; scale bars, 50 μm. **H** Schematic diagram showing proposed working model for the regulation of LGSN-mediated stemness and pyroptosis, facilitating sensitivity to chemotherapy in gastric cancer stem cells. ns, not significant; ns, not significant; *P < 0.05; **P < 0.01; ****P < 0.0001; error bars show mean ± SD.

Nude mice inoculated with control shRNA-NTC or *LGSN*-knockdown GCSC-derived xenograft tumors were given either normal saline or a combination of both 5-FU and L-OHP for 15 days (Fig. 7A). We found that *LGSN*-repressed xenografts were smaller in volume, and the 5-FU and L-OHP treatment groups showed mild tumor suppression. Moreover, the combination of repressed LGSN expression and chemo-drugs treatment dramatically inhibited tumor growth compared with the mild tumor suppression of the 5-FU and L-OHP treatments without LGSN suppression (Fig. 7B–D). The amount of severely apoptotic and necrotic cell death GC cells significantly increased, consistent with the significant reduction in tumor proliferation and pyroptosis markers, in both the sh*LGSN* group and combination treatment group, suggesting *LGSN*-knockdown-mediated vimentin depletion effectively triggered pyroptotic cell death signaling and promoted chemotherapeutic effects on the gastric tumor in vivo (Fig. 7E–G). There was no significant difference in mice body weight (Supplementary Fig. S16A) and major vital organs were observed (Supplementary Fig. S16B).

To further assess whether *LGSN* could be a safe and potential target for GC therapy, we performed systemic treatment in nude mice bearing GCSC xenografts via tail vein injection of GFP-tagged AAV-sh*LGSN* and AAV–shNTC, respectively. The AAV-sh*LGSN* treatment was found to effectively inhibit tumorigenicity in GCSC xenograft-bearing mice, without causing any changes in body weight, as compared to the AAV-shNTC treatment (Supplementary Fig. S17A–D; Supplementary Fig. S18A). Moreover, both sh*LGSN* and control viruses can reach GCSC xenografts (Supplementary Fig. S17E) and normal mouse tissues (Supplementary Fig. S18B), but not the normal lens where LGSN is exclusively expressed (Supplementary Fig. S17E). Importantly, the *LGSN* interference does not cause histological changes in the normal vital organs, including the eyes (Supplementary Fig. S17F; Supplementary Fig. S18B). Collectively, these results suggested that a combination of traditional chemotherapy drugs and LGSN depletion may be a safer and more effective treatment approach for GC.

## Discussion

The GC tumor environment is incredibly heterogeneous, as it encompasses cells of different lineages and levels of differentiation. CSCs are emerging as critical players in initiating and maintaining tumors [25], and dedifferentiation can allow for the emergence of CSCs [26]. However, the stemness determinates that control GCSC generation are still not fully understood. In this study, we first showed that LGSN is overexpressed in GC and is involved in maintaining the stemness of CD44^+^/CD54^+^ GCSCs in those with an unfavorable prognosis. CD44 and CD54 are widely expressed in tumor, stromal, and immune cells [15]. Although LGSN splicing variant 4 has a reportedly high expression in lung cancer, no data have been gathered showing that LGSN is involved in the lung cancer stemness [11]. Thus, a potential research direction opened up as to whether the addition of LGSN can further narrow the range of GC-specific CSC populations and be used to precisely identify the heterogeneous gastric isthmus, a long-lived stem cell population. Additionally, LGSN was found to promote GCSCs’ high stemness state via the induction of vimentin, and a previous study showed that full-length LGSN can bind the 2B filament region of vimentin in the lens [12]. Vimentin intermediate filaments orchestrate microtubule patterning and the alignment of traction stresses [27] to allow for directional migration in polypoidal giant cancer cells [28]. As such, we proposed that LGSN can regulate gastric epithelial cancer cell heterogeneity and the invasiveness of subpopulations in a bidirectional interconversion process.

Intratumoral sub-populations of CSCs are often able to use differential mechanisms [29] to facilitate both tumor initiation and maintenance [30] and seed new therapeutic-resistant tumors. The ability to undergo and govern RCD is one of the most prominent intrinsic CSC-specific characteristics. As we have only begun to scratch the surface of how these immortal CSCs cells drive stemness and tumorigenesis, very little is known about the specific death evasion modules utilized by these GCSCs. In our study, we obtained evidence that GCSC-specific LGSN up-regulates and hijacks vimentin to override the NLRP3-dependent pyroptotic program, switching the fate of GCSCs from death to proliferation and maintaining their self-renewal ability. This mechanism may be one of the key pathways in the death resistance of GCSCs. Once *LGSN* was knocked down, there was a significant depletion of vimentin followed by NLRP3/caspase1-P20/GSDMD-N-mediated pyroptosis, which potentially prevents self-renewal and tumorigenesis. Consistent with this, some extrinsic and intrinsic death pathways of LGSN have been shown to participate in controlling lens fiber cell terminal differentiation [31] in the control of normal lens development and prevention of cataractogenesis [32], indicating that this property of LGSN to regulate the process of cell death is generally conserved in human cells.

Previously, vimentin was reported to modulate cell fate and was then found to be cleaved by caspases in apoptotic neutrophils [33]. The cleavage of vimentin and loss of intermediate filaments are key features of pyroptotic cell swelling [34]. Actin filaments disruption-induced cytoskeletal defects also elicit Pyrin/caspase-1 inflammasome activation and pyroptosis in mouse macrophages [35]. However, there is an unresolved issue as to how vimentin mediates inflammasome activation. Findings inconsistent with this were obtained in several studies, in which *VIM*-knockout inactivated NLRP3 in macrophages [36] and loss of vimentin inhibited the activation of NLRP3 inflammasome signaling, leading to lung inflammation and leaky endothelium and alveolar epithelial barriers [37]. Notably, as shown in Fig. 5E, NLRP3 gradually increased within 4 days post *LGSN* repressing while it started to decrease 5 days post *LGSN* repressing in GCSC cells, indicating LGSN probably confers influence on the early stage of pyroptosis. However, other studies have suggested that vimentin-deficient cells should have more robust inflammasome activation, which has been linked to the mitochondrial-derived ROS generation [38]. Interestingly, vimentin intermediate filaments are involved in modulating mitochondrial motility, and a lack of vimentin increased ROS production [39], leading to the activation of NLRP3 inflammasome [40]. Due to the complexity of crosstalk among microbiota, organic barriers, and the immune system, the specificity of vimentin in the regulation of inflammation in vivo is likely to differ among models [41]. This is in line with our findings that NLRP3 expression is dependent on a lack of vimentin. Conceivably, further work on the relationship between LGSN, vimentin, and ROS will provide insights into the mechanisms of NLRP3 inflammasome activation in various types of GCSCs and GCs.

Pyroptotic cell death appears to share significant cross-talk with other RCD modes, particularly apoptosis [42]. However, apoptotic cells that result from therapy may release signals [43] that can impact nearby cells, driving tumor repopulation after therapies and potentially promoting evasion from antitumor immunity [44]. Thus, alternative approaches to target and induce other RCD modes, such as pyroptosis, have been explored as potential means to eliminate cancer cells [45].In recent years, clinicians have started to use a handful of pyroptosis-inducing therapeutic strategies in the fight against CSCs [46]. Yet, pyroptosis induction alone could fail to trigger efficient tumor inhibition, highlighting the importance of treating the majority of tumors with a combination of pyroptosis inducers and other therapies [47]. Despite several studies reporting the role of cancer chemotherapy in cell sensitivity to pyroptosis induction [48], the association between pyroptosis and anticancer chemotherapy in GCs remains unclear. Recent studies have demonstrated that GSDMD pores are an average of 22 nm in diameter [49], which may not allow very large ASC specks (inflammasomes), at 1–2 μm in diameter, to penetrate. Interestingly, we found that ACS was released and vimentin was cleaved outside of larger GSDMD-N membrane holes after LGSN knockdown, indicating that a genetic absence of LGSN may permit chemo-drug influx through GSDMD pores by synergizing with a reduced dosage of 5-FU and L-OHP, leading to dramatic antitumor effects and significant xenotransplanted tumor regression. This synergistic effect could alleviate the chemo drugs side effects on normal vital organs. LGSN expression is almost absent in normal adult human tissues [11], which makes it an ideal target for GC treatment. However, it’s worth mentioning that LGSN is expressed exclusively at high levels in the transparent, not the cataractous human lens [50]. Although the dramatically decreased LGSN mRNA has a strong potential connection with age-related cataracts [51], the normal lens was completely immune-privileged and could be protected from the blood–organ barrier [52]. It has also been found that in order to maintain lens transparency, LGSN mRNA is tightly regulated and rapidly diminished in fully differentiated mature lens secondary fibrocytes as they near their final stage of differentiation [9, 12]. Moreover, a previous study revealed that anti-LGSN immunological response in lung cancer patients is not harmful to the lens [11]. In line with previous findings, our systemic in vivo AAV-sh*LGSN* treatment study also showed that sh*LGSN* viruses are unable to reach the lens where LGSN is exclusively expressed. Thus, LGSN is considered a promising molecular target for GC therapy. Future experiments are needed to determine how the accompanying tumor microenvironment of GC responds to LGSN-mediated cell death evasion and to investigate more appropriate systematic therapeutic strategies.

Taken together, our findings revealed that LGSN can serve as a switch for GCSC-specific stemness and cell death. LGSN may result in blunted extrinsic stress or cytoplasmic homeostasis perturbations that aid in resisting pyroptosis priming and activation. Targeting LGSN contributed to vimentin-loss-mediated pyroptosis, which improved the susceptibility of GC cells to conventional chemo-drugs (Fig. 7H). Our results highlighted the importance of the tumor-initiation role of LGSN and the exact pyroptotic machinery affected in GCSCs and provided insights into the best chemoprevention strategies and most efficacious targeted therapeutic interventions.

## Supplementary information

Li et al_Supplementary Information

Li et al_Supplementary Figures

Li et al_Author Contributions Statement

Li et al_Supplementary_Table 1

Li et al_Supplementary_Table 2

Li et al_Supplementary_Table 3

Li et al_Supplementary_Table 4

Li et al_Supplementary_Table 5

Li et al_Supplementary_Table 6

Original Data File

## Data Availability

The RNA sequencing raw data generated from this study have been submitted to the Gene Expression Omnibus database (GEO; accession number: GSE210249). GSE29272 dataset was available in the GEO. The remaining data are available within the Supplementary Information.
