# Supplementary material for: Targeting LGSN restores sensitivity to chemotherapy in gastric cancer stem cells by triggering pyroptosis
Source: Cell Death Dis. 2023 Aug 23;14(8):545. doi: 10.1038/s41419-023-06081-8 (PMC10447538; doi:10.1038/s41419-023-06081-8)
Supplement: Supplementary file 1 — Li et al_Supplementary Information [file 41419_2023_6081_MOESM1_ESM.docx]

**Targeting LGSN restores sensitivity to chemotherapy in gastric cancer stem cells by triggering pyroptosis**

Yu-Ting Li^1,2,*^, Xiang-Yu Tan^1,*^, Li-Xiang Ma^1^, Hua-Hui Li^1,2^, Shu-Hong Zhang^1^, Chui-Mian Zeng^3^, Liu-Na Huang^1^, Ji-Xian Xiong^1,#^, Li Fu^1^^,#^

^1^Guangdong Province Key Laboratory of Regional Immunity and Diseases, Department of Pharmacology and International Cancer Center, Shenzhen University Medical School, Shenzhen University, Shenzhen, Guangdong, 518055, China;

^2^Shenzhen University-Friedrich Schiller Universität Jena Joint PhD Program in Biomedical Sciences, Shenzhen University Medical School, Shenzhen, Guangdong, 518055, China;

^3^Department of Endocrinology and Diabetes Center, The First Affiliated Hospital of Sun Yat-Sen University, Guangzhou, China;

*These authors contributed equally to this work

﻿**^#^Correspondence:** Li Fu, Department of Pharmacology and International Cancer Center, Shenzhen University Medical School, Shenzhen University, Shenzhen, Guangdong, 518055, China; Tel: +86-755-86671992; Email: gracelfu@szu.edu.cn; Ji-Xian Xiong, PhD, School of Pharmacy and International Cancer Center, Shenzhen University Medical School, Shenzhen University, Shenzhen, Guangdong, 518055, China; Tel/Fax: +86-755-86670623; E-mail: xiongjixian@szu.edu.cn

**Running title**: Targeting LGSN inhibits gastric cancer progression

**Conflict of interest:** The authors declare no potential conflict of interest.

**Keywords:** LGSN, stemness, pyroptosis, chemoresistance, gastric cancer

**Supplementary Materials and Methods**

**Reagents and antibodies**

The details of all the reagents (e.g., Oxaliplatin, 5-Fluorouracil, and Withaferin A) and antibodies (e.g., LGSN, vimentin, and GSDMD) used in this study were listed in Table S6.

**Immunoprecipitation**

Cells were washed with ice-cold PBS and lysed with immunoprecipitation lysis buffers (Invitrogen) containing a protease inhibitor cocktail (Mass Spectrometry-SAFE; Beyotime). Approximately 1000 mg of the LGSN- or vimentin-associated proteins were immunoprecipitated with protein A/G-agarose beads (Invitrogen) coupled with an anti-DYKDDDDK Tag (Cell Signaling) or anti-vimentin antibody (Cell Signaling) overnight at 4°C. A matched isotype antibody (IgG; Santa Cruz Biotechnology) was used as a negative control. After washing five times with lysis buffer, precipitated antibody-protein-bead conjugates were extracted with sample loading buffer and boiled for 10 min, followed by SDS/PAGE and western blot analysis.

**SDS/PAGE followed by mass spectrometry**

Immunoprecipitates were separated on 10% SDS/PAGE gel and stained with Coomassie brilliant blue to differentiate IgG bands expressed in wild-type or LGSN-overexpressing cells. A volume of 0.5-1 mm^3^ of band at a mass of 50-70 KDa was cut from gels. These bands were in-gel digested overnight with 100 ng of trypsin after destaining and dehydrating, eluted twice with 0.1% formic acid and 75% acetonitrile, and lyophilized. The lyophilized powder was then dissolved in 0.1% formic acid and loaded onto a C18 Nano-Trap column. The peptides were separated in a linear gradient elution on an analytical column and analyzed by Q Exactive HF-X mass spectrometer (Thermo Fisher). The resulting spectra data from each fraction were searched separately against the UniProt database [homo_sapiens_uniprot_2021_7_15. fasta. fasta (202195 sequences)] using the search engine Proteome Discoverer 2.2 (PD 2.2, Thermo).

**Spheroid formation assay**

Single cells used for tumor spheroid formation assays were seeded at a density of 100 cells per well into ultra-low attachment 6-well plates (Corning) with GCSC culture medium for 14-day culture, as previously described ^1^. Cells were replenished with fresh medium every 3 days. Spheroids were visualized under an inverted microscope (Zeiss). Tumor spheroids of diameters larger than 50 μm were counted. For statistical analysis, 500 cells from 5 wells were collected at a time and the process was repeated 3 times.

**Lentivirus vectors, cell transfection, and lentiviral transduction**

*LGSN* (NM_016571.3)- and *VIM* (NM_003380.5)-specific shRNA expression vectors, scrambled shRNA nontarget control (shNTC), overexpression, and control vectors were manufactured by Guangzhou IGE Biotechnology Ltd. The short hairpin RNA (shRNA) sequences used are listed in Table S6. Using Lipofectamine 3000 Reagent (Invitrogen) as instructed by the manufacturer’s protocol, envelope ﻿expressing plasmid pCMV-VSVG (﻿Addgene plasmid #8454; ﻿0.9 ﻿μg) and packaging plasmid psPAX2 (﻿Addgene plasmid #12260; ﻿9 ﻿μg) were co-transfected into ﻿95–99% confluent 293T cells (﻿100 mm Petri dish) with the custom lentiviral plasmids (﻿9 ﻿μg) to facilitate the harvesting of lentiviral particles. After 48 h, virus-containing supernatants were collected, filtered using a 0.45-μm filter, and concentrated in a Lenti-X Concentrator (Takara). To screen for stably transfected single clones, the target cells were infected with lentiviral particles mixed with 2.5 µg/mL polybrene and subsequently selected for using the antibiotic puromycin (5–10 µg/mL, Sigma). Target cells were prepared for real-time PCR (RT-qPCR) and western blot assay to verify the expression of targeted genes 48–72 h after transfection.

**Western blot**

Cultured cells were lysed in radio-immunoprecipitation buffer (MCE) supplemented with protease and phosphatase inhibitor cocktail (Roche). Total protein concentration was measured using BCA assay (Thermo Scientific). Cell lysates were separated on SDS-polyacrylamide gels (Epizyme), then transferred onto 0.22 μm polyvinylidene difluoride (Bio-Rad) membranes at 4°C. After the membranes were blocked in 5% BSA in Tris-buffered saline (TBST), 0.1% Tween-20 for 2 h, they were incubated with primary antibodies in 5% BSA in TBST overnight at 4°C and with secondary antibodies in TBST for 2 h at room temperature. The primary and secondary antibodies used in the western blot are listed in Table S6. Finally, the membranes were visualized using Pierce SuperSignal west chemiluminescent substrate (Thermo Scientific) and the ChemiDoc Touch Gel Imaging system (Bio-Rad). Images were quantified using Image Lab software (Bio-Rad). The uncropped blotting images were listed in Supplemental Material.

**RNA extraction and RT-qPCR**

Total RNA was isolated using TRIzol Reagent (Invitrogen) and converted into cDNA with PrimeScript RT-PCR Master Mix (Takara) in accordance with the manufacturer’s standard procedures. RT-qPCR for analyzing mRNA levels was conducted with TB Green Premix Ex Taq kit (Takara) on a CFX96 Real-Time PCR Detection System (Bio-Rad). The amplification procedure consisted of incubation at 95°C for 15 s, 60°C for 1 min, and 72°C for 1 min for 35 cycles. Data were then analyzed using the ΔΔCt method to calculate the fold-change normalized to cDNA of the housekeeping gene *GAPDH* in each sample. The forward and reverse primer sequences used are listed in Table S6.

**Flow cytometry**

For the cell death assay, cells were collected and stained with an Annexin V-fluorescein isothiocyanate (FITC)/Propidium Iodide (PI) Apoptosis Detection Kit (BD Biosciences) for 15 min on ice, in accordance with the manufacturer’s protocol, then detected and analyzed by flow cytometry using a CytoFLEX (Beckman Coulter).

For the cell cycle assay, we measured cellular DNA content using PI (Beyotime). Cells were collected by centrifugation ﻿at 1,000 ﻿x g and fixed with 70% ice-cold ethanol for 24 h at 4°C. Cells were washed twice with phosphate-buffered saline (PBS) and resuspended in a cell mixture with PI (final concentration at 5-50 μg/mL) before incubating at room temperature for 15 min in the dark. Finally, the cell cycles were analyzed by flow cytometry.

For CD44 or CD54 expression analysis, approximately 1 × 10^6^ cells were incubated on ice for 45 min with fluoresceinIsothiocyanate (FITC)-conjugated anti-CD44 (Biolegend) and allophycocyanin (APC)-conjugated anti-CD54 (Biolegend), which were 1:50 diluted according to instructions provided by the manufacturer. Cells were detected and analyzed by flow cytometry using a CytoFLEX machine.

**Cell proliferation assay**

The CCK-8 method was used to detect cell viability. Exponentially growing cells were plated in 96-well plates and the cell number adjusted to a density of cells per well of 2,000 (GES-1) or 5,000 (GCSCs)/well in 100 µL of DMEM medium. The absorbance was quantified using a microplate reader (Biotek) at 450 nm wavelength after cultivating cells with CCK-8 reagent (10 μL/well, Dojindo Kumamoto) and incubating for 4 h. The half-maximal inhibitory concentrations (IC50 values) were calculated using GraphPad Prism software.

**Colony formation assay**

Five hundred single cells were seeded into 6-well plates and incubated for 14 days. Colonies then were incubated with 4% paraformaldehyde for 15 min and stained with 1% crystal violet for another 20 min at room temperature. Cell colonies were counted (diameter ≥300 mm) ^2^ and analyzed by Image J software.

**Transwell migration assay**

In short, 2.5 × 10^4^ cells were mixed into 500 µL FBS-free medium and laid over the upper chamber (Falcon) of a 24-well plate. The lower chambers were filled with 700 µL of DMEM containing 10% FBS as a chemoattractant. Migrating cells on the lower side of the chambers were fixed with 4% paraformaldehyde after 24 h and then stained with 1% crystal violet solution. Migration cell number was measured using microscopy (Zeiss), and the average of five microscopic fields per chamber was calculated.

**Limiting dilution analysis**

GCSC cells were dispersed at defined doses (1 to 20 cells/100 µL) and then seeded onto ultra-low attachment 96-well plates (Corning) in GCSC culture medium ^1^. After 1 week, the numbers of seeded cells and positive tumor spheroids were accessed to determine the “tumor-initiating cell frequency” calculated using the Extreme Limiting Dilution Analysis (ELDA) previously described ^3^.

**LDH releasing assay**

Cell culture supernatants were harvested at indicated time points. Aliquots of supernatants (120 mL/well) were transferred into 96-well plates and treated with LDH detection reagent (60 mL/well; Beyotime) followed by incubation for 30 min at room temperature in the absence of light. The absorbance at 490 nm was measured on a microplate reader (Biotek). The percentage of LDH released was calculated using the equation (LDH_sample_−LDH_background_)/(LDH_maximum_−LDH_background_) × 100%.

**Immunofluorescence (IF) staining**

Cells were seeded at 60-80% confluence onto glass coverslips. Cells were fixed with 4% paraformaldehyde, permeabilized with 0.1% Triton X-100, blocked with 1% bovine serum albumin, and incubated with primary antibodies targeting LGSN (Sigma) and vimentin (Cell Signaling) at 4°C overnight. The cells were incubated with Alexa Fluor dye-conjugated secondary antibodies at room temperature for 1 h, followed by nucleic counterstaining with 4′,6-diamidino-2-phenylindole (DAPI) (Invitrogen). Slides were subsequently mounted with ProLong Diamond Antifade Mountant (Invitrogen). The primary antibodies and second antibodies used are listed in Table S6. Images were obtained using a fluorescence microscope (Biotek) with a 20× objective.

**Immunohistochemical and hematoxylin/****eosin staining analyses of xenografts**

Xenograft specimens freshly excised from mice were immediately incubated in 4% paraformaldehyde, then paraffin-embedded. The samples were sectioned to 7 mm, and the sections underwent xylene deparaffinization, graded ethanol rehydration, and antigen retrieval by microwave heating for 20 min in 1 mM EDTA (pH 8.0). To eliminate the endogenous peroxidase, the sections were incubated with 3% H_2_O_2_ for 20 min. After blocking nonspecific binding with 5% normal goat serum, the tumor specimens were incubated with primary rabbit antibodies to LGSN (1:100, Sigma), Vimentin (1:8000, Proteintech), Caspase1-P20 (1:1500, Proteintech), GSDMD (1:3000, Proteintech), and Ki67 (1:16000, Proteintech) for IHC analysis. Subsequently, the tumor specimens were washed and incubated with biotin-conjugated goat anti-rabbit secondary antibody (1:100, Beyotime) and streptavidin-HRP (1:100, Beyotime), then washed and incubated with DAB (3,3’-diaminobenzidine) (1:2, Beyotime) to activate the staining, and nucleus counterstained with hematoxylin. The TMAs and mouse specimens were processed for hematoxylin and eosin (H&E) staining with similar deparaffinization and rehydration, and then stained with H&E (Servicebio) to observe histopathological changes. The images were scanned and analyzed with the Slide Scan System (SQS1000; TEKSQRAY).

**TUNEL**

A terminal deoxynucleotidyl transferase-mediated dUTP nick-end labeling (TUNEL) assay was applied to examine apoptotic cells in the xenografts from mice. A TUNEL assay kit (Servicebio) was used according to the instructions of the manufacturer. Briefly, paraffin-embedded tissue sections were deparaffinized and rehydrated in xylene and an ethanol gradient at room temperature, then rinsed with distilled water. The antigen retrieval of tissue sections was achieved using proteinase K working solution at 37°C for 25 min, then the sections were rinsed with PBS (pH 7.4). The sections were permeated with 0.1% Triton X-100 at room temperature for 20 min. TUNEL reactions were started using the appropriate amount of terminal deoxynucleotidyl transferase, dUTP, and buffer and incubated at 37°C for 2 h in a humidified chamber. The sections were thoroughly rinsed with PBS (pH 7.4) and counterstained with DAPI at room temperature for 10 min in a dark chamber, followed by anti-fade mounting medium treatment. The staining was examined under ortho-fluorescent microscopy (Nikon Eclipse C1) and photographed using an imaging system (Nikon DS-U3).

**Bioinformatics** **analysis and Code availability**

For GSEA, the whole-genome expression profile of gastric cancer samples from the TCGA database was loaded into GSEA software (<http://software.broadinstitute.org/gsea/index.jsp>) for analysis. Kaplan–Meier plots were accessed online (https://kmplot.com/analysis/). The datasets analyzed for the expression status of LGSN in GC patients can be found in GEO (https://www.ncbi.nlm.nih.gov/geo/). LGSN expression and predictions for the clinical chemotherapeutic or radiotherapy responses of GC patients were all obtained from TCGA. The maximal clique centrality algorithm in the Cytohubba plugin of Cytoscape software was used to predict strong interactors among the differentially expressed genes. R package was downloaded from the website (http://genemed.uchicago.edu/~pgeeleher/pRRophetic, for the IC50 prediction of 5-FU, cisplatin and Metformin) and (https://github.com/maese005/oncoPredict, for the IC50 prediction of L-OHP).

**References**

1 Xiong J, Li Y, Tan X, Fu L. Combined Conditional Knockdown and Adapted Sphere Formation Assay to Study a Stemness-Associated Gene of Patient-derived Gastric Cancer Stem Cells. J Vis Exp. 2020; 2020: 1–7.

2 Lee S, Rodriguez-Villanueva J, McDonnell T. Restrained Terminal Differentiation and Sustained Stemness in Neonatal Skin by Ha-Ras and Bcl-2. Am J Dermatopathol. 2017; 39: 199–203.

3 Hu Y, Smyth GK. ELDA: extreme limiting dilution analysis for comparing depleted and enriched populations in stem cell and other assays. J Immunol Methods. 2009; 347: 70–8.

**Supplementary Legends**

Fig. S1. (Related to Fig. 1) GCSCs stably express CD44 and CD54. A Representative flow cytometric histograms and quantification of CD44- and CD54-positive proportions among two GCSCs cell lines. B Volcano plot for the fold change versus statistical significance in gene expression between GCSC spheroids and differentiated monolayer gastric epithelial-like cells, as determined by RNA-seq.

Fig. S2. (Related to Fig. 1) H&E staining and LGSN score in high- and low-LGSN cases from GC tissue microarray (TMA). A H&E staining of tumor tissue and corresponding adjacent normal tissues from GC TMA; scale bars, 100 μm. B Statistical analysis showed LGSN IHC staining score was associated with the clinical TNM stage of GC. ***P* < 0.01; error bars show mean ± SD.

Fig. S3. (Related to Fig. 1) Correlation between *LGSN* expression and cancer stemness score. A LGSN mRNA expression levels in normal (n = 134) and gastric cancer tissues (n = 134) in the GSE29272 dataset. B–D Kaplan–Meier analysis indicated that high expression of *LGSN* was correlated with poor overall survival (OS), first progression (FP), and post-progression survival (PPS) in GC patients (https://kmplot.com/analysis/). Median *LGSN* expression was used as the cutoff value. *LGSN* Probe set: 220393_at. Statistical significance was determined by log-rank test. E GC patients from TCGA database were dichotomized into two groups with a median value of 0.04695 (TPM) *LGSN* mRNA level: low-LGSN group (shown as blue dots on the left-hand side, n = 181) and high-LGSN group (shown as red dots on the right-hand side, n = 181). F Boxplots of mRNA expression-based stemness index (mRNAsi) analysis in normal and tumor samples in the TCGA cohort. G Correlation between epigenetic regulation-based stemness index (EREG-mRNAsi; left) or DNA methylation-based stemness index (mDNAsi; right) and *LGSN* expression level. Error bars show mean ± SD.

Fig. S4. (Related to Fig. 1) Up-regulation of LGSN in GC cell lines. A Western blot of LGSN protein expression levels in GES-1 and GC cell lines. B Representative morphology images of indicated cells grown as adherently or as spheroids; scale bars, 100 μm. C Immunoblot showing expression levels of LGSN, stemness, EMT and differentiation-related proteins in indicated cells grown as monolayer or spheroids in GCSC completed medium. D Time-course analysis of indicated protein levels in shNTC and sh*LGSN* GCSC1.

**Fig. S5. (Related to Fig. 2) *LGSN* overexpression promotes migration and cisplatin resistance in GES-1 cells. A** Cell migration potential of VEC and *LGSN*-overexpressing GES-1 cells, as detected by Transwell (n=3); scale bars, 200 μm. **B** CCK-8 measurement of IC50 of cisplatin in *LGSN*-overexpressing GES-1 cells (μM) (Log10) (n=3). *****P* < 0.0001; error bars show mean ± SD.

Fig. S6. (Related to Fig. 2) Self-renewal ability of GCSCs. Representative images of GCSC spheroid diameters during time-course analysis; scale bars, 50 μm.

**Fig. S7.** **(Related to Fig. 2) *LGSN* overexpression promotes migration in diffGCSCs. A–B** Cell migration potential of VEC and *LGSN*-overexpressing diffGCSCs, as detected by Transwell (n=3); scale bar, 100 μm. ****P* < 0.001; *****P* < 0.0001; error bars show mean ± SD.

**Fig. S8.** **(Related to Fig. 3) Vimentin interacts with LGSN in GCSCs and vimentin high expression is correlated with a poor prognosis in GC patients.** **A** Co-immunoprecipitation of Flag-tagged LGSN overexpressed in GES-1 and diffGCSC1. LGSN and Flag were immunoprecipitated with anti-Flag antibodies. **B** Coomassie blue staining shows proteins immunoprecipitated by Flag from *LGSN*-overexpressing diffGCSC1 cell lysates. **C** Bubble plot showing GO enrichment analyses of proteins identified by mass spectrometry from the SDS PAGE gel brick of *LGSN*-overexpressing GES-1 cell lysates in Fig. 3A. **D** STRING database prediction of the network of 40 differential protein-protein interactions with LGSN protein (presented as corresponding gene names). **E** Top 10 candidates in the network ranked by Maximal Clique Centrality (MCC) method in Cytoscape (presented as corresponding gene names). **F** Kaplan–Meier analysis indicated that high vimentin expression was correlated with poor overall survival (OS), first progression (FP), and post-progression survival (PPS) of the Kaplan-Meier Plotter database. **G** Vimentin was highly expressed in the majority of gastric carcinoma samples in the GSE29272 dataset. **H** Differentially expressed genes between normal and CG tumor tissues in the GSE29272 dataset. Blue dots (down-regulated genes; n = 6106); red dots (up-regulated genes; n = 5925), and cutoff for Log 2-fold change = 0.138.

**Fig. S9. (Related to Fig. 3) LGSN promotes vimentin-mediated proliferation and migration in diffGCSCs.** **A** CCK-8 detection of cell viability of *LGSN*-overexpressed diffGCSC1 transfected with or without sh*VIM* plasmids (n=3). **B** Rescue experiments to determine cell migration ability of VEC and *LGSN*-overexpressing GES-1 cells transfected with or without sh*VIM* plasmids (n=3); scale bars, 200 μm. **C** Migration potential, as detected by Transwell, of VEC and *LGSN*-overexpressing diffGCSC1 treated with Withaferin A (WFA) (250 nM; 24 h) (n=3); scale bars, 200 μm. **D–E** Western blot indicating the amount of phosphorylated vimentin (p-Vimentin-S56) and total vimentin in *LGSN*-overexpressing (**D**) or *LGSN*-knockdown GCSCs (**E**) after transfection for 48 hours. **P* < 0.05; ***P* < 0.01; *****P* < 0.0001; error bars show mean ± SD.

Fig. S10. (Related to Fig. 4) *LGSN* knockdown inhibits GCSC tumor spheroid formation. Representative images of the morphology of shNTC and sh*LGSN* GCSCs on Days 0, 2, 4, and 6; scale bars, 50 μm.

**Fig. S11. (Related to Fig. 4) *LGSN* knockdown combined with chemotherapy has little effect on mouse body weight.** Body weight was monitored every 3 days for mice bearing subcutaneous xenografts (n=5). ns, not significant; error bars show mean ± SD.

**Fig. S12.** **(Related to Fig. 5) Vimentin rescues *LGSN*-silencing-induced inhibition of proliferation and migration in diffGCSCs.** **A–B** Cell viability of *VIM*-overexpressing diffGCSC1 in the absence or presence of sh*LGSN* plasmids, as detected by CCK-8 (n=3) (**A**) and colony formation assay (n=3) (**B**). **C** Cell migration potential, as detected by Transwell, of sh*LGSN* diffGCSC1 transfected with vimentin overexpression plasmids (n=3); scale bars, 200 μm. **D** Representative images of morphology of GCSCs treated with WFA (500 nM, 24h); scale bars, 50 μm. **P* < 0.05; ***P* < 0.01; ****P* < 0.001; *****P* < 0.0001; error bars show mean ± SD.

Fig. S13. (Related to Fig. 6) Interference of LGSN does not induce cell death in normal cells. A–B Representative images of the morphology (top) and Annexin V/PI (bottom) staining assays of shNTC- or sh*LGSN-* transfected GES-1 (A) and NCM460 (B) cells on Day 3 after shRNA transfection; scale bars, 100 μm. ns, not significant; error bars show mean ± SD.

Fig. S14. (Related to Fig. 6) LGSN promotes chemo-drug resistance in clinical GC patients and GCSCs. A Overall survival (OS) after clinical radiation therapy of GC patients with high and low LGSN expression in the TCGA cohort. B–D Assessment of sensitivity to several chemotherapeutics [5-FU (B), cisplatin (C) and metformin (D)] of high- (n=181) and low- (n=181) LGSN expression groups. E CCK-8 assay of IC50 of cisplatin for shNTC and sh*LGSN* GCSCs (μM) (Log10) (n=3). Error bars show mean ± SD.

**Fig. S15. (Related to Fig. 6) Chemo-drugs accelerates *LGSN*-knockdown-induced GCSC pyroptosis.** Representative light microscope images of shNTC and sh*LGSN* GCSCs treated with 5-FU plus L-OHP or DMSO; scale bars,50 μm.

**Fig. S16.** **(Related to Fig. 7) *LGSN* knockdown combined with chemo-drugs demonstrates minimal toxicity *in vivo*.** **A** Body weight of mice with control and *LGSN-*knockdown xenografts treated with and without 5-FU plus L-OHP was monitored every 3 days (n=5). **B** H&E staining of vital organs from mice, conﬁrming a normal phenotype in the treated mice; scale bars, 50 μm. ns, not significant; error bars show mean ± SD.

**Fig. S17.** **(Related to Fig. 7) Targeting LGSN does not affect the lens in a systemic *in vivo* treatment model.** **A** Representative image of the GSC xenografts removed from nude mice following tail vein injection of LGSN-shRNA AAV9 vector (AAV-sh*LGSN*) or the control shRNA AAV9 vector (AAV-shNTC). Virus was injected once at 2.5 × 10^11^ vg/mouse; scale bar, 1 cm. **B** AAV-sh*LGSN* treatment effectively inhibited tumorigenicity in GCSC xenograft-bearing mice. (n=5). **C** The wet weight of GCSC xenografts removed from AAV-sh*LGSN* treatment group or AAV-shNTC treatment group was measured at Day 57 post-injection (n=5). **D** Protein expression of the LGSN was evaluated by WB in AAV-sh*LGSN*-treated GCSC xenografts as compared to their controls. **E** Representative images of green fluorescence assay, H&E staining and IHC staining of LGSN showing virus infection in tumor cells of GCSC xenografts (top) but not in eyes of mice (bottom); scale bars, 200 μm (GFP), 50 μm (H&E), 500 μm (IHC-top) or 20 μm (IHC-bottom). Arrows indicate LGSN-positive lens epithelial cells. **F** Representative images of macroscopic views (top) and H&E staining (bottom) showing the normal phenotype in eyes after AAV-sh*LGSN* or AAV-shNTC treatment; scale bars, 500 μm (black) or 50 μm (white). ****P* < 0.001; *****P* < 0.0001; error bars show mean ± SD.

**Fig. S18.** **(Related to Fig. 7) AAV-mediated *in vivo* knockdown of *LGSN* demonstrates minimal toxicity in mouse.** **A** Body weight of mice with control and AAV-sh*LGSN*-treated GCSC xenografts was monitored every 5 days (n=5). **B** Green fluorescence assay (left) and H&E staining (right) of vital organs from mice, conﬁrming a normal phenotype in the treated mice; scale bars, 200 μm (white) or 50 μm (black). ns, not significant; error bars show mean ± SD.
