# Supplementary material for: Targeting LGSN restores sensitivity to chemotherapy in gastric cancer stem cells by triggering pyroptosis
Source: Cell Death Dis. 2023 Aug 23;14(8):545. doi: 10.1038/s41419-023-06081-8 (PMC10447538; doi:10.1038/s41419-023-06081-8)
Supplement: Supplementary file 2 — Li et al_Supplementary Figures [file 41419_2023_6081_MOESM2_ESM.pdf]

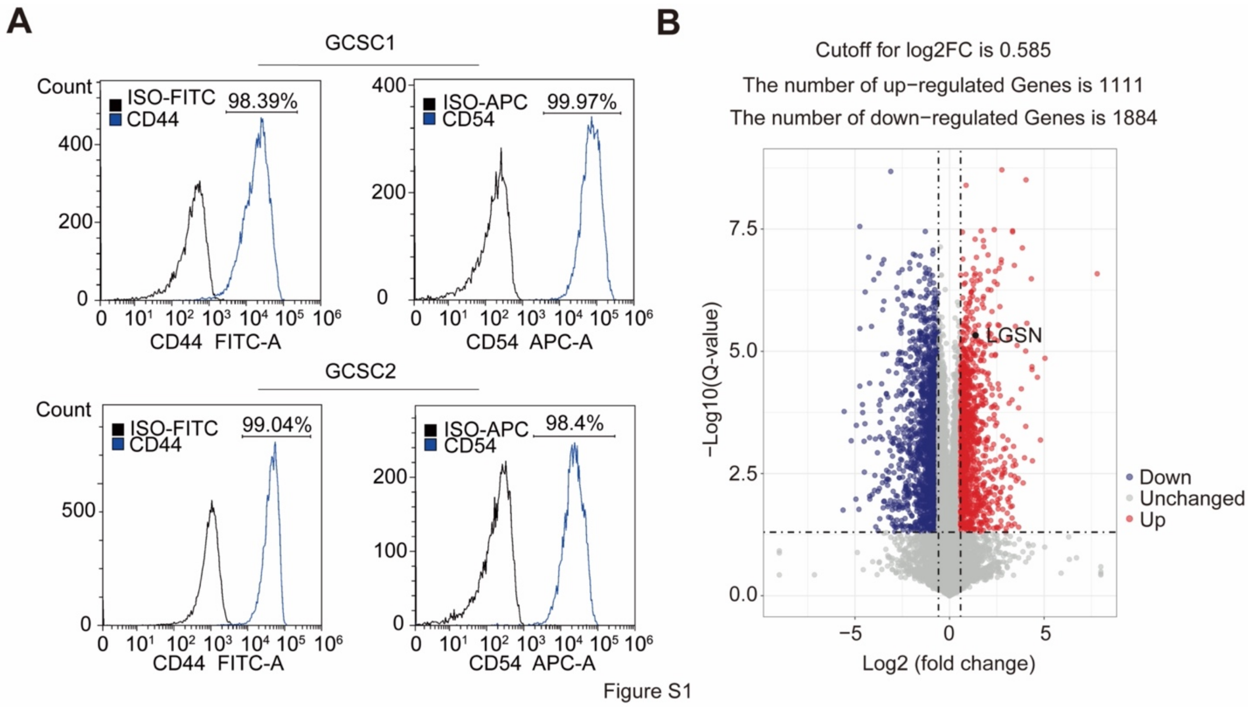
A
GCSC1
Count
ISO-FITC
CD44
98.39%
CD44 FITC-A
ISO-APC
CD54
99.97%
CD54 APC-A
GCSC2
Count
ISO-FITC
CD44
99.04%
CD44 FITC-A
ISO-APC
CD54
98.4%
CD54 APC-A
B
Cutoff for log2FC is 0.585
The number of up−regulated Genes is 1111
The number of down−regulated Genes is 1884
LGSN
−Log10(Q-value)
Log2 (fold change)
Down
Unchanged
Up

Figure S1

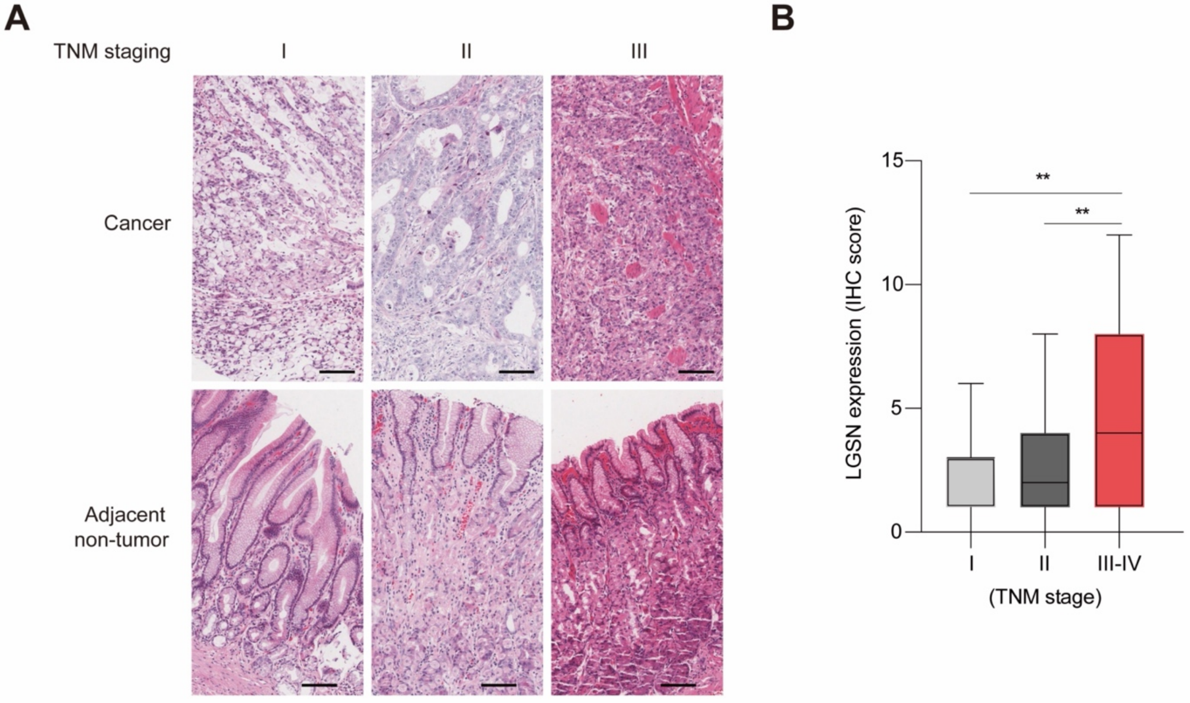
A
TNM staging
I
II
III
Cancer
Adjacent non-tumor
B
LGSN expression (IHC score)
15
10
5
0
**
**
I
II
III-IV
(TNM stage)

Figure S2

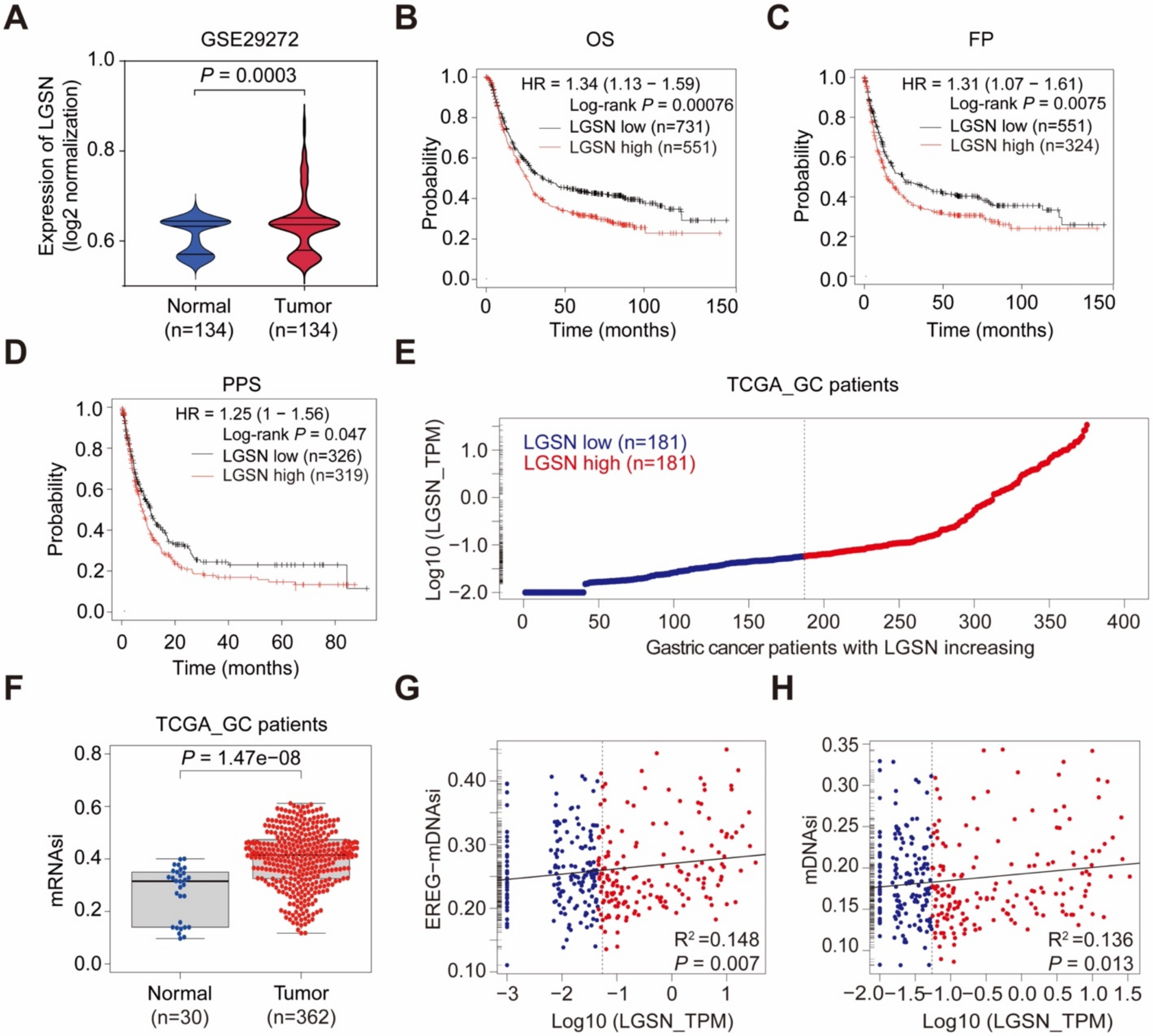
A
GSE29272
P = 0.0003
Expression of LGSN (log2 normalization)
Normal (n=134)
Tumor (n=134)
B
OS
HR = 1.34 (1.13 − 1.59)
Log-rank P = 0.00076
LGSN low (n=731)
LGSN high (n=551)
Probability
Time (months)
C
FP
HR = 1.31 (1.07 − 1.61)
Log-rank P = 0.0075
LGSN low (n=551)
LGSN high (n=324)
Probability
Time (months)
D
PPS
HR = 1.25 (1 − 1.56)
Log-rank P = 0.047
LGSN low (n=326)
LGSN high (n=319)
Probability
Time (months)
E
TCGA_GC patients
LGSN low (n=181)
LGSN high (n=181)
Log10 (LGSN_TPM)
Gastric cancer patients with LGSN increasing
F
TCGA_GC patients
P = 1.47e−08
mRNAsi
Normal (n=30)
Tumor (n=362)
G
EREG−mDNAsi
R2 =0.148
P = 0.007
Log10 (LGSN_TPM)
H
mDNAsi
R2 =0.136
P = 0.013
Log10 (LGSN_TPM)

Figure S3

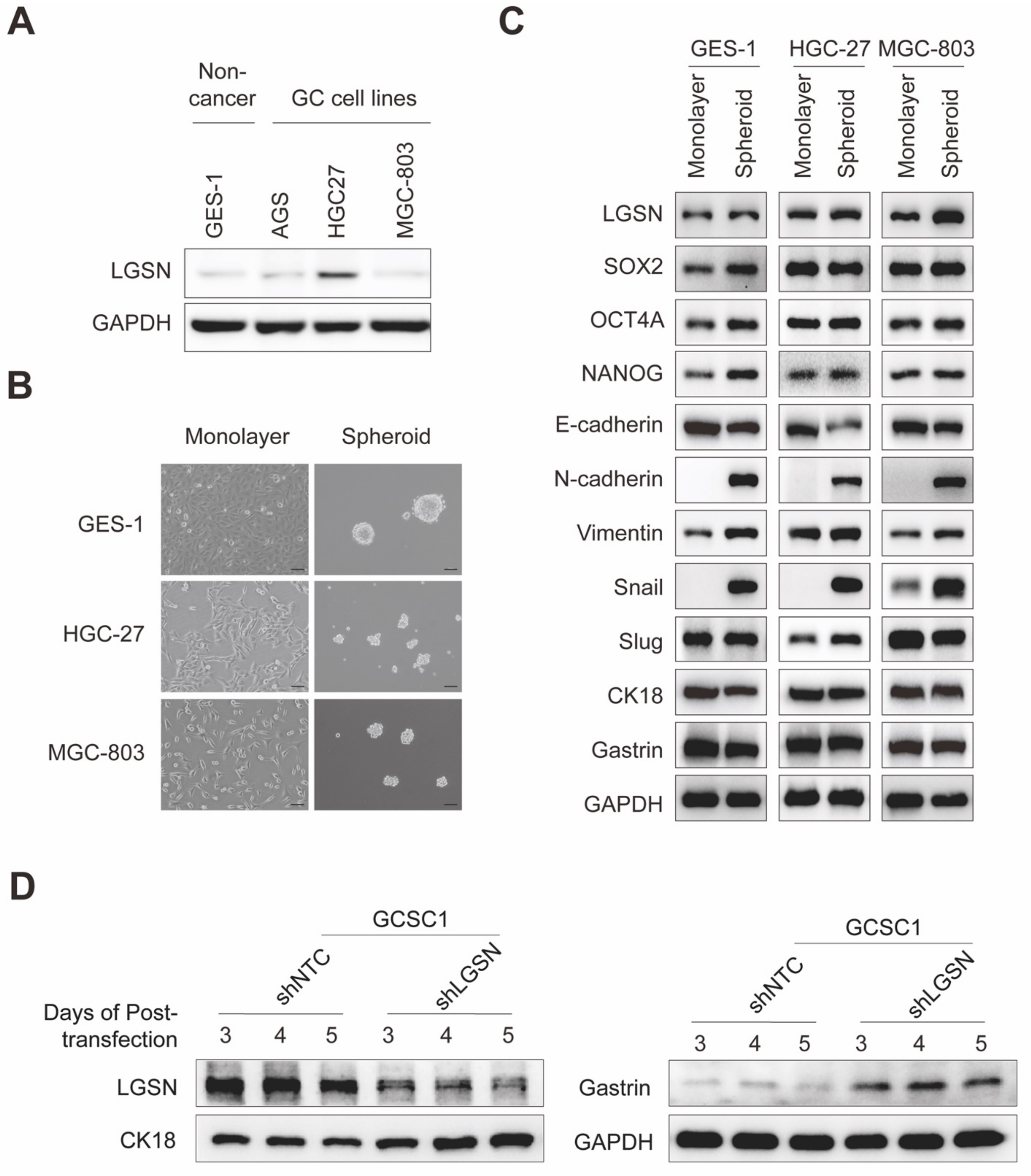
A
Non-cancer
GC cell lines
GES-1
AGS
HGC27
MGC-803
LGSN
GAPDH
B
Monolayer
Spheroid
GES-1
HGC-27
MGC-803
C
GES-1
HGC-27
MGC-803
Monolayer
Spheroid
LGSN
SOX2
OCT4A
NANOG
E-cadherin
N-cadherin
Vimentin
Snail
Slug
CK18
Gastrin
GAPDH
D
GCSC1
shNTC
shLGSN
Days of Post-transfection
3 4 5 3 4 5
LGSN
CK18
Gastrin
GAPDH

Figure S4

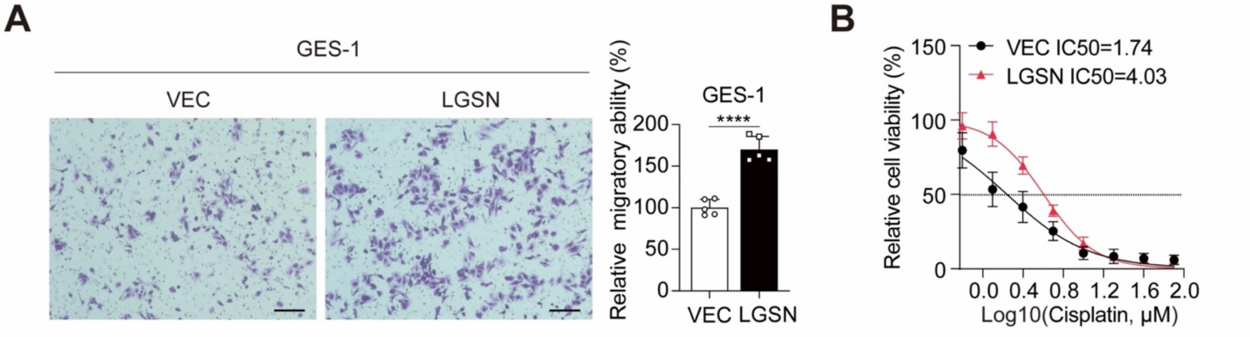
A
GES-1
VEC
LGSN
Relative migratory ability (%)
GES-1
****
0
50
100
150
200
VEC
LGSN
B
Relative cell viability (%)
VEC IC50=1.74
LGSN IC50=4.03
0
50
100
150
0.0
0.4
0.8
1.2
1.6
2.0
Log10(Cisplatin, μM)

Figure S5

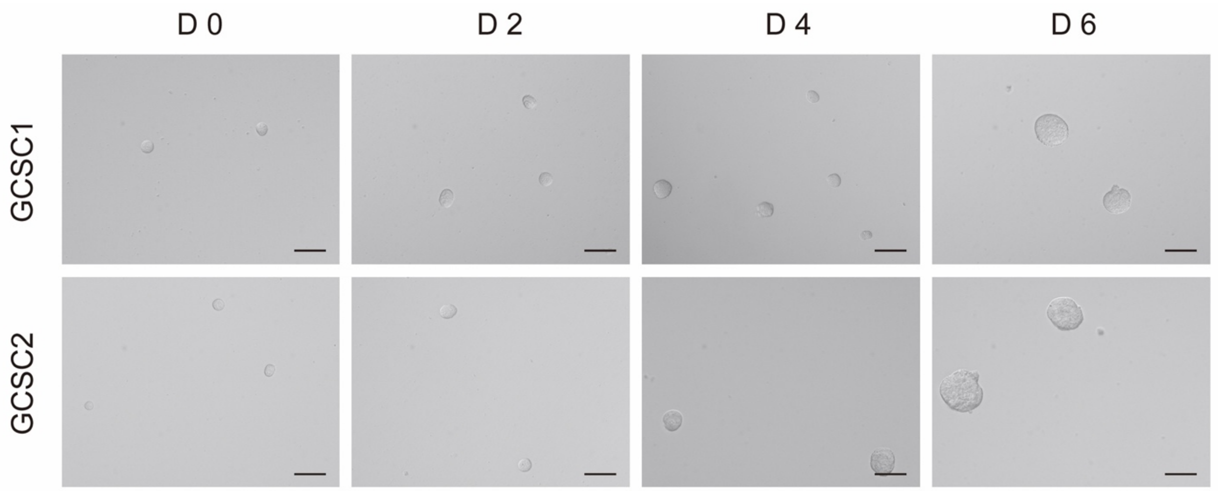

Figure S6

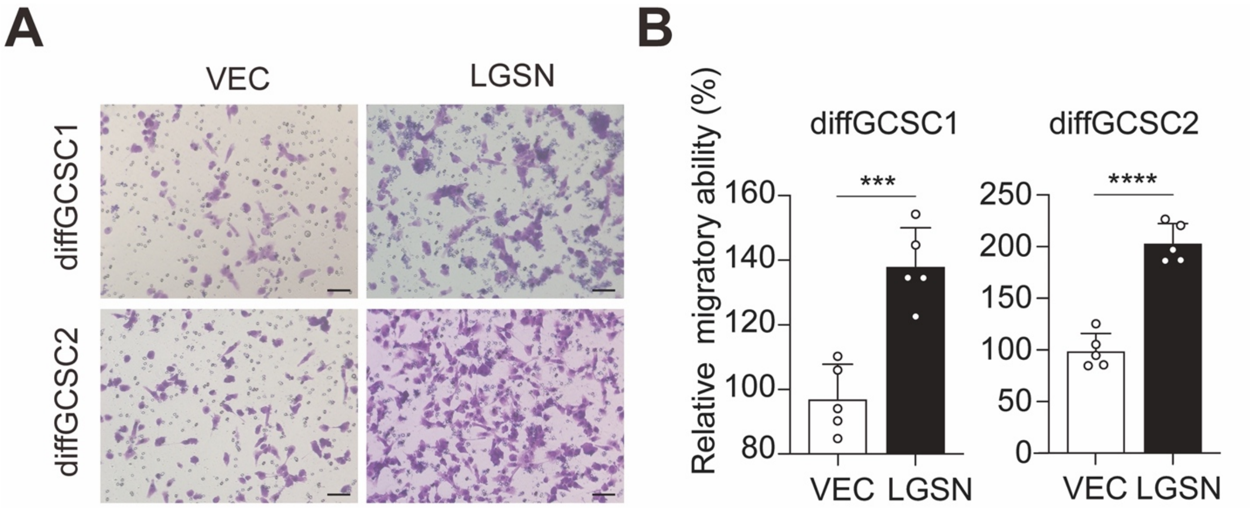
A
VEC
LGSN
diffGCSC1
diffGCSC2
B
Relative migratory ability (%)
diffGCSC1
***
160
140
120
100
80
VEC
LGSN
diffGCSC2
****
250
200
150
100
50
0
VEC
LGSN

Figure S7

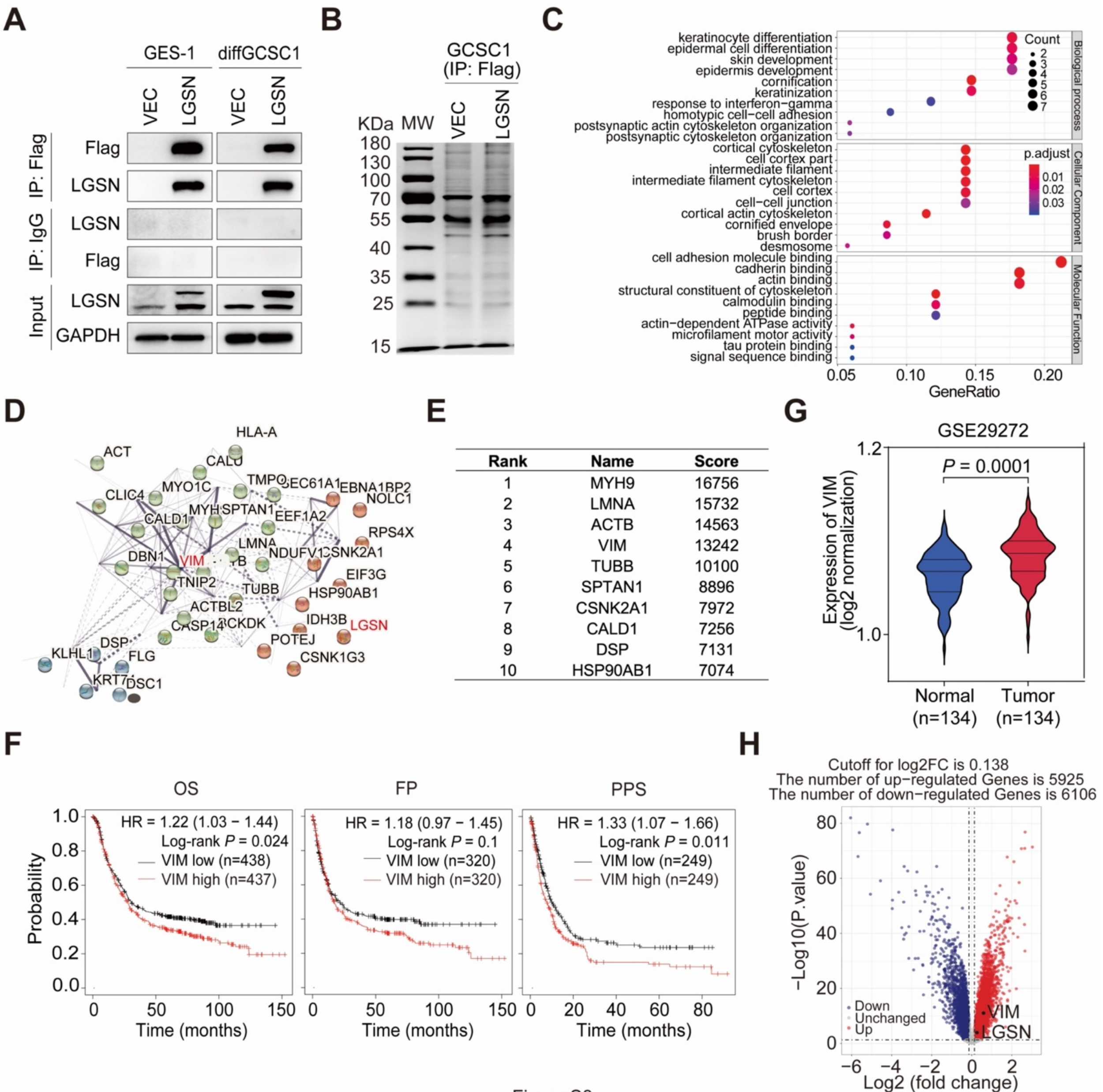
A
GES-1
diffGCSC1
VEC
LGSN
IP: Flag
IP: IgG
Input
Flag
LGSN
GAPDH
B
GCSC1
(IP: Flag)
KDa
MW
180
130
100
70
55
40
35
25
15
C
keratinocyte differentiation
epidermal cell differentiation
skin development
epidermis development
cornification
keratinization
response to interferon−gamma
homotypic cell−cell adhesion
postsynaptic actin cytoskeleton organization
postsynaptic cytoskeleton organization
cortical cytoskeleton
cell cortex part
intermediate filament
intermediate filament cytoskeleton
cell cortex
cell−cell junction
cortical actin cytoskeleton
cornified envelope
brush border
desmosome
cell adhesion molecule binding
cadherin binding
actin binding
structural constituent of cytoskeleton
calmodulin binding
peptide binding
actin−dependent ATPase activity
microfilament motor activity
tau protein binding
signal sequence binding
Biological process
Cellular Component
Molecular Function
Count
p.adjust
GeneRatio
D
VIM
LGSN
E
Rank Name Score
1 MYH9 16756
2 LMNA 15732
3 ACTB 14563
4 VIM 13242
5 TUBB 10100
6 SPTAN1 8896
7 CSNK2A1 7972
8 CALD1 7256
9 DSP 7131
10 HSP90AB1 7074
G
GSE29272
P = 0.0001
Expression of VIM (log2 normalization)
Normal (n=134)
Tumor (n=134)
F
OS
HR = 1.22 (1.03 − 1.44)
Log-rank P = 0.024
VIM low (n=438)
VIM high (n=437)
FP
HR = 1.18 (0.97 − 1.45)
Log-rank P = 0.1
VIM low (n=320)
VIM high (n=320)
PPS
HR = 1.33 (1.07 − 1.66)
Log-rank P = 0.011
VIM low (n=249)
VIM high (n=249)
Probability
Time (months)
H
Cutoff for log2FC is 0.138
The number of up−regulated Genes is 5925
The number of down−regulated Genes is 6106
−Log10(P.value)
Log2 (fold change)
Down
Unchanged
Up
VIM
LGSN

Figure S8

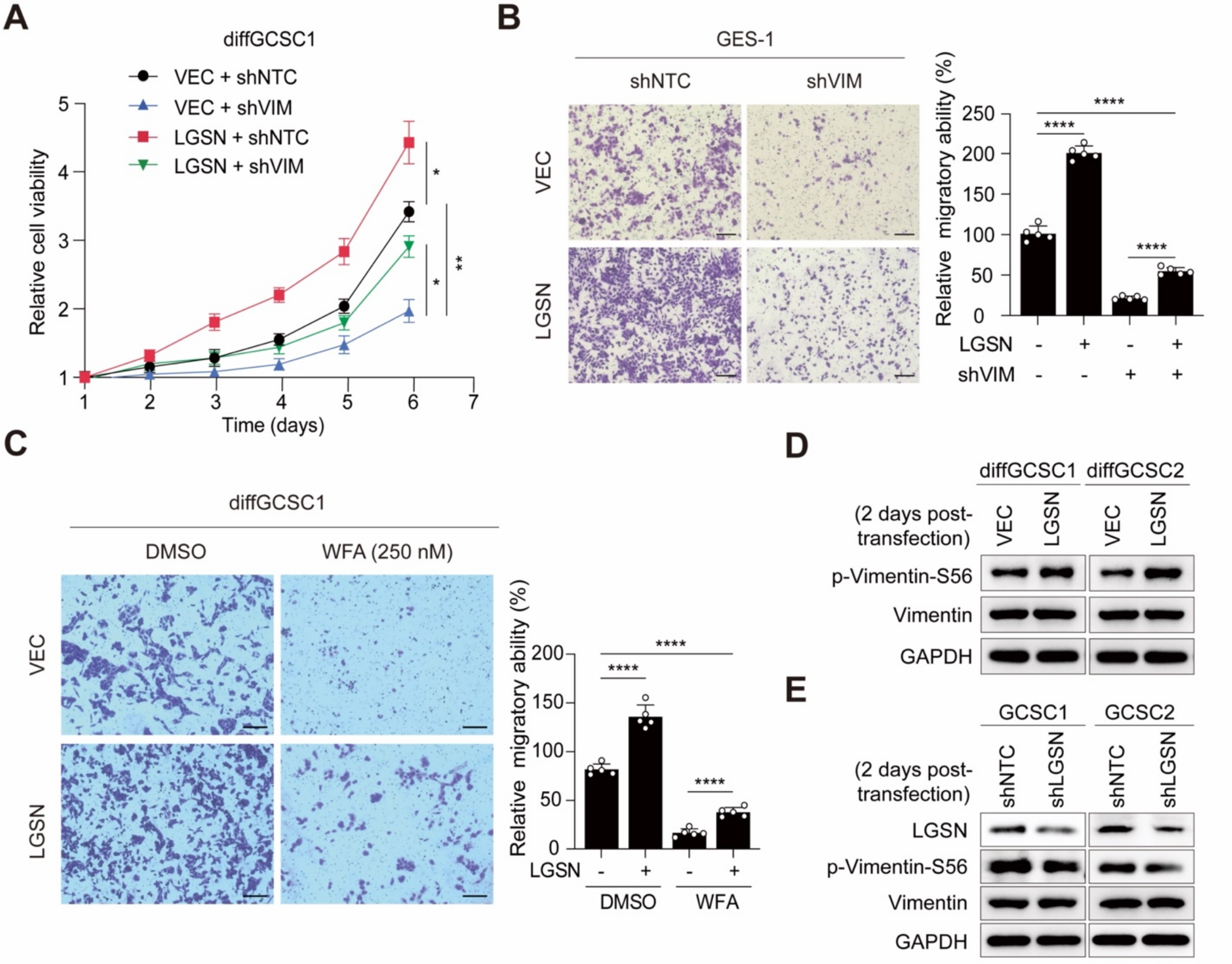
A
diffGCSC1
VEC + shNTC
VEC + shVIM
LGSN + shNTC
LGSN + shVIM
Relative cell viability
Time (days)
B
GES-1
shNTC
shVIM
VEC
LGSN
Relative migratory ability (%)
LGSN
shVIM
C
diffGCSC1
DMSO
WFA (250 nM)
VEC
LGSN
Relative migratory ability (%)
LGSN
DMSO
WFA
D
diffGCSC1
diffGCSC2
(2 days post-transfection)
VEC
LGSN
p-Vimentin-S56
Vimentin
GAPDH
E
GCSC1
GCSC2
(2 days post-transfection)
shNTC
shLGSN
LGSN
p-Vimentin-S56
Vimentin
GAPDH

Figure S9

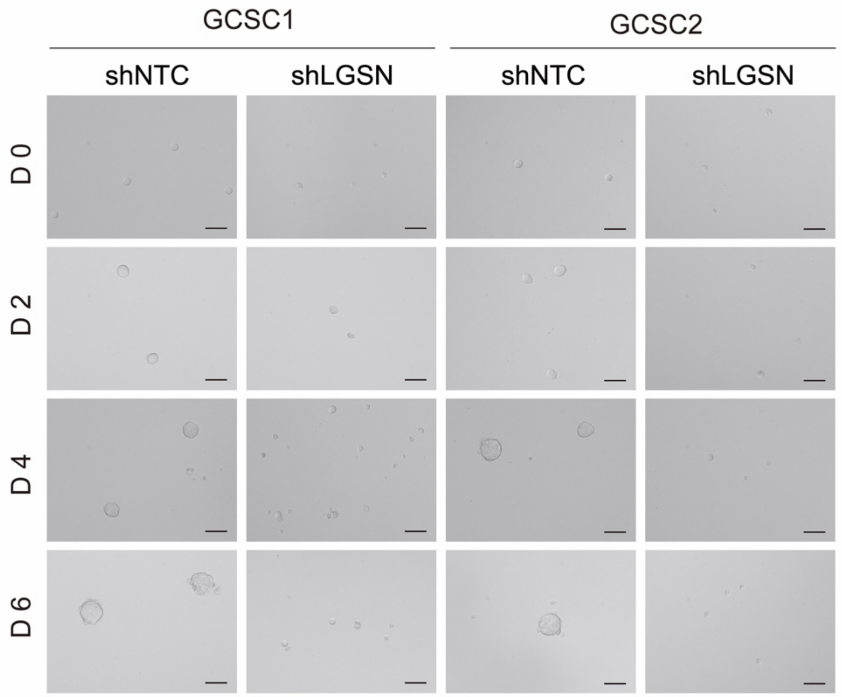

Figure S10

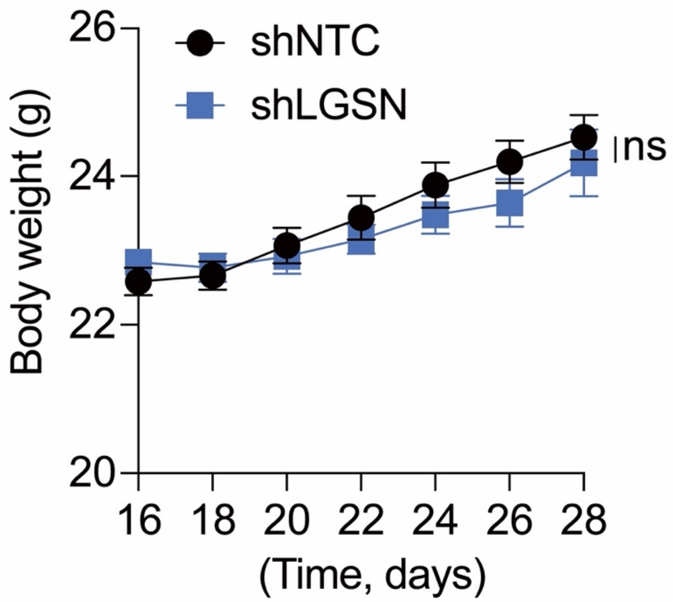
26
24
22
20
Body weight (g)
shNTC
shLGSN
ns
16
18
20
22
24
26
28
(Time, days)

Figure S11

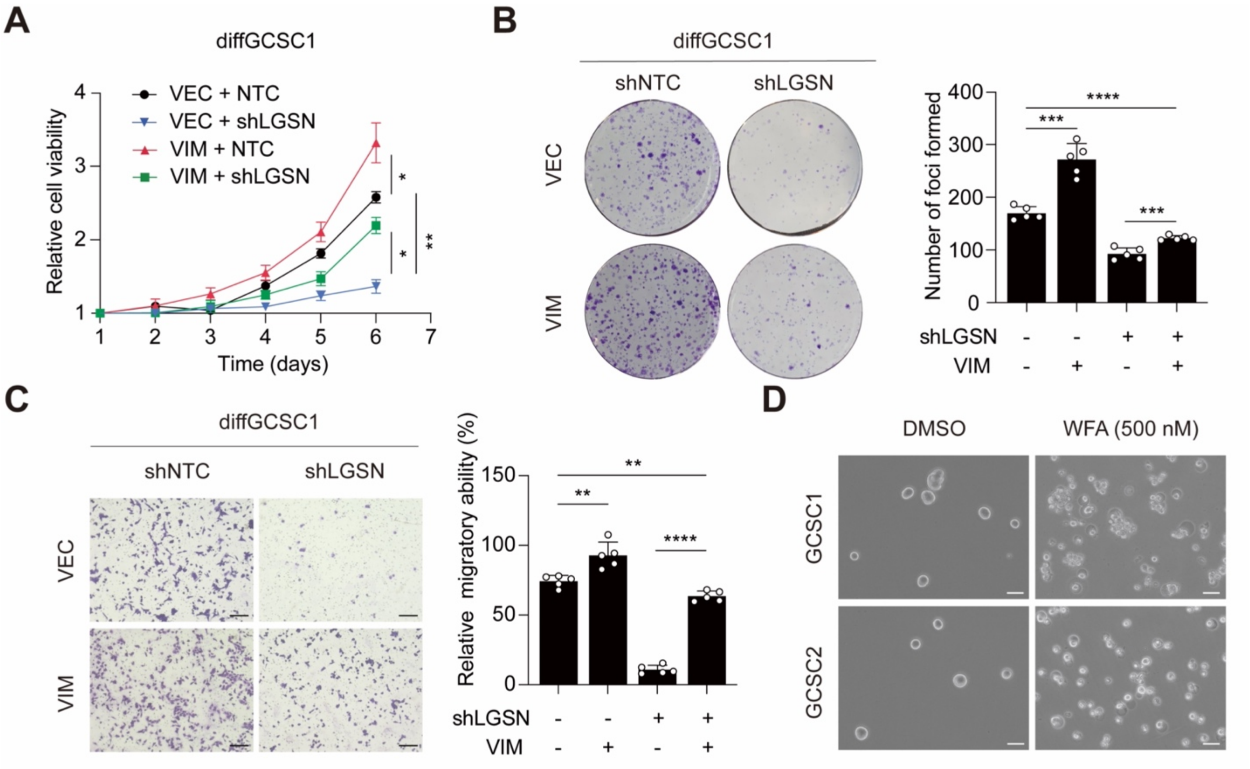
A
diffGCSC1
VEC + NTC
VEC + shLGSN
VIM + NTC
VIM + shLGSN
Relative cell viability
Time (days)
B
diffGCSC1
shNTC
shLGSN
VEC
VIM
Number of foci formed
shLGSN
VIM
C
diffGCSC1
shNTC
shLGSN
VEC
VIM
Relative migratory ability (%)
shLGSN
VIM
D
DMSO
WFA (500 nM)
GCSC1
GCSC2

Figure S12

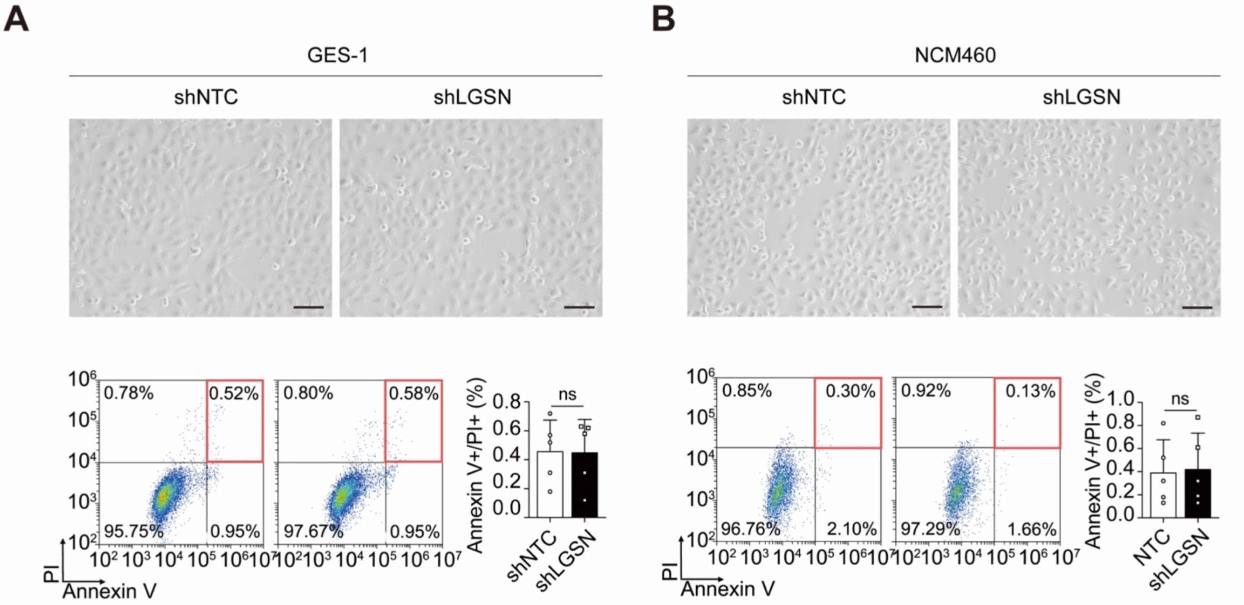
A
GES-1
shNTC
shLGSN
0.78%
0.52%
95.75%
0.95%
0.80%
0.58%
97.67%
0.95%
PI
Annexin V
Annexin V+/PI+ (%)
ns
shNTC
shLGSN
B
NCM460
shNTC
shLGSN
0.85%
0.30%
96.76%
2.10%
0.92%
0.13%
97.29%
1.66%
PI
Annexin V
Annexin V+/PI+ (%)
ns
NTC
shLGSN

Figure S13

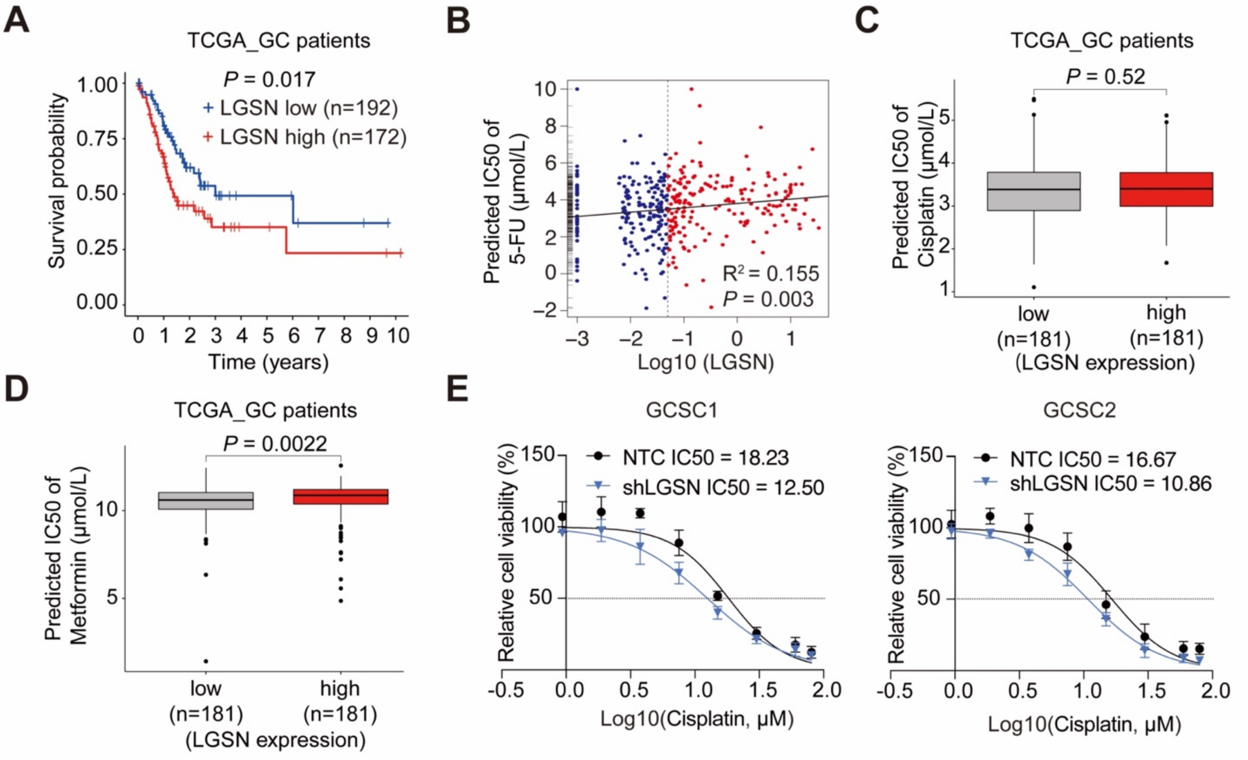

A
TCGA_GC patients
P = 0.017
LGSN low (n=192)
LGSN high (n=172)
Survival probability
Time (years)
B
Predicted IC50 of 5-FU (μmol/L)
R² = 0.155
P = 0.003
Log10 (LGSN)
C
TCGA_GC patients
P = 0.52
Predicted IC50 of Cisplatin (μmol/L)
low (n=181)
high (n=181)
(LGSN expression)
D
TCGA_GC patients
P = 0.0022
Predicted IC50 of Metformin (μmol/L)
low (n=181)
high (n=181)
(LGSN expression)
E
GCSC1
NTC IC50 = 18.23
shLGSN IC50 = 12.50
Relative cell viability (%)
Log10(Cisplatin, μM)
GCSC2
NTC IC50 = 16.67
shLGSN IC50 = 10.86
Relative cell viability (%)
Log10(Cisplatin, μM)

Figure S14

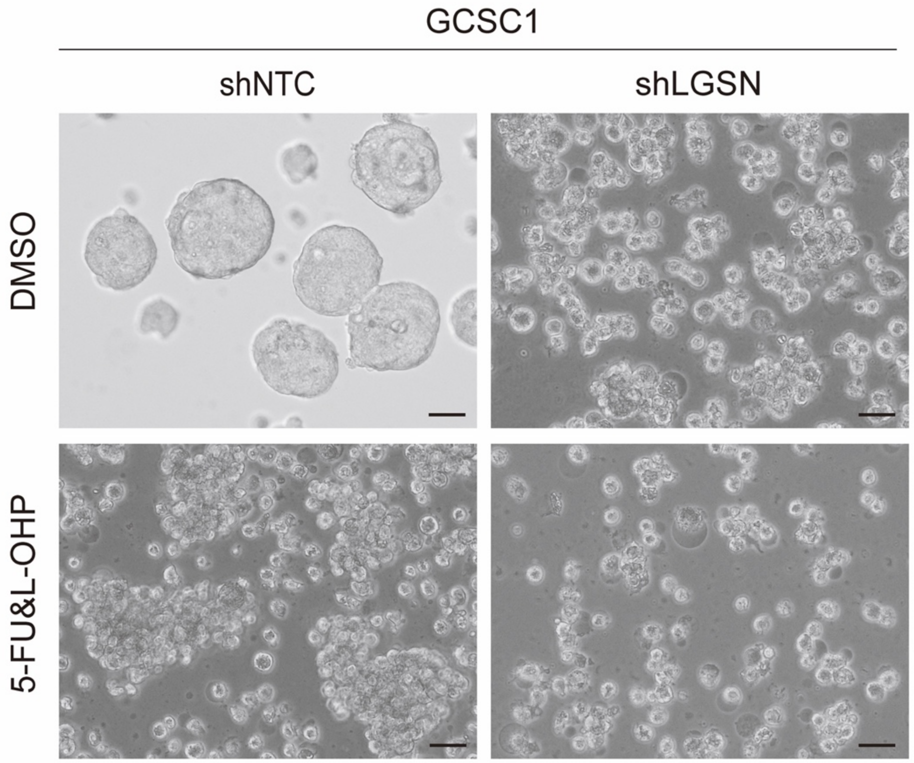

Figure S15

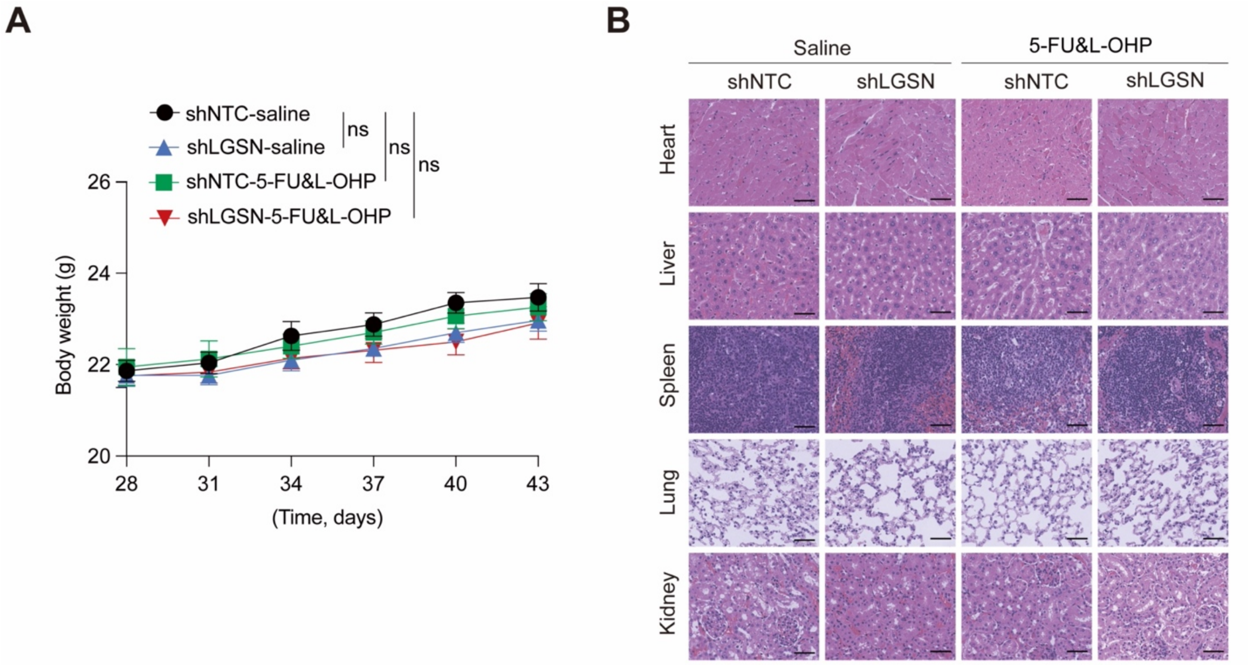
A
shNTC-saline
shLGSN-saline
shNTC-5-FU&L-OHP
shLGSN-5-FU&L-OHP
ns
ns
ns
Body weight (g)
26
24
22
20
28
31
34
37
40
43
(Time, days)
B
Saline
5-FU&L-OHP
shNTC
shLGSN
shNTC
shLGSN
Heart
Liver
Spleen
Lung
Kidney

Figure S16

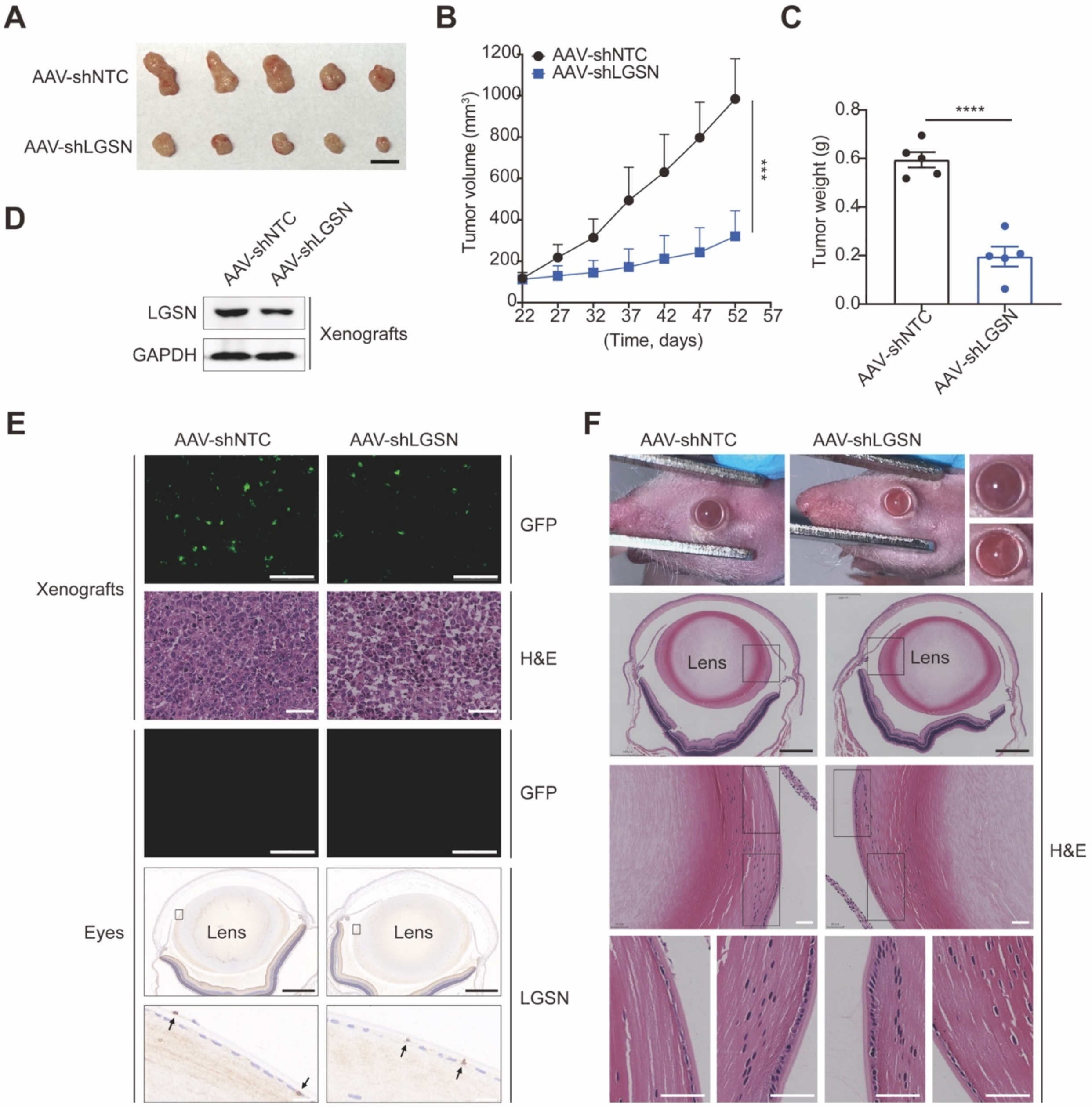
A
AAV-shNTC
AAV-shLGSN
B
AAV-shNTC
AAV-shLGSN
Tumor volume (mm3)
1200
1000
800
600
400
200
0
22 27 32 37 42 47 52 57
(Time, days)
***
C
Tumor weight (g)
0.8
0.6
0.4
0.2
0.0
****
AAV-shNTC
AAV-shLGSN
D
AAV-shNTC
AAV-shLGSN
LGSN
GAPDH
Xenografts
E
AAV-shNTC
AAV-shLGSN
Xenografts
GFP
H&E
GFP
Eyes
Lens
Lens
LGSN
F
AAV-shNTC
AAV-shLGSN
Lens
Lens
H&E

Figure S17

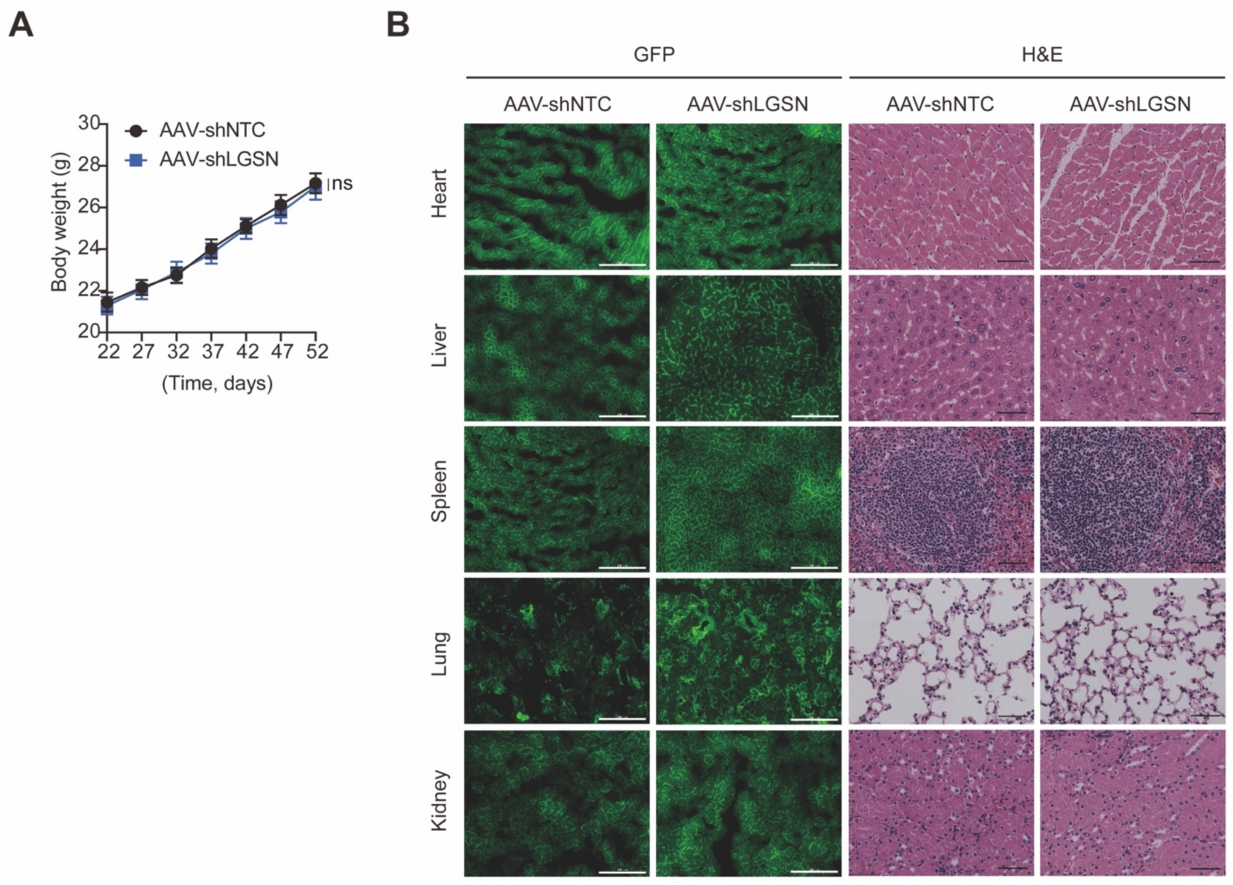
A
AAV-shNTC
AAV-shLGSN
ns
Body weight (g)
30
28
26
24
22
20
22
27
32
37
42
47
52
(Time, days)
B
GFP
H&E
AAV-shNTC
AAV-shLGSN
AAV-shNTC
AAV-shLGSN
Heart
Liver
Spleen
Lung
Kidney

Figure S18
