# Supplementary material for: Targeting LGSN restores sensitivity to chemotherapy in gastric cancer stem cells by triggering pyroptosis
Source: Cell Death Dis. 2023 Aug 23;14(8):545. doi: 10.1038/s41419-023-06081-8 (PMC10447538; doi:10.1038/s41419-023-06081-8)
Supplement: Supplementary file 3 — Li et al_Author Contributions Statement [file 41419_2023_6081_MOESM3_ESM.docx]

**Author Contributions**

Conceptualization, Y.-T.L., X.-Y.T., J.-X.X. and L.F.; methodology, Y.-T.L., X.-Y.T., and L.F.; investigation, Y.-T.L., X.-Y.T., L.-X.M., H.-H.L., and L.F.; writing – original draft, Y.-T.L., and X.-Y.T.; writing – review & editing, Y.-T.L., X.-Y.T., and L.F.; resources, Y.-T.L., X.-Y.T., L.-X.M., H.-H.L., S.-H.Z., L.-N.H., and J.-X.X.; formal analysis, Y.-T.L., X.-Y.T., and H.-H.L.; data curation, Y.-T.L., X.-Y.T., L.-X.M., H.-H.L. and C.-M.Z.; visualization, Y.-T.L., X.-Y.T., H.-H.L., and S.-H.Z.; funding acquisition, J.-X.X. and L.F.
