# Supplementary material for: Targeting LGSN restores sensitivity to chemotherapy in gastric cancer stem cells by triggering pyroptosis
Source: Cell Death Dis. 2023 Aug 23;14(8):545. doi: 10.1038/s41419-023-06081-8 (PMC10447538; doi:10.1038/s41419-023-06081-8)

Figure 1E

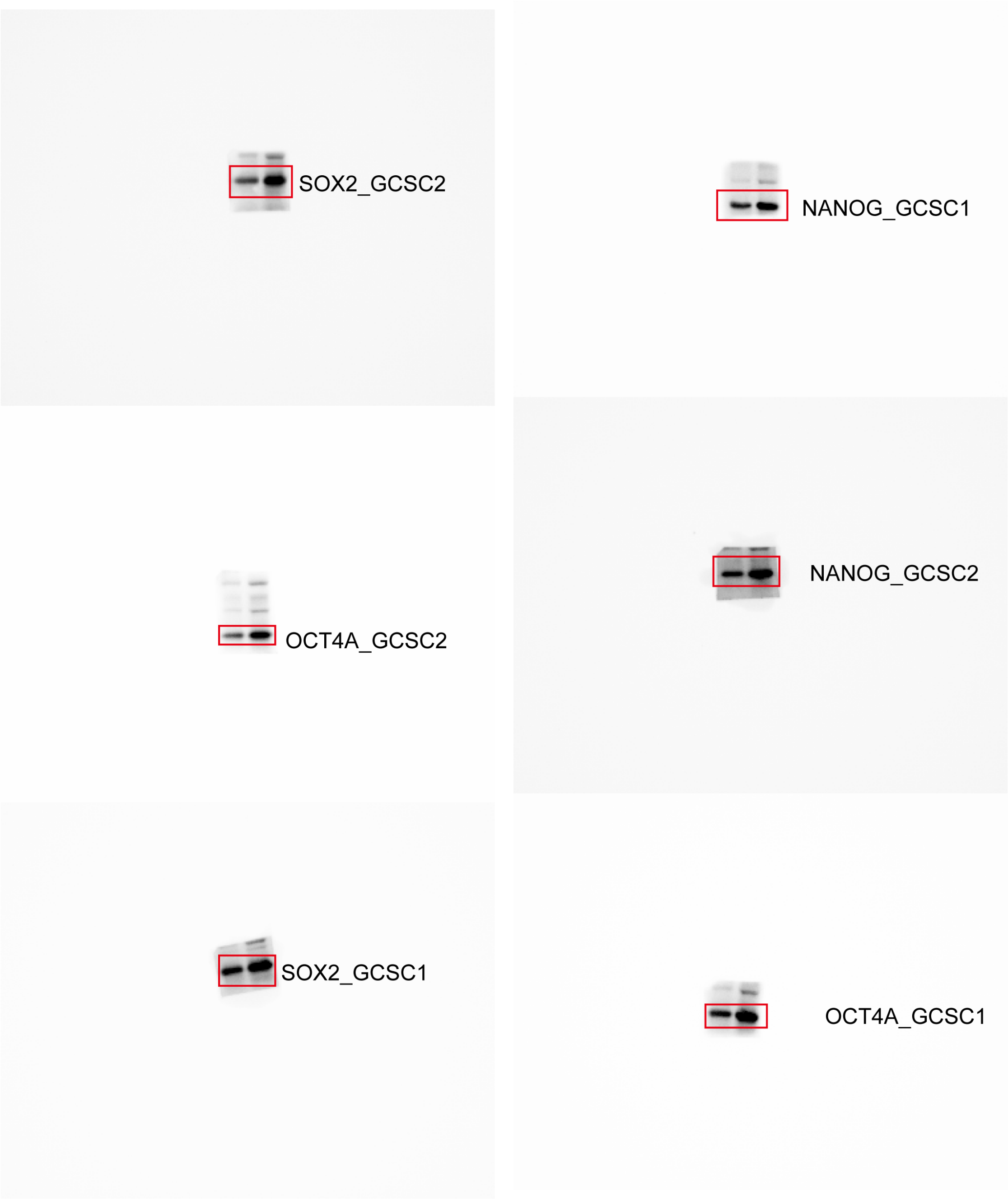

SOX2_GCSC2
NANOG_GCSC1
OCT4A_GCSC2
NANOG_GCSC2
SOX2_GCSC1
OCT4A_GCSC1

Figure 1E

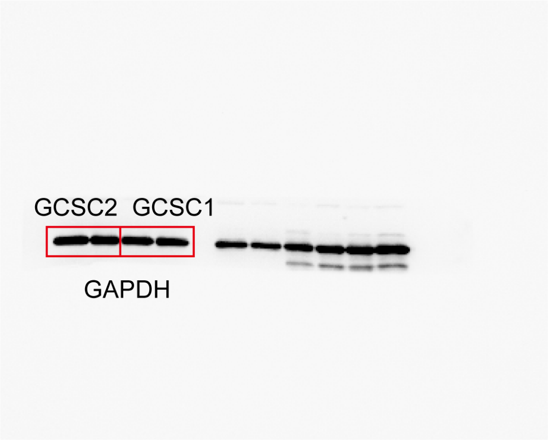

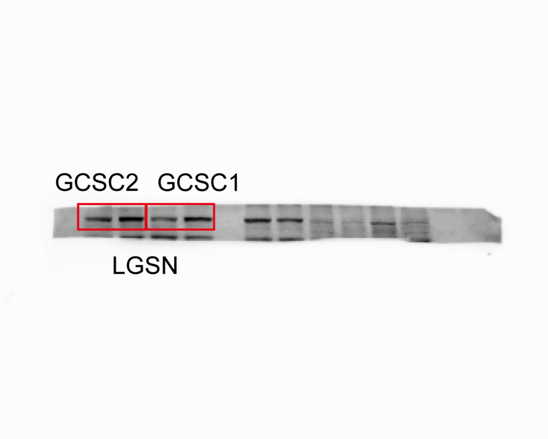

Figure 2A

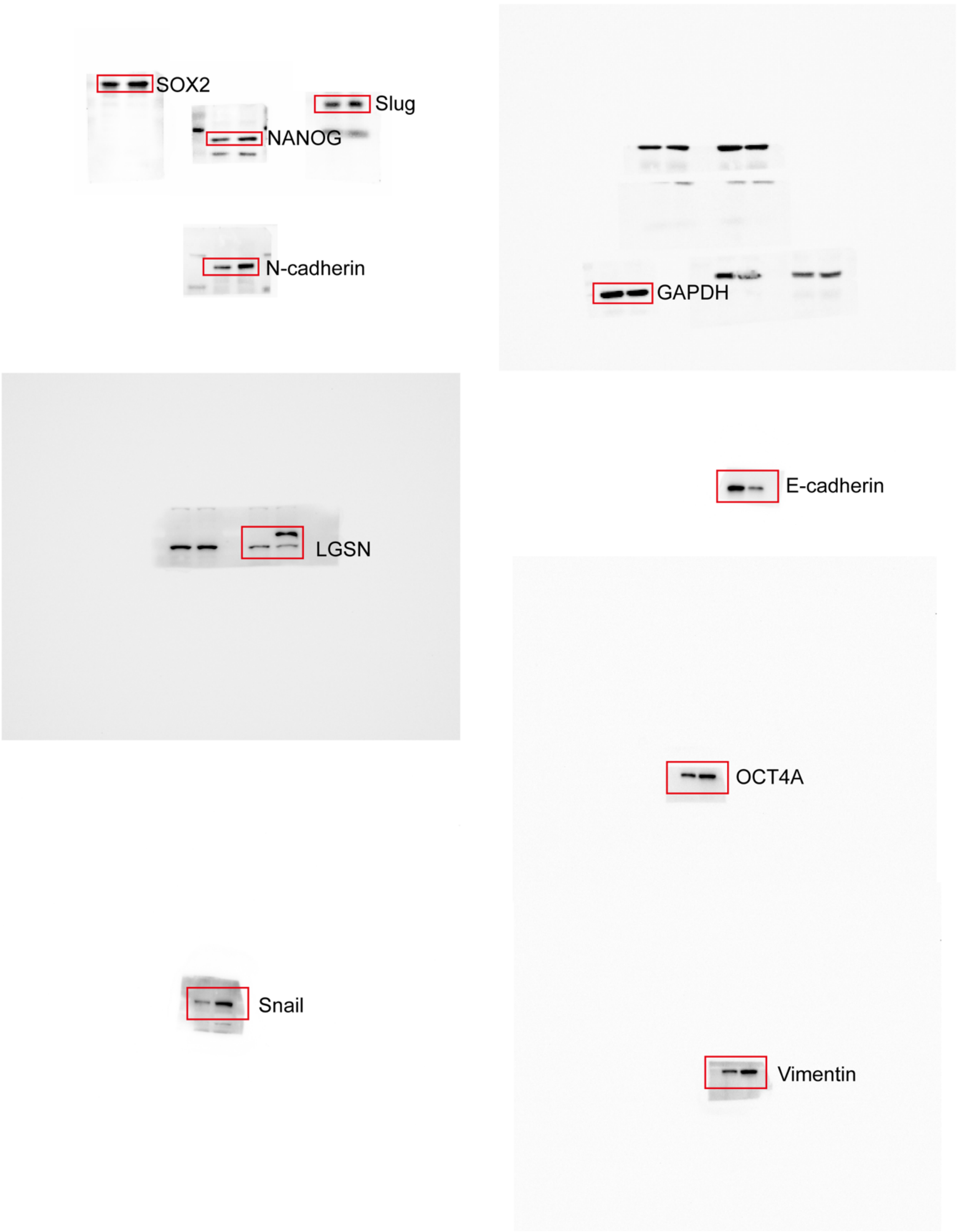
SOX2
Slug
NANOG
N-cadherin
GAPDH
E-cadherin
LGSN
OCT4A
Snail
Vimentin

Figure 2G

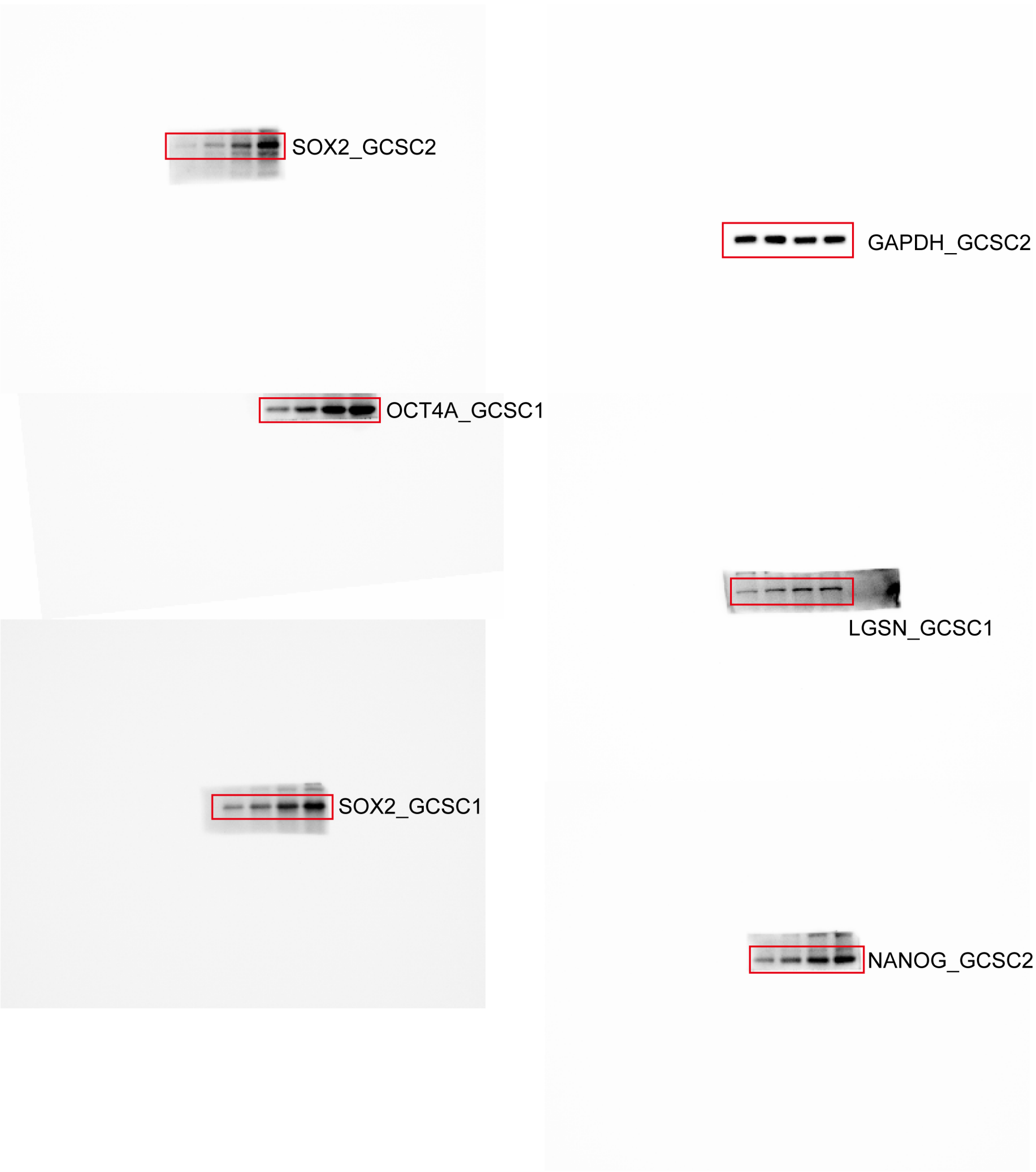
SOX2_GCSC2
GAPDH_GCSC2
OCT4A_GCSC1
LGSN_GCSC1
SOX2_GCSC1
NANOG_GCSC2

Figure 2G

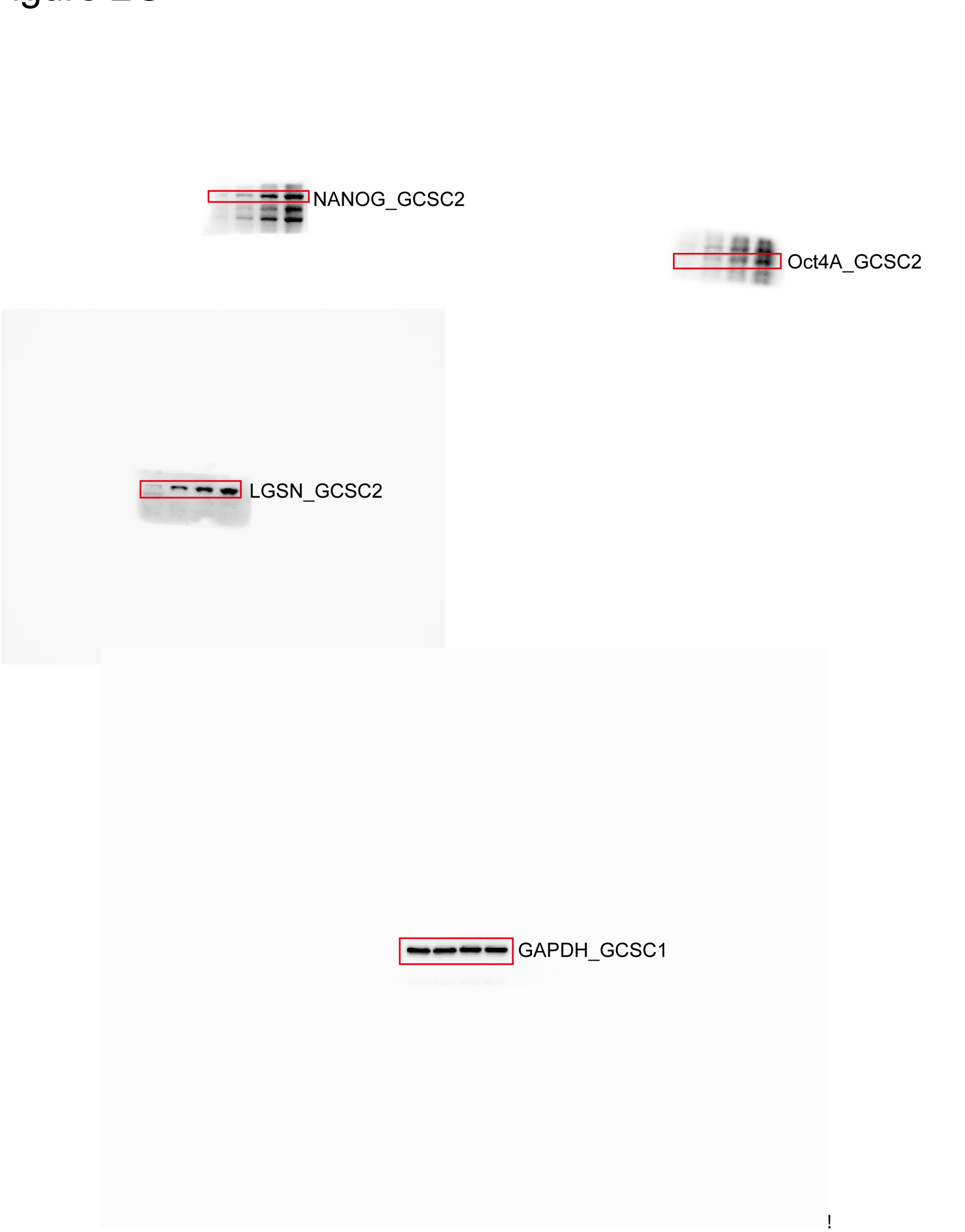

Figure 2I

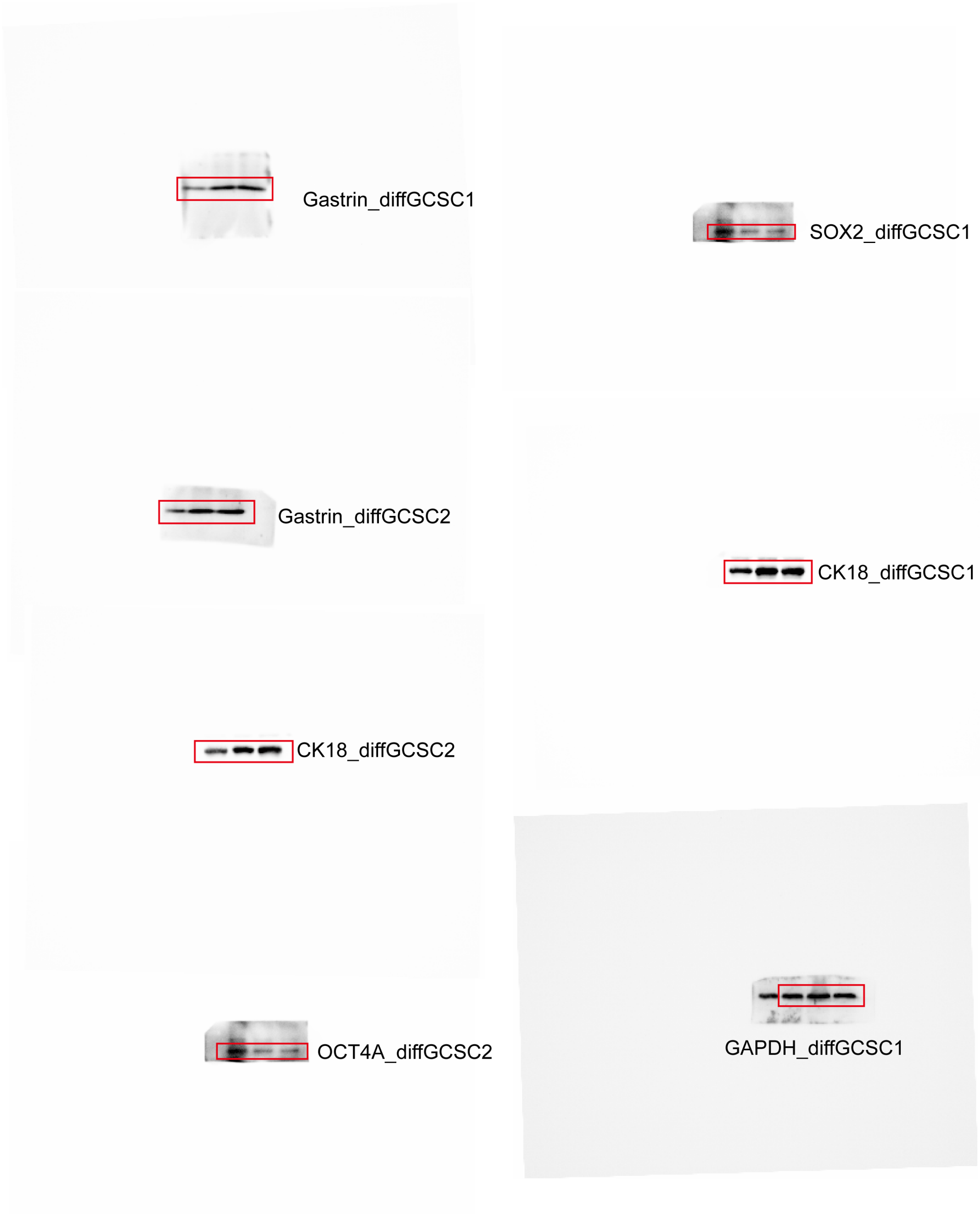

Gastrin_diffGCSC1
SOX2_diffGCSC1
Gastrin_diffGCSC2
CK18_diffGCSC1
CK18_diffGCSC2
OCT4A_diffGCSC2
GAPDH_diffGCSC1

Figure 2J

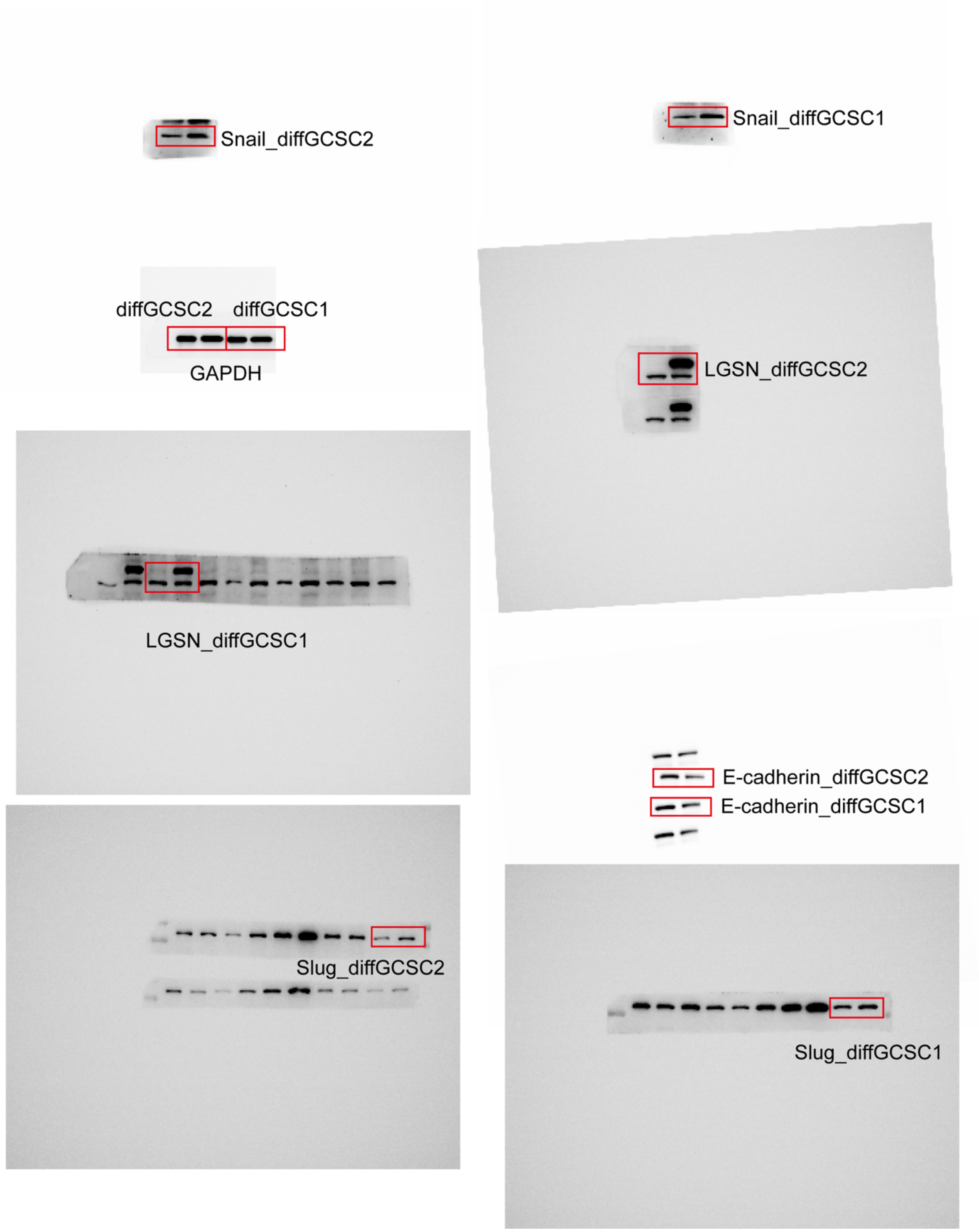
Snail_diffGCSC2
Snail_diffGCSC1
diffGCSC2
diffGCSC1
GAPDH
LGSN_diffGCSC2
LGSN_diffGCSC1
E-cadherin_diffGCSC2
E-cadherin_diffGCSC1
Slug_diffGCSC2
Slug_diffGCSC1

Figure 2J

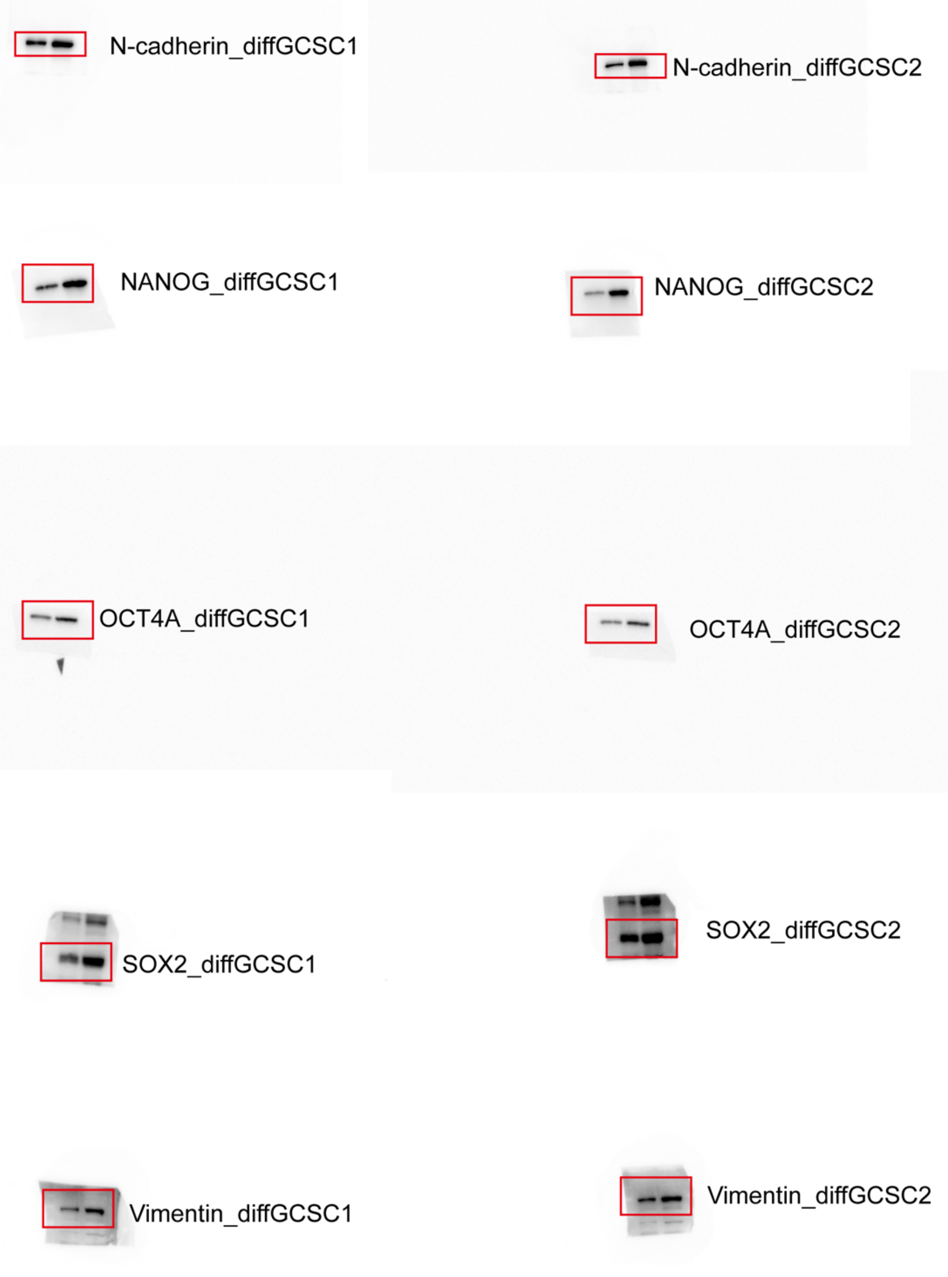
N-cadherin_diffGCSC1
N-cadherin_diffGCSC2
NANOG_diffGCSC1
NANOG_diffGCSC2
OCT4A_diffGCSC1
OCT4A_diffGCSC2
SOX2_diffGCSC1
SOX2_diffGCSC2
Vimentin_diffGCSC1
Vimentin_diffGCSC2

Figure 3A

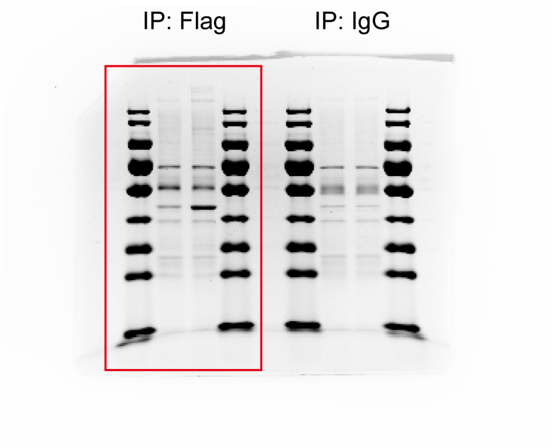

Figure 3D

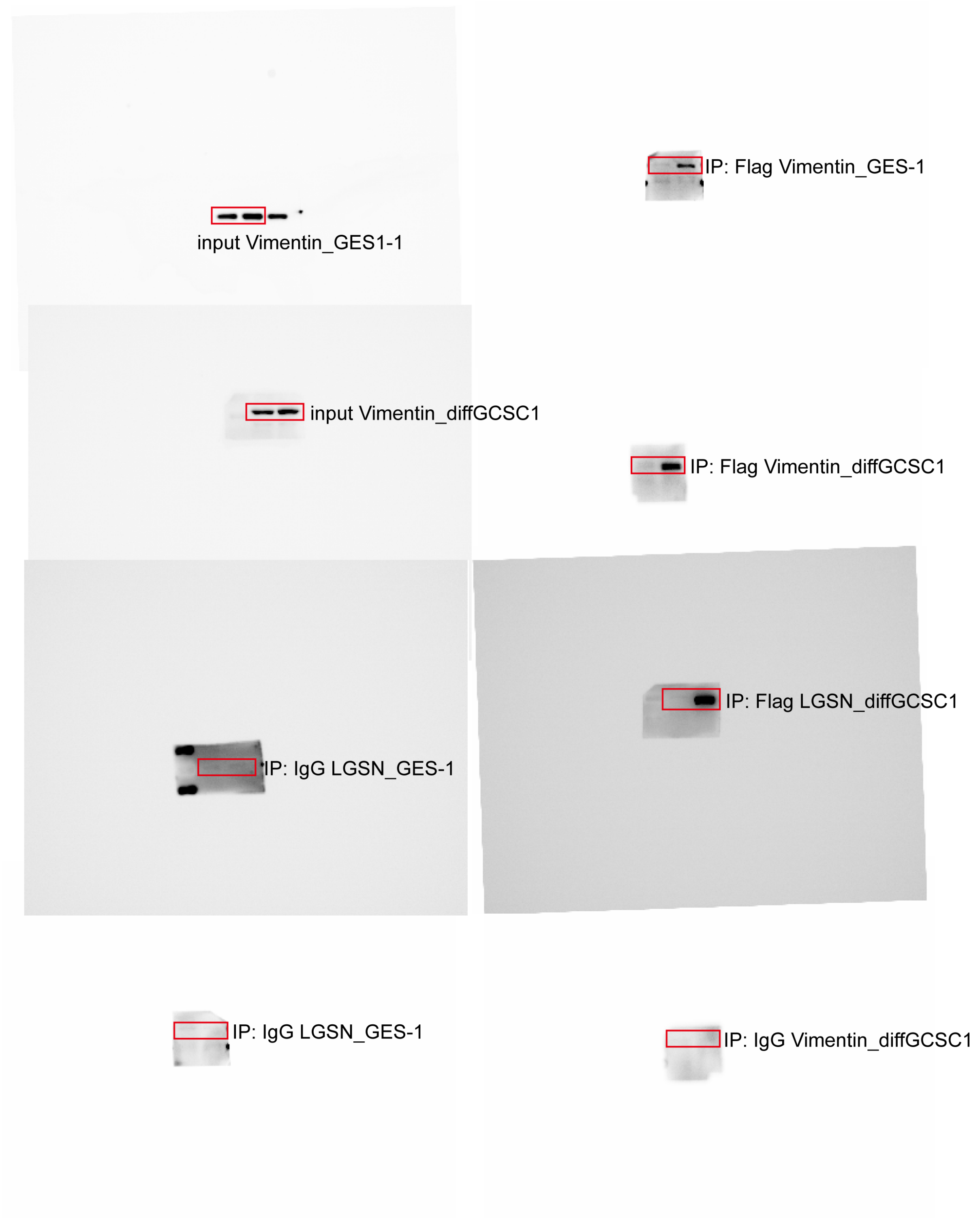

input Vimentin_GES1-1
IP: Flag Vimentin_GES-1
input Vimentin_diffGCSC1
IP: Flag Vimentin_diffGCSC1
IP: Flag LGSN_diffGCSC1
IP: IgG LGSN_GES-1
IP: IgG LGSN_GES-1
IP: IgG Vimentin_diffGCSC1

Figure 3D

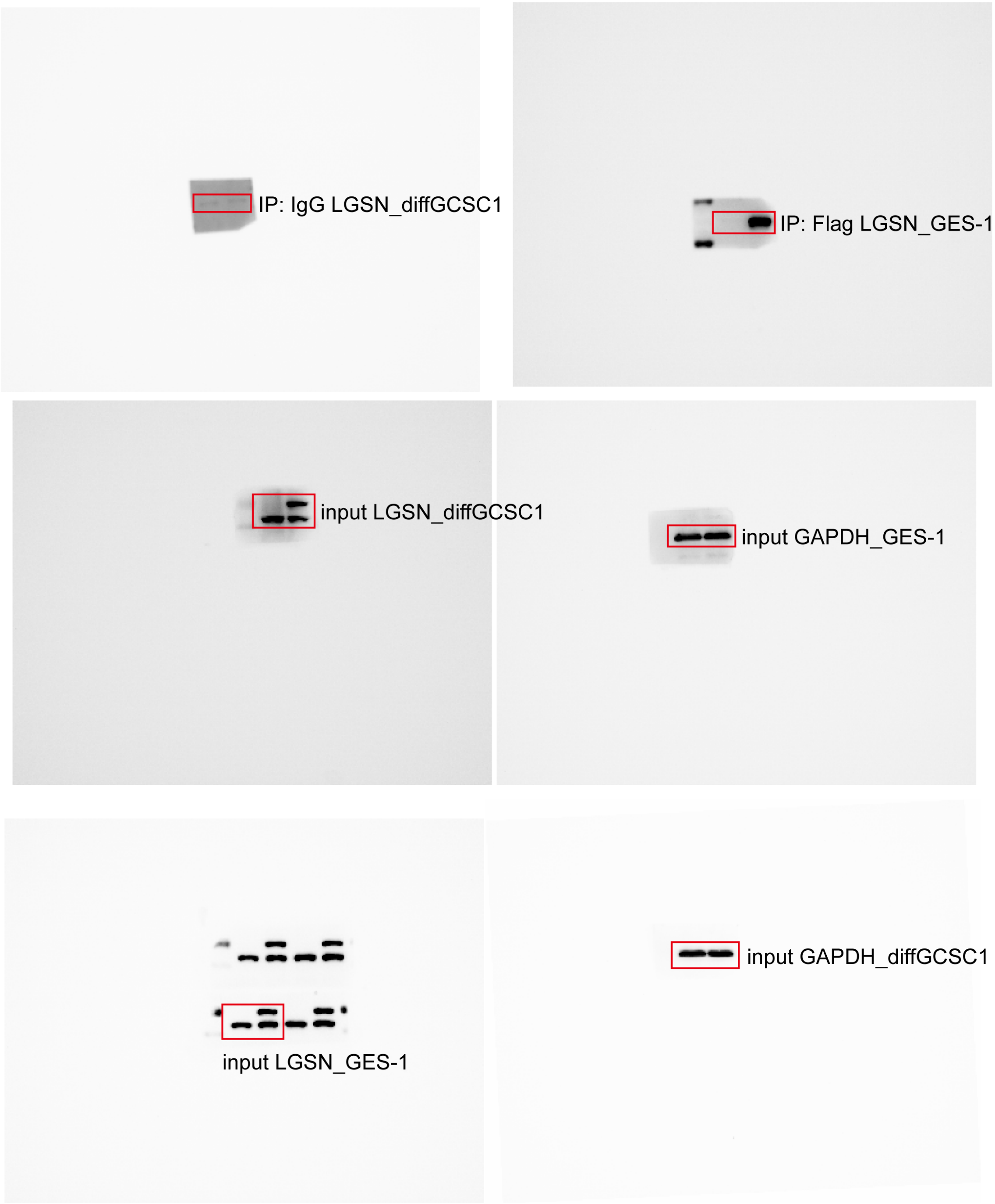
IP: IgG LGSN_diffGCSC1
IP: Flag LGSN_GES-1
input LGSN_diffGCSC1
input GAPDH_GES-1
input LGSN_GES-1
input GAPDH_diffGCSC1

Figure 3E

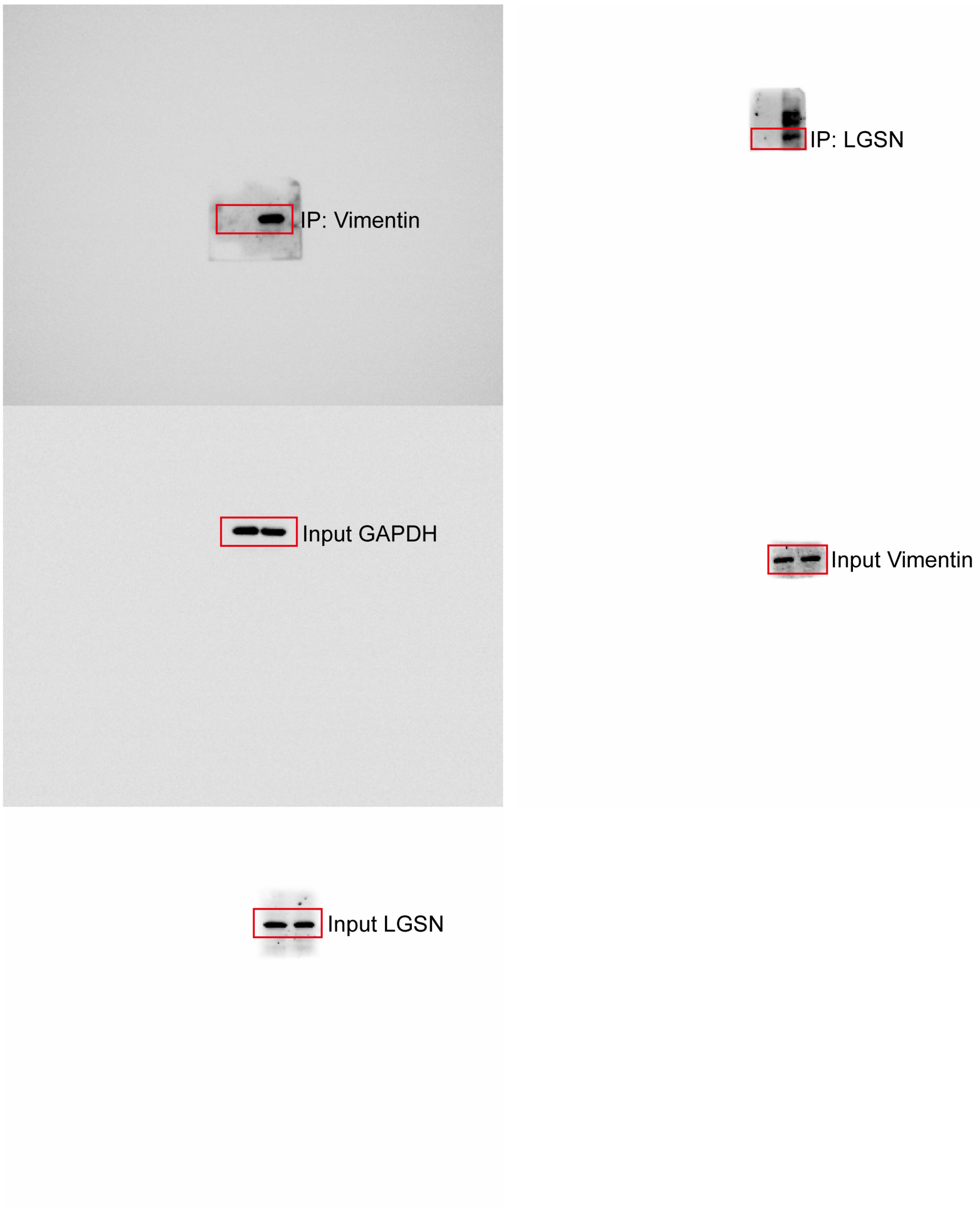

Figure 3I

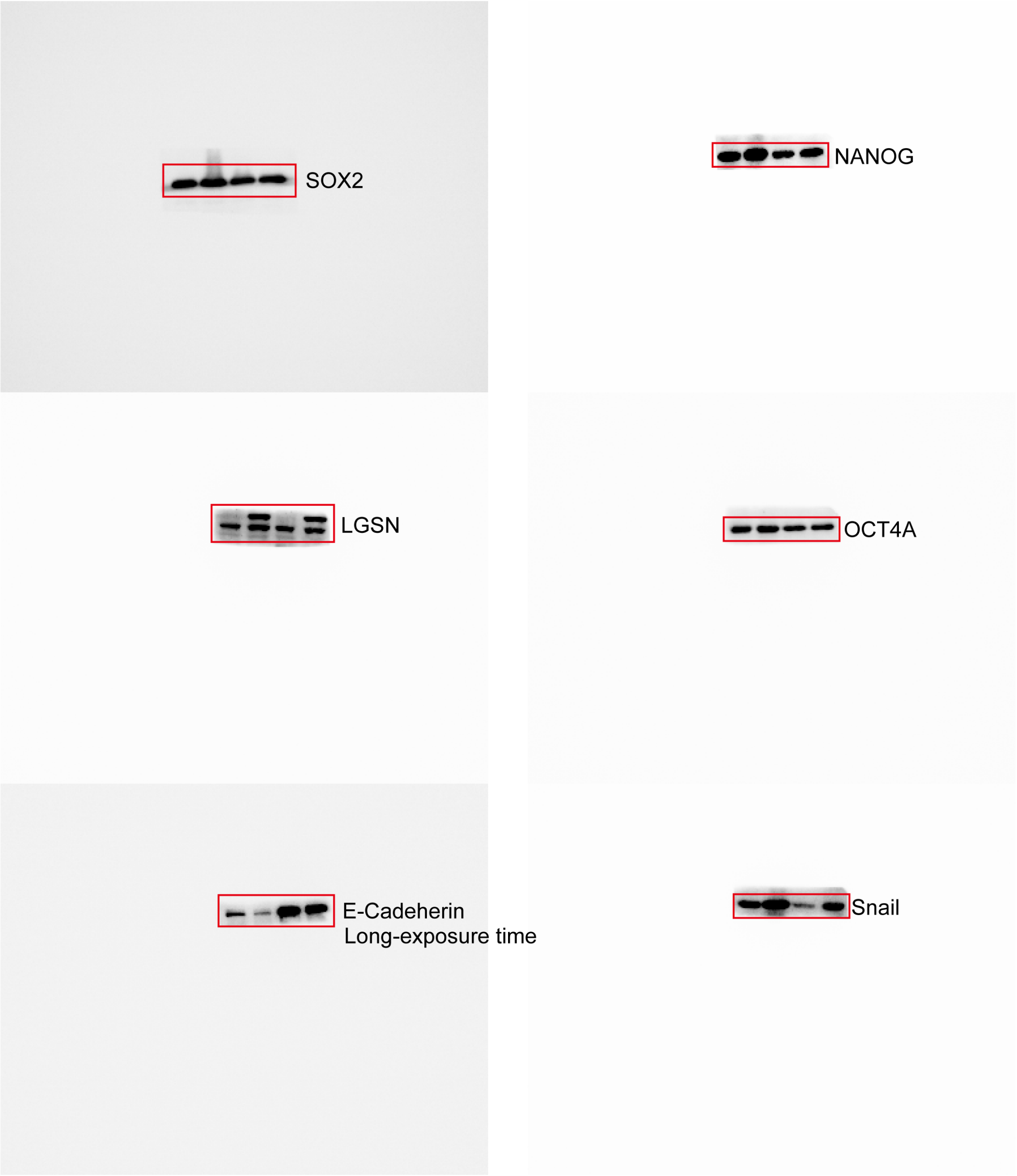

Figure 3I

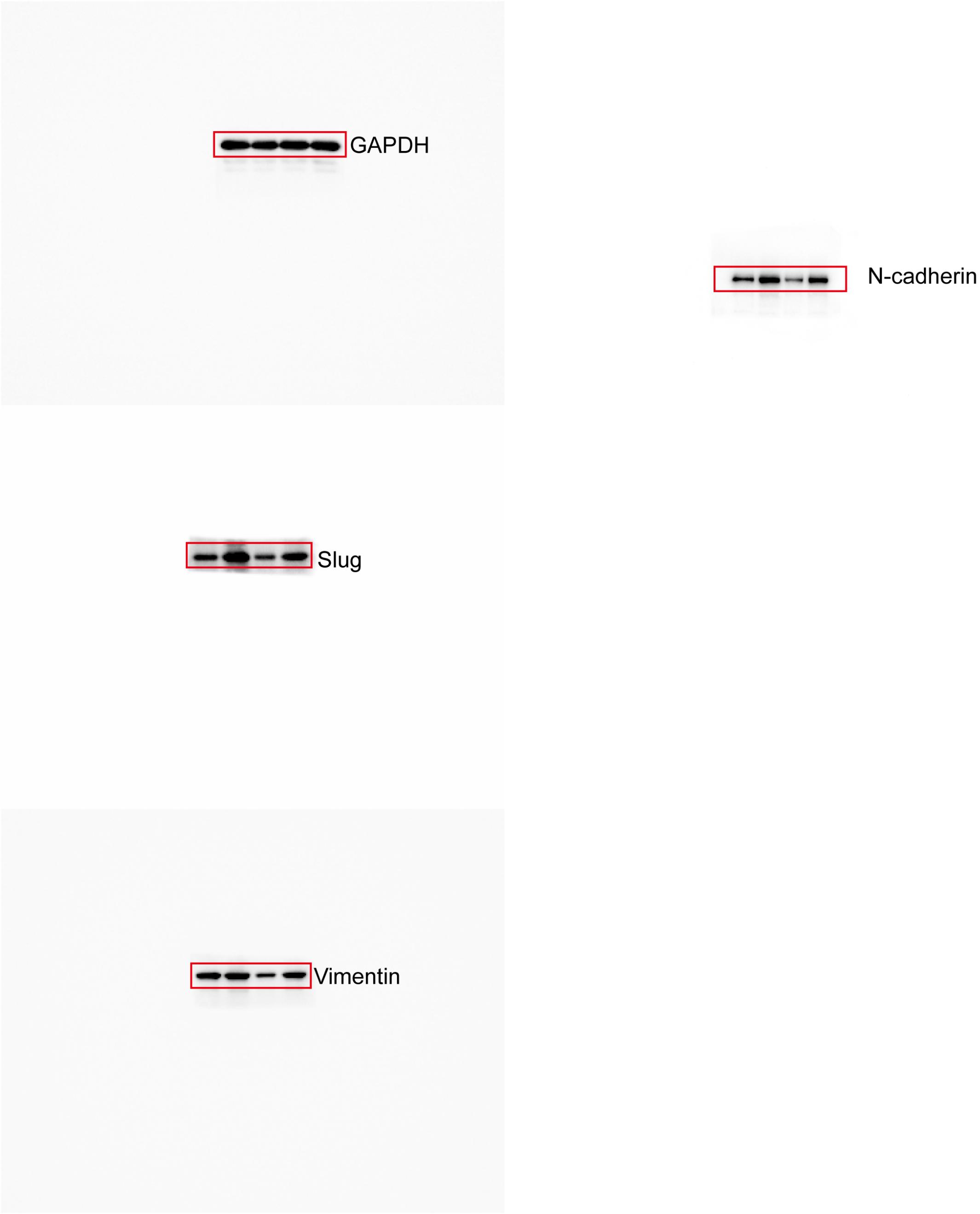

Figure 4B

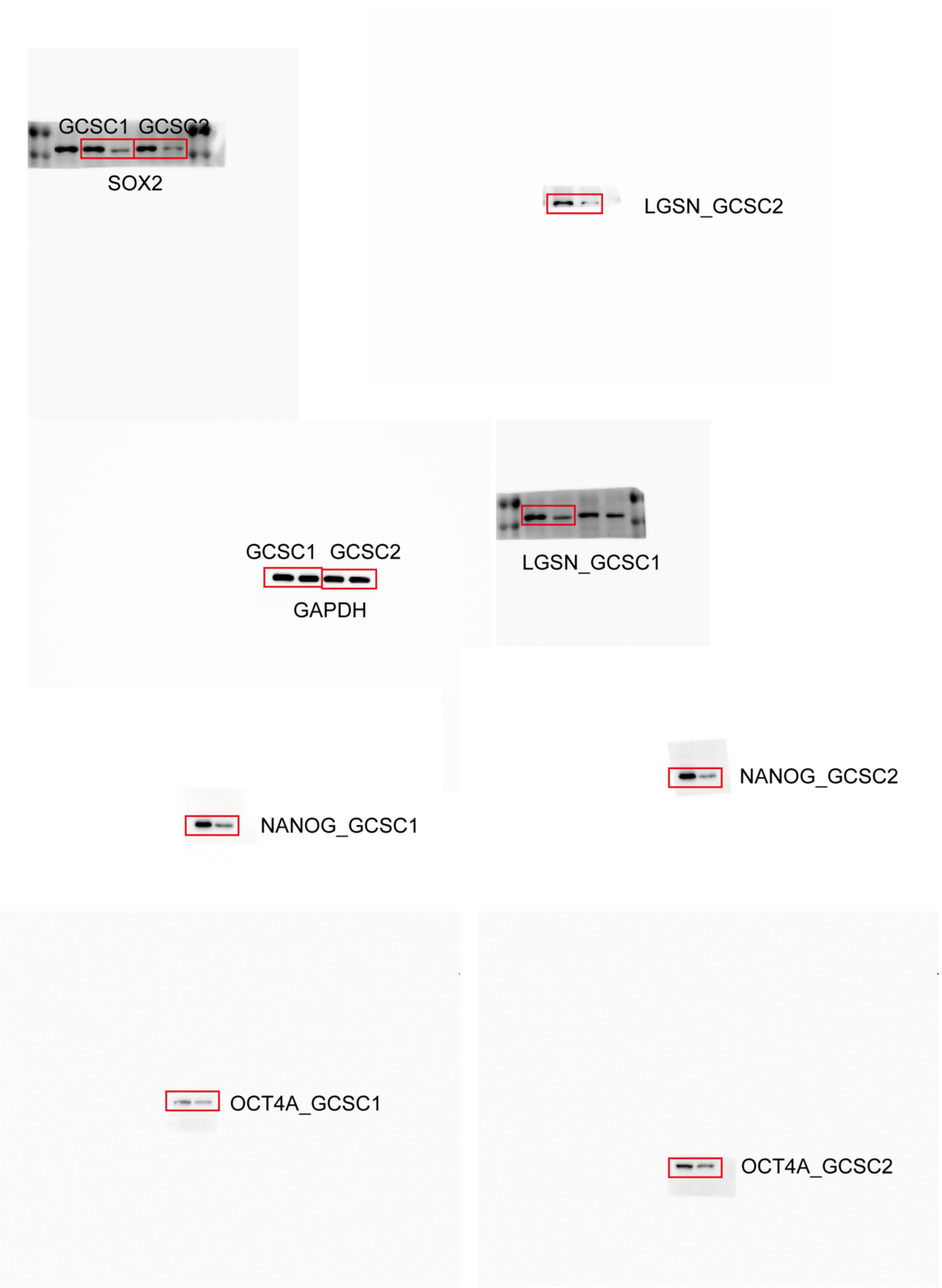
GCSC1 GCSC2
SOX2
LGSN_GCSC2
GCSC1 GCSC2
GAPDH
LGSN_GCSC1
NANOG_GCSC2
NANOG_GCSC1
OCT4A_GCSC1
OCT4A_GCSC2

Figure 5C

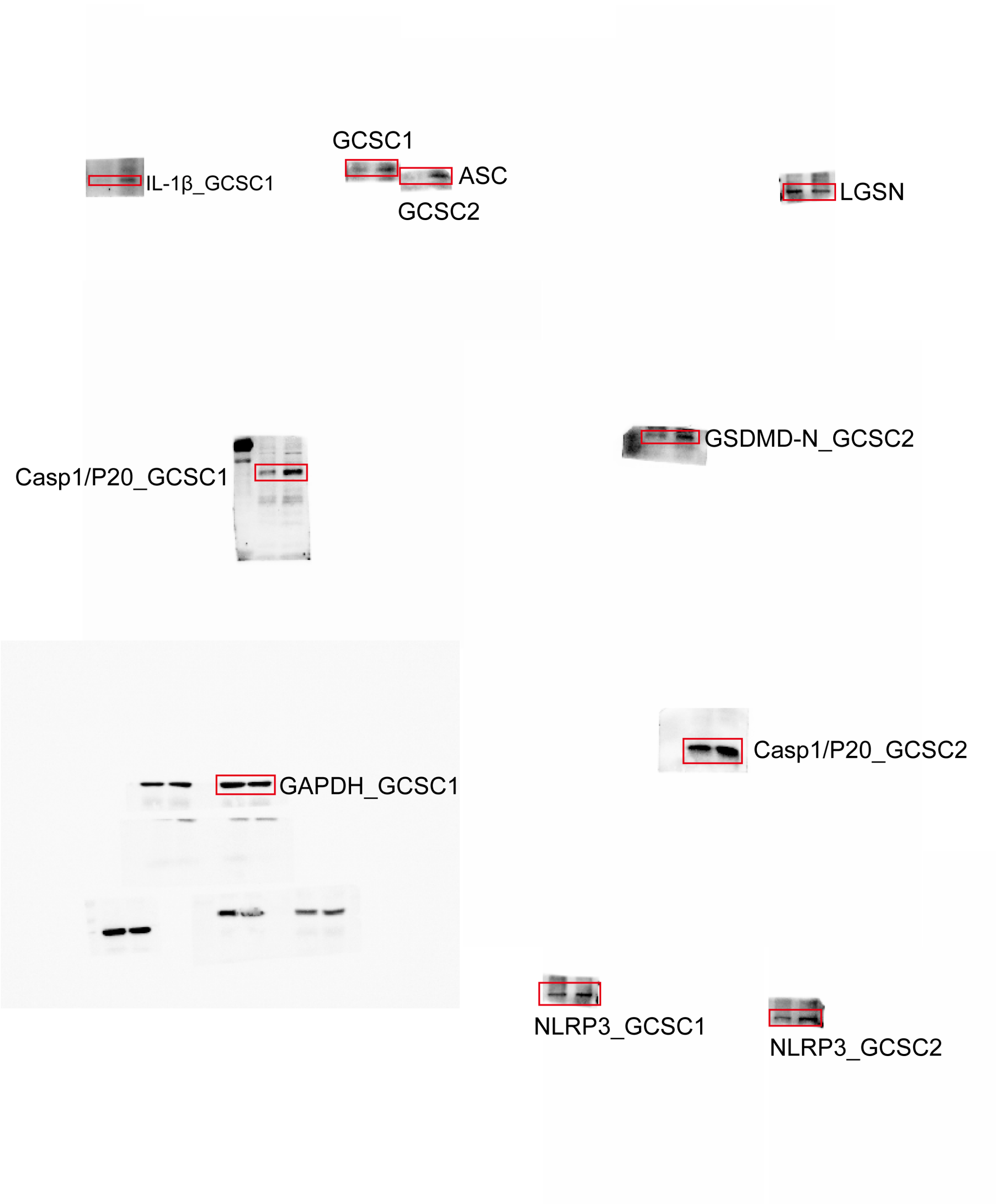

GCSC1
IL-1β_GCSC1
ASC
GCSC2
LGSN
GSDMD-N_GCSC2
Casp1/P20_GCSC1
Casp1/P20_GCSC2
GAPDH_GCSC1
NLRP3_GCSC1
NLRP3_GCSC2

Figure 5C

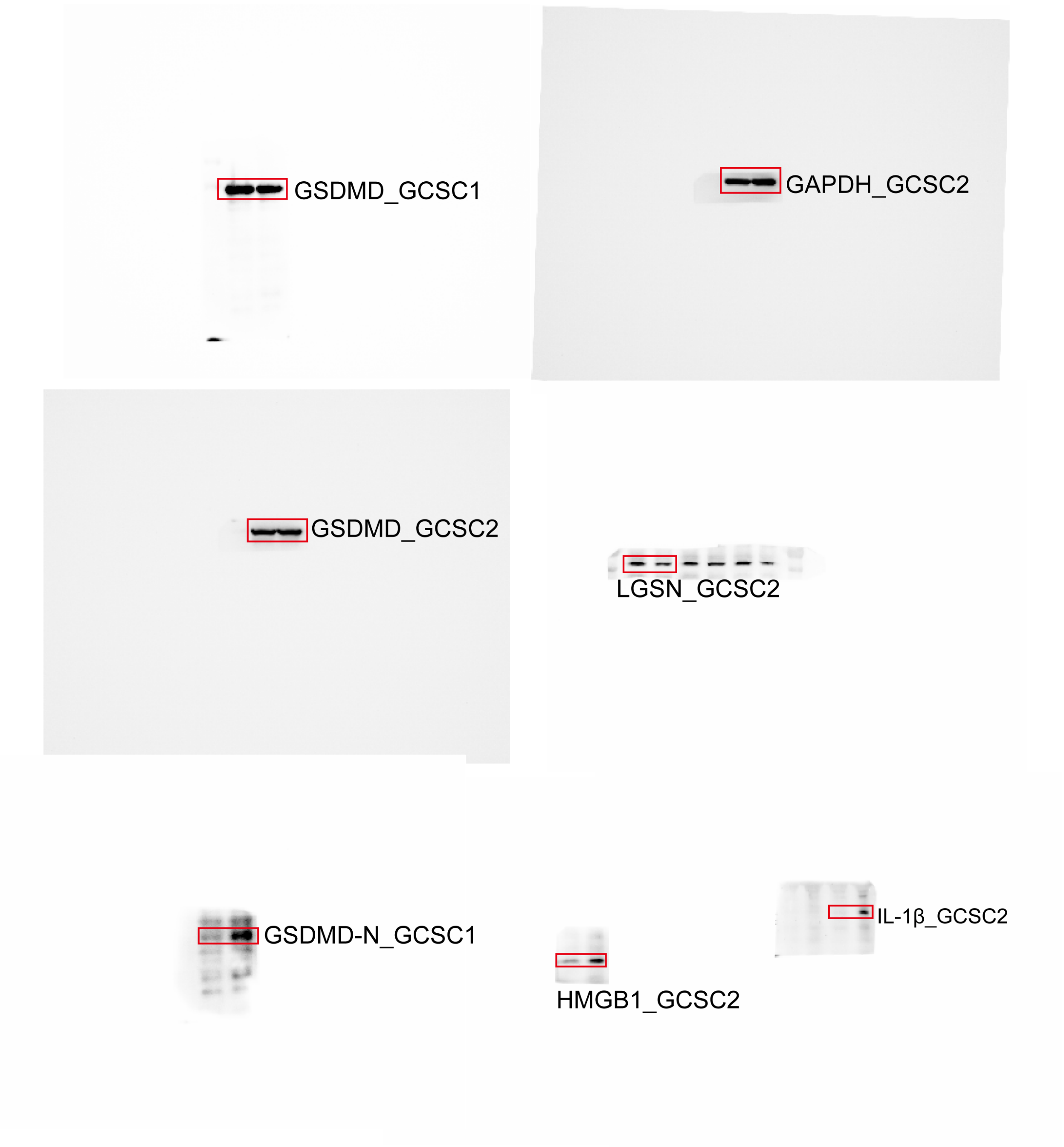
GSDMD_GCSC1
GAPDH_GCSC2
GSDMD_GCSC2
LGSN_GCSC2
GSDMD-N_GCSC1
HMGB1_GCSC2
IL-1β_GCSC2

Figure 5C

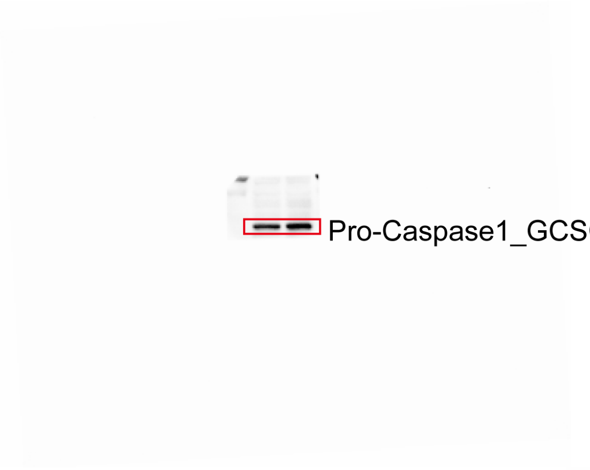
Pro-Caspase1_GCSC2

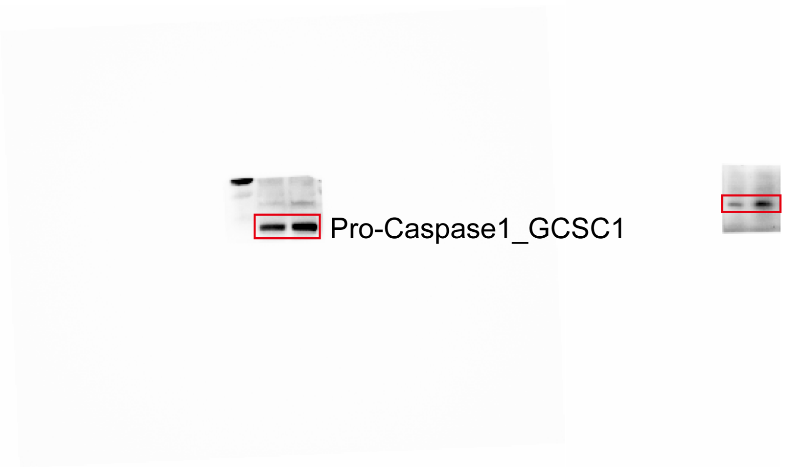
Pro-Caspase1_GCSC1
HMGB1_GCSC1

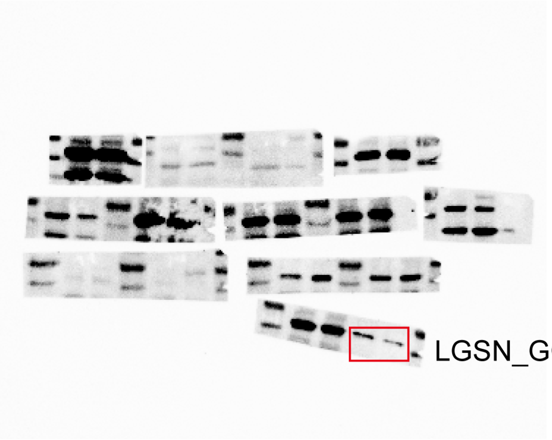
LGSN_GCSC1

Figure 5E

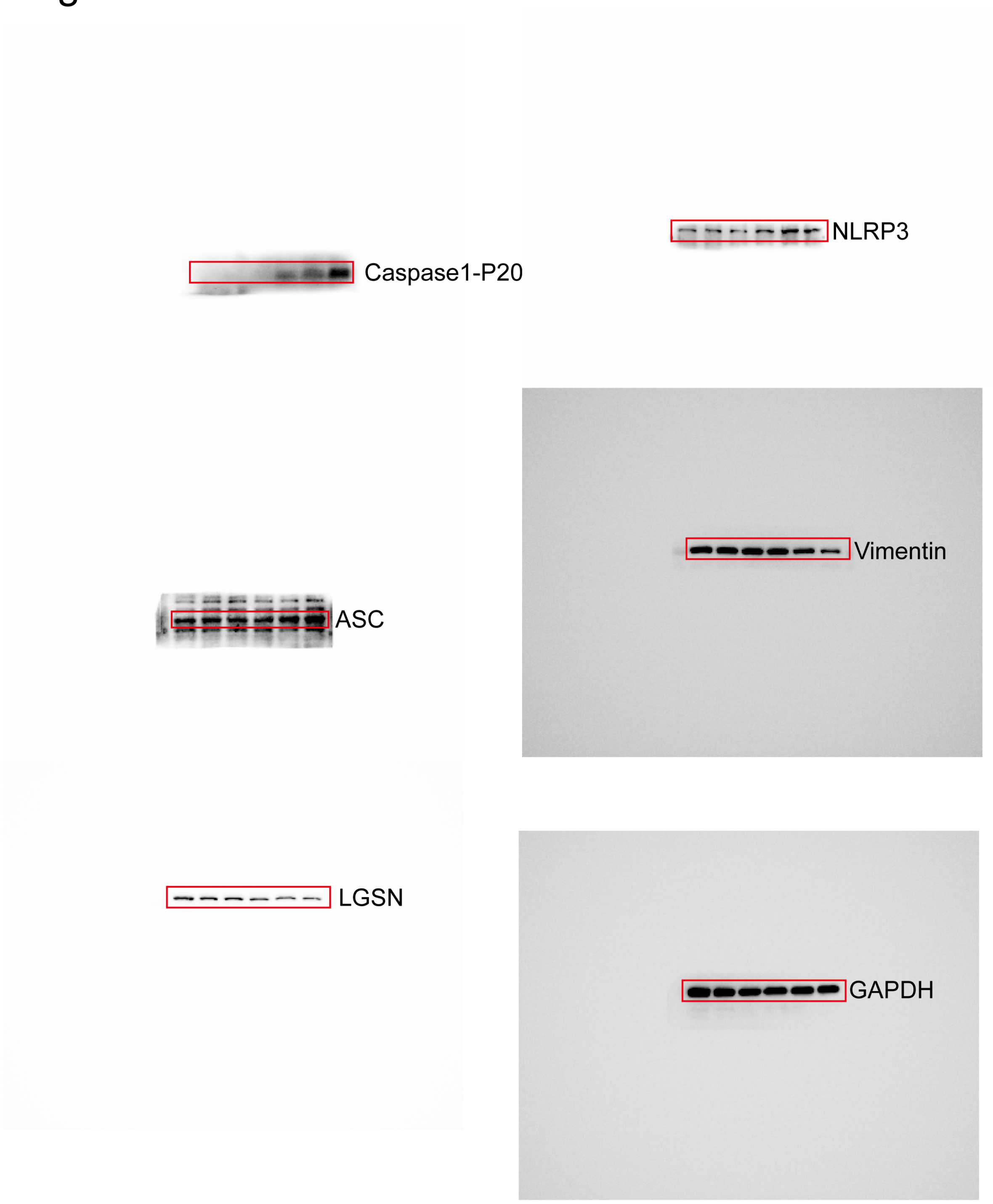

Figure 5F

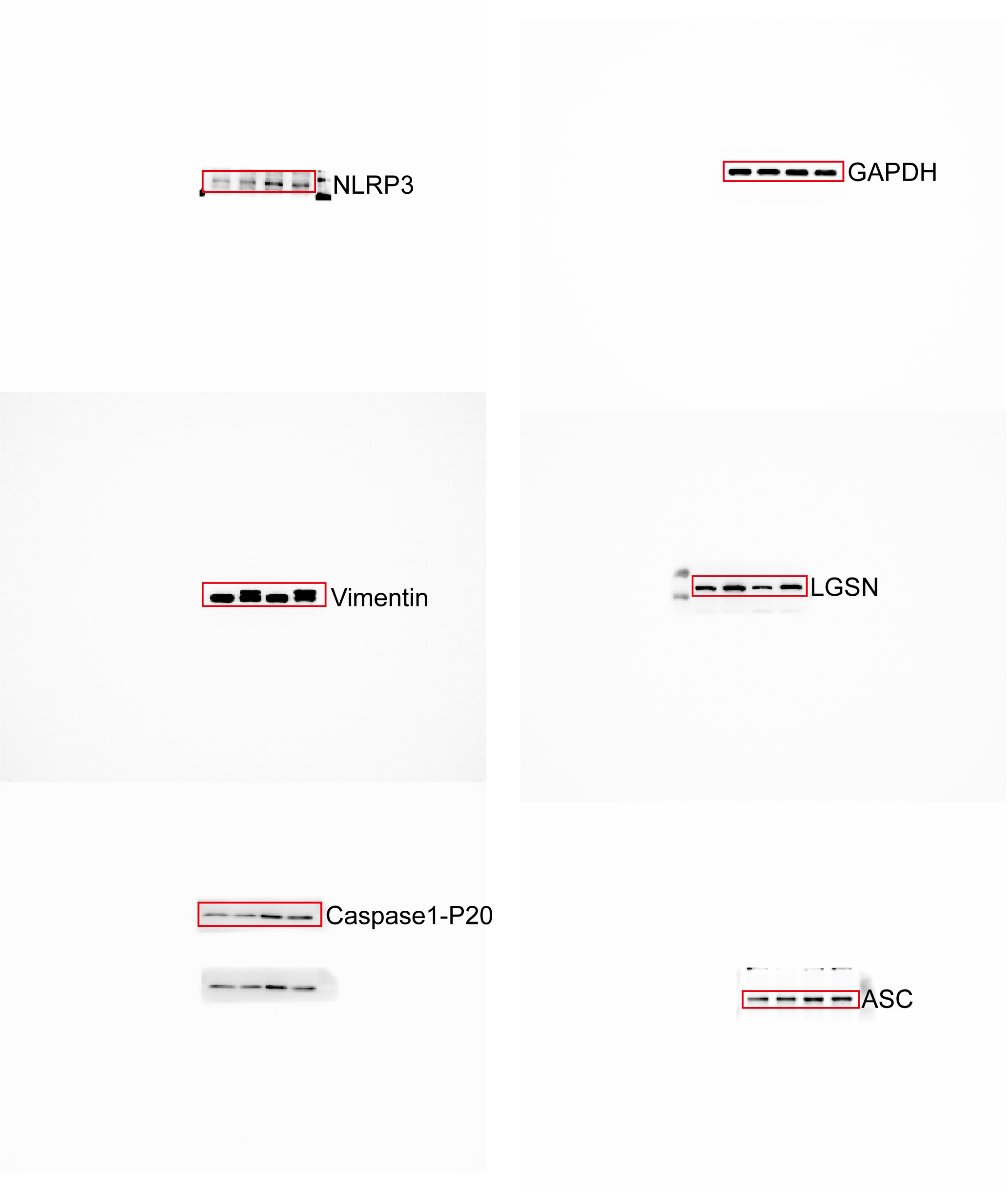

Figure 5G

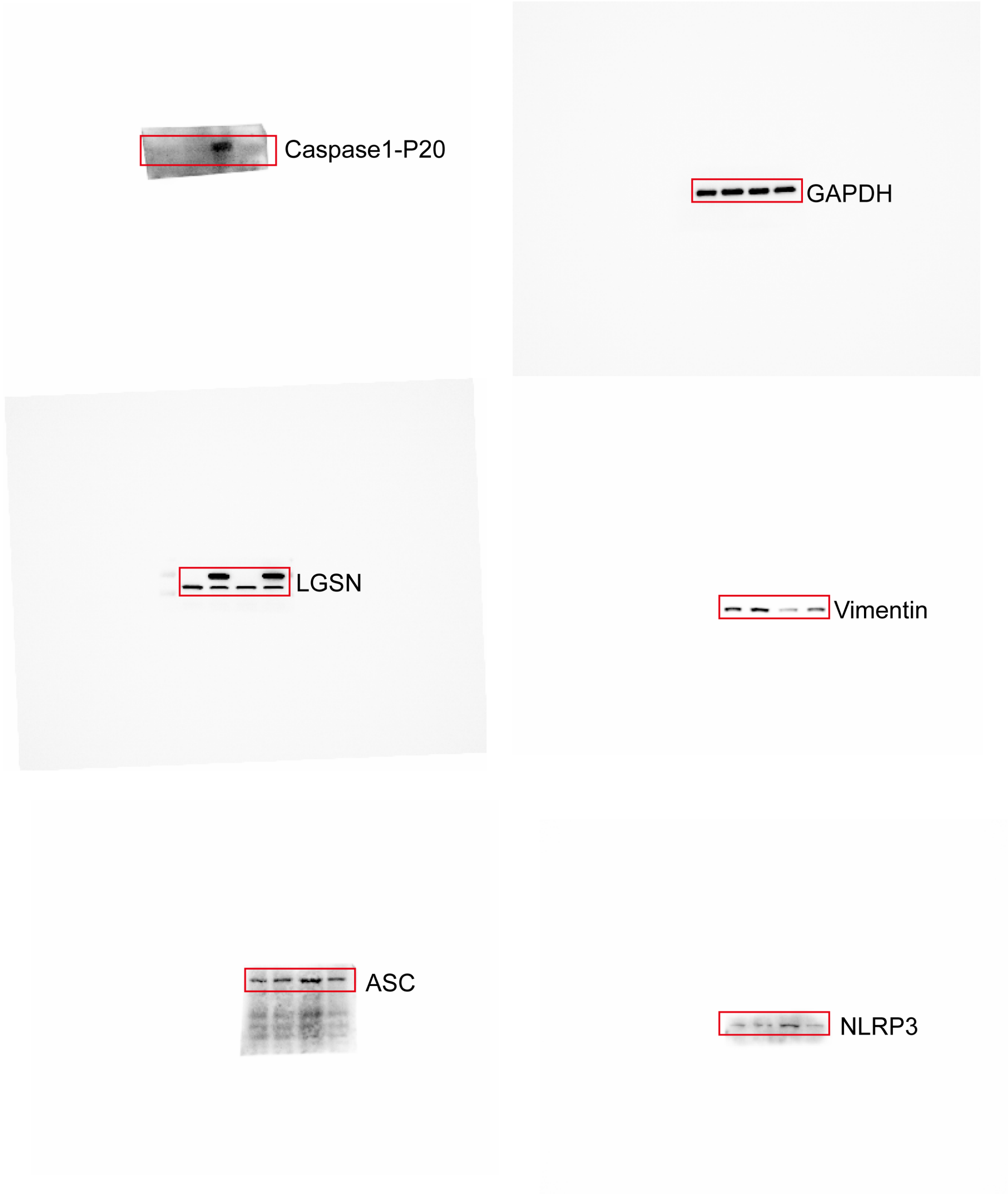

Figure 5H

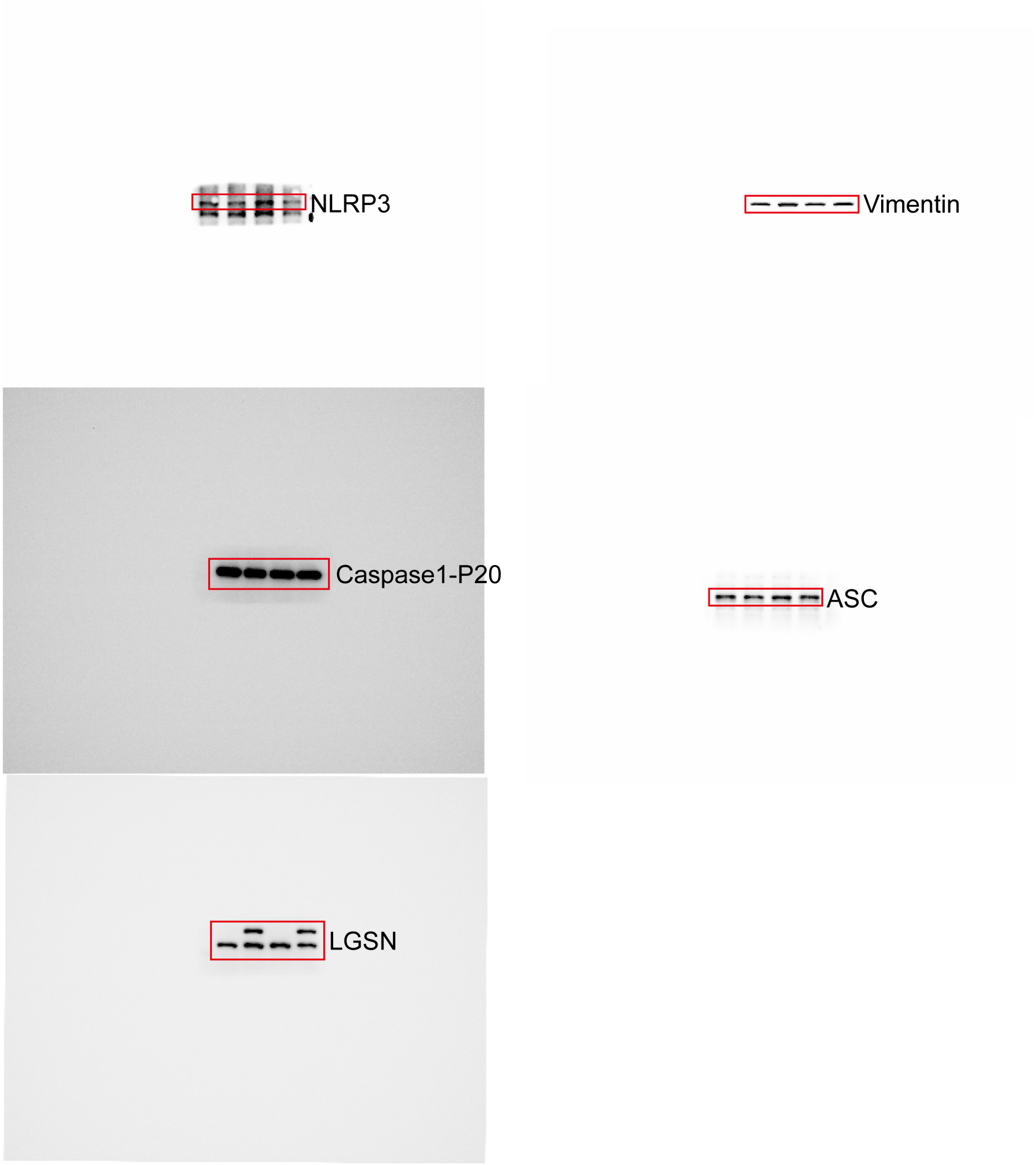

Figure 6G

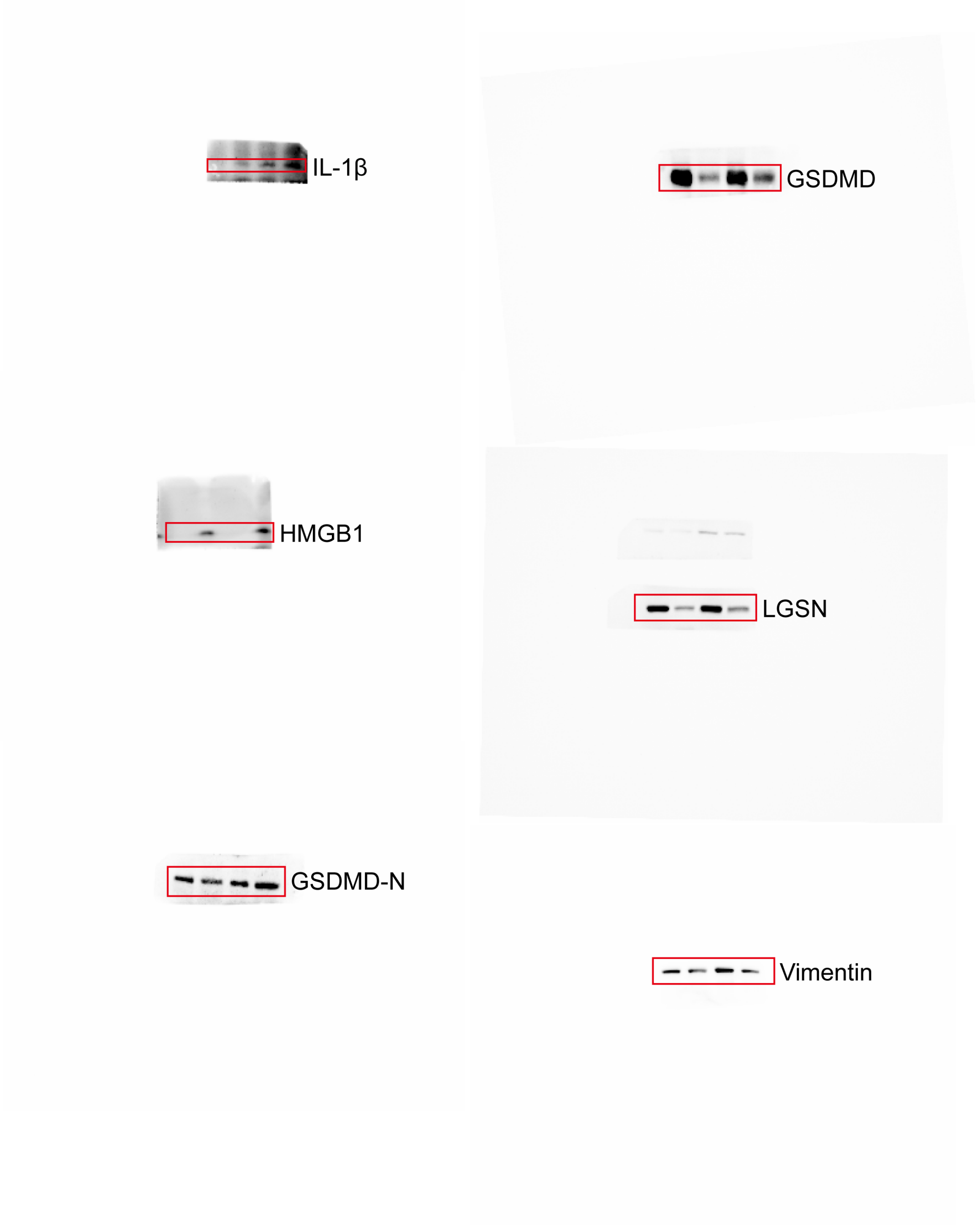

Figure 6G

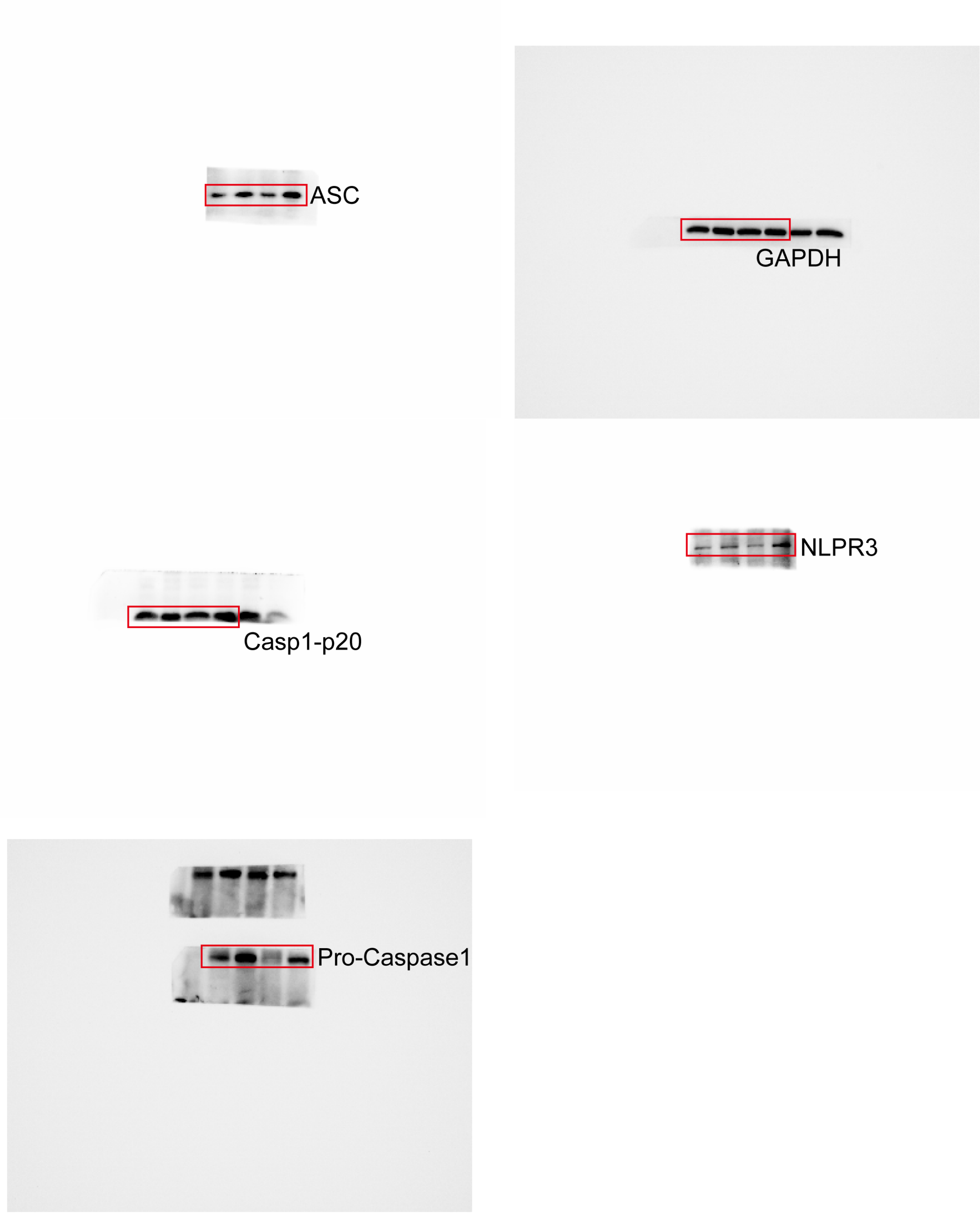

Figure 6I

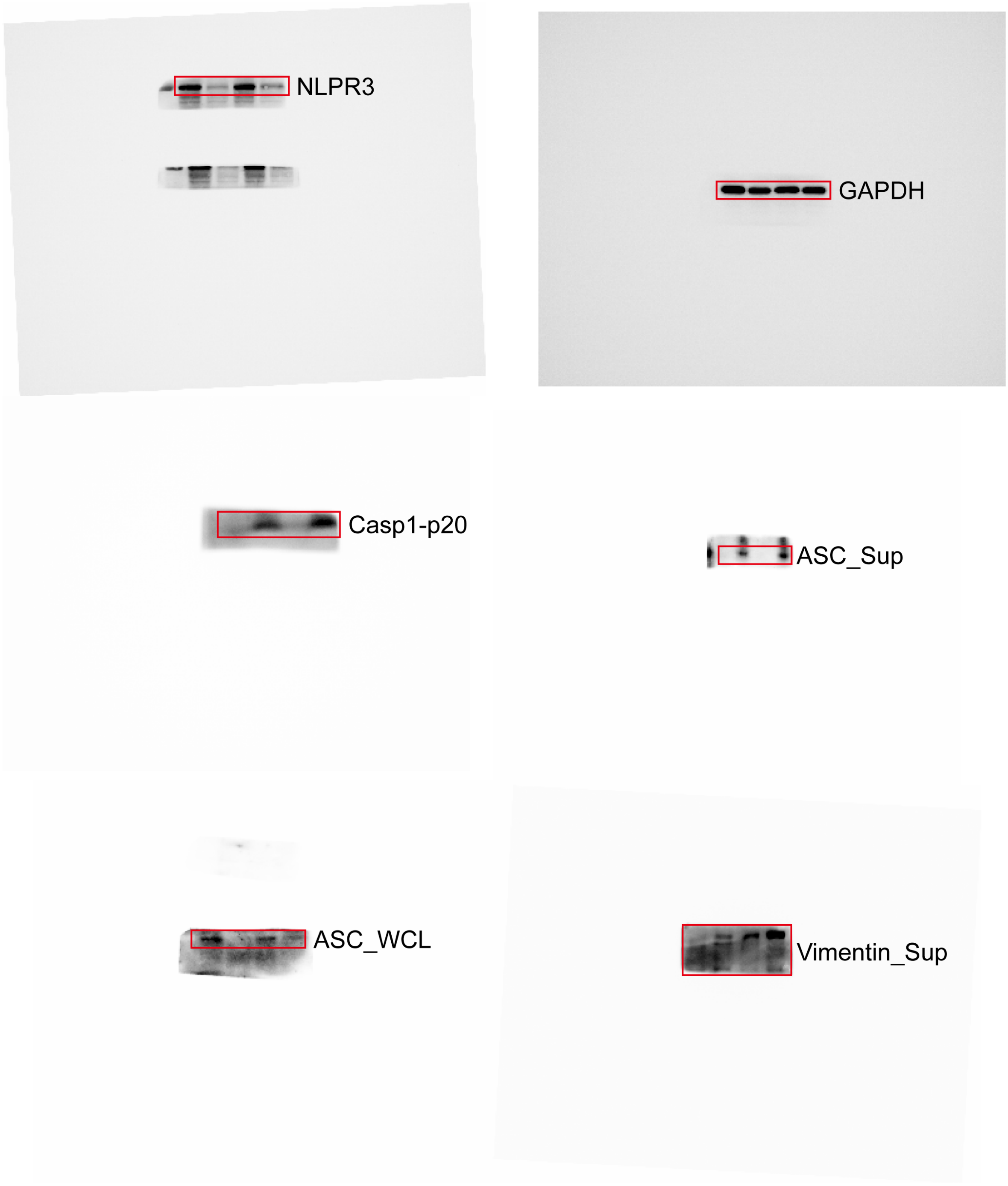

Figure 6I

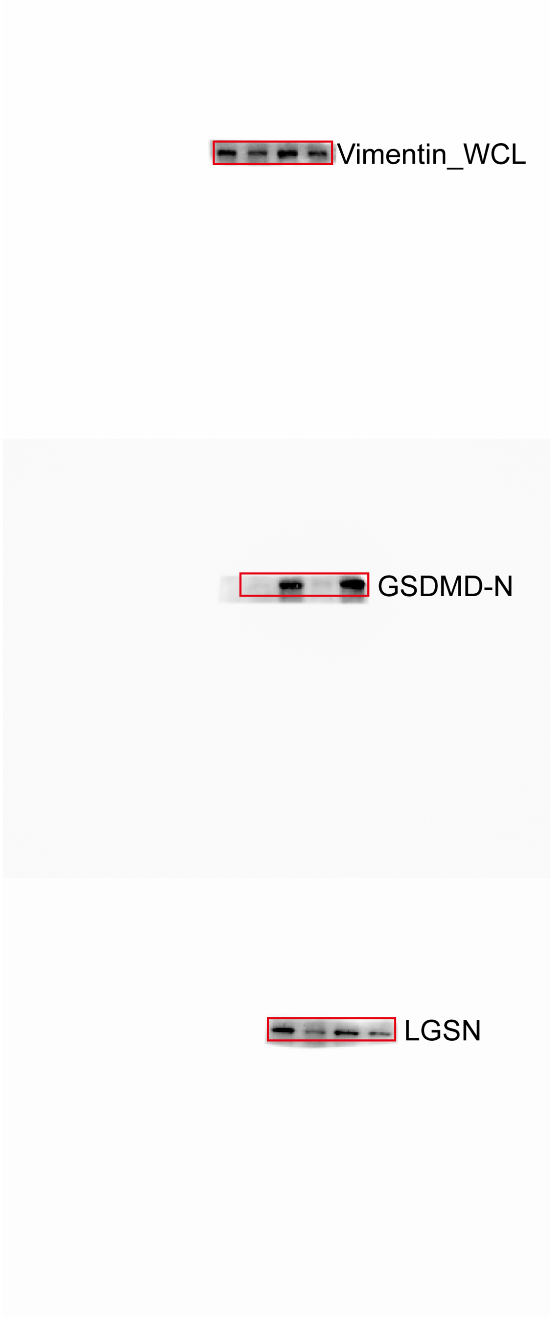

Figure S4A

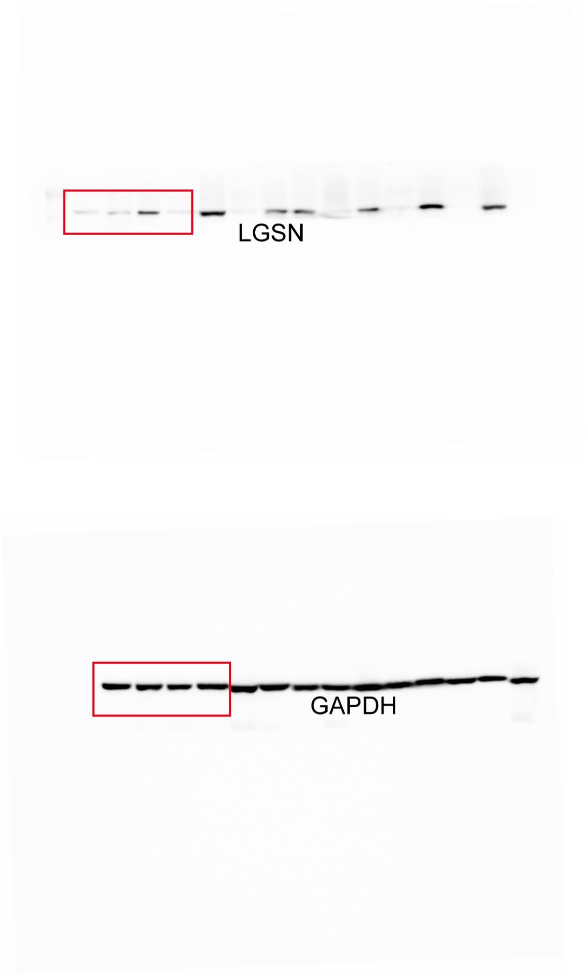

Figure S4C

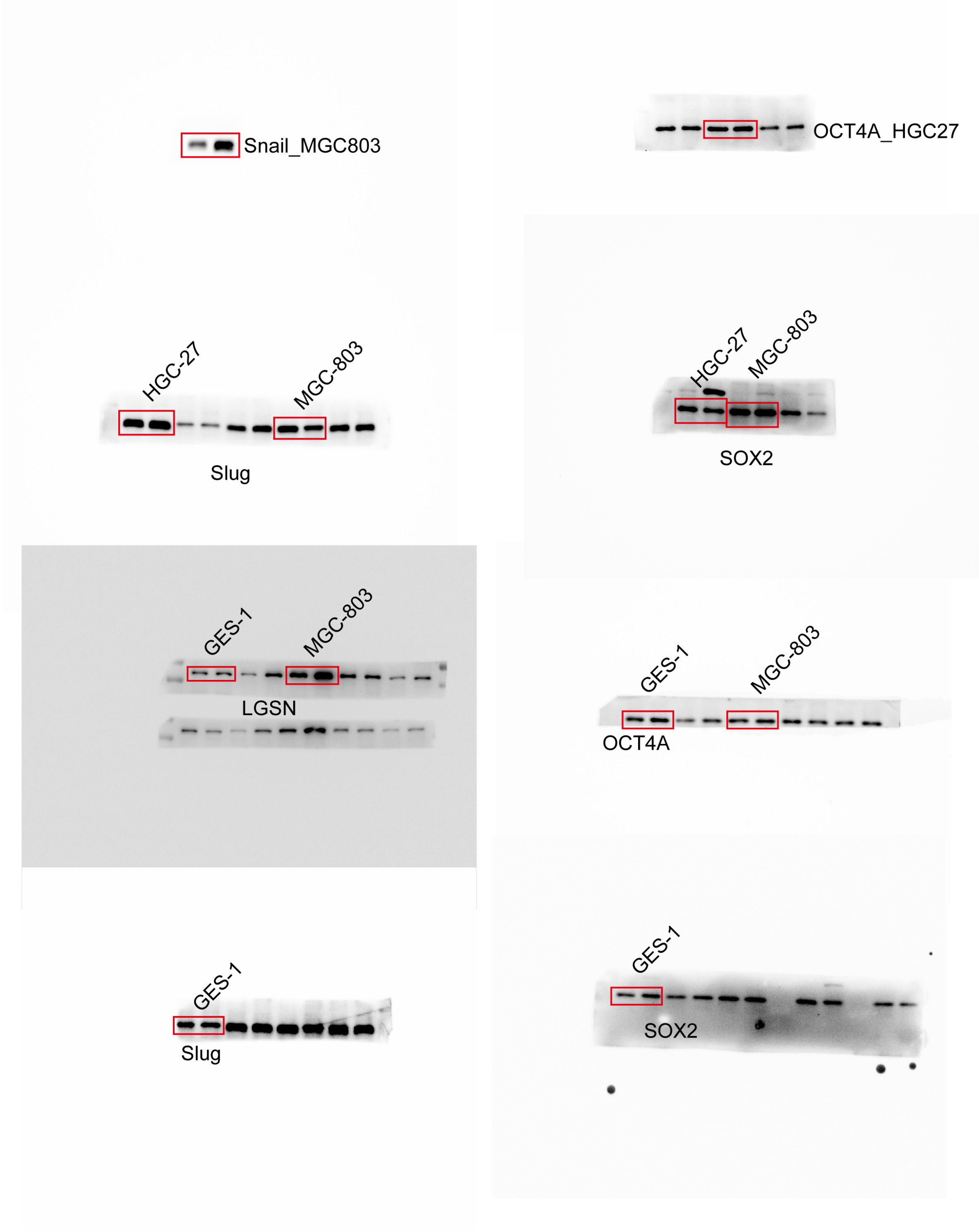

Snail_MGC803
OCT4A_HGC27
HGC-27
MGC-803
Slug
HGC-27
MGC-803
SOX2
GES-1
MGC-803
LGSN
GES-1
MGC-803
OCT4A
GES-1
Slug
GES-1
SOX2

Figure S4C

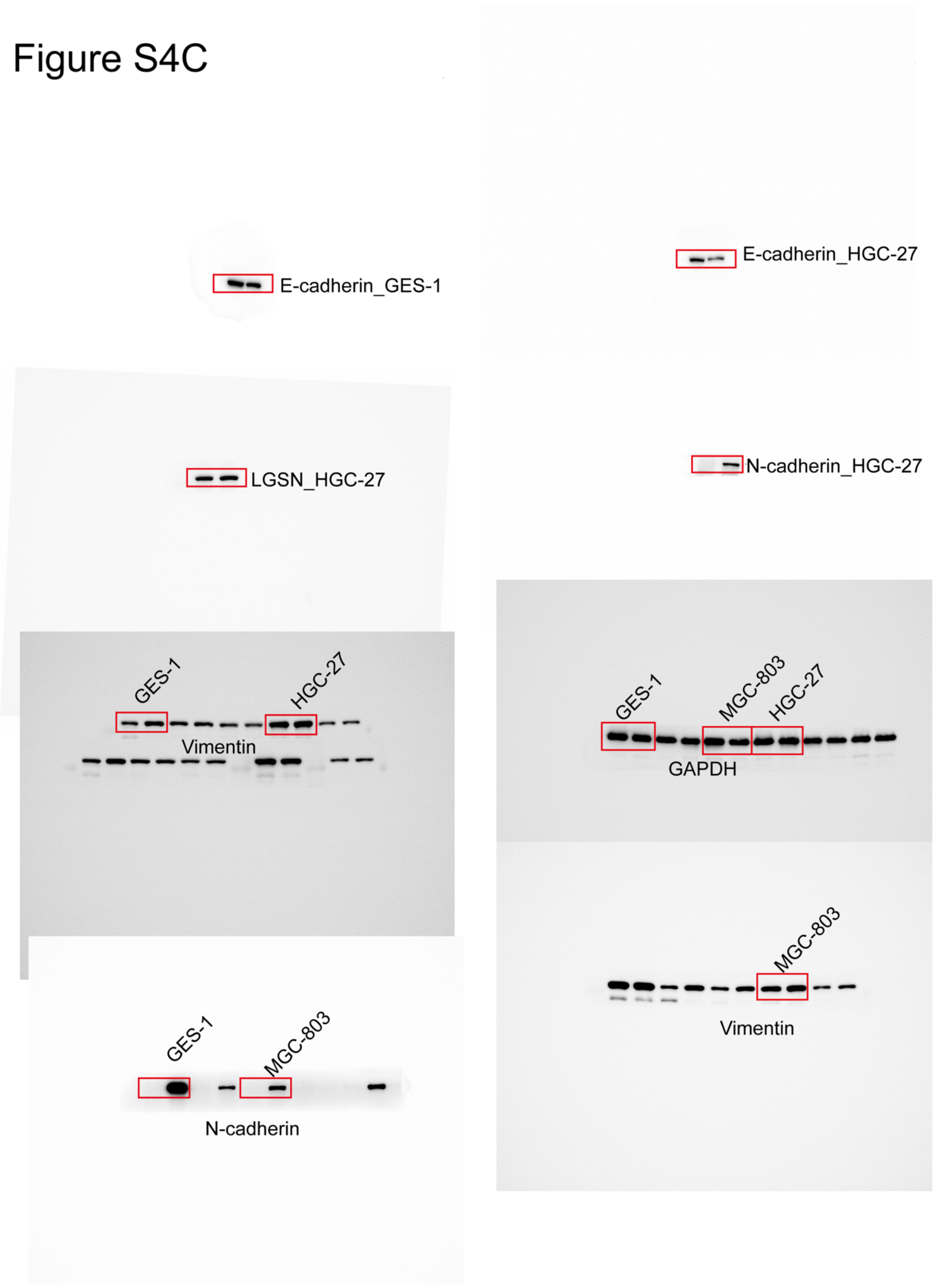

E-cadherin_GES-1
E-cadherin_HGC-27
LGSN_HGC-27
N-cadherin_HGC-27
GES-1
HGC-27
Vimentin
GES-1
MGC-803
HGC-27
GAPDH
GES-1
MGC-803
N-cadherin
MGC-803
Vimentin

Figure S4C

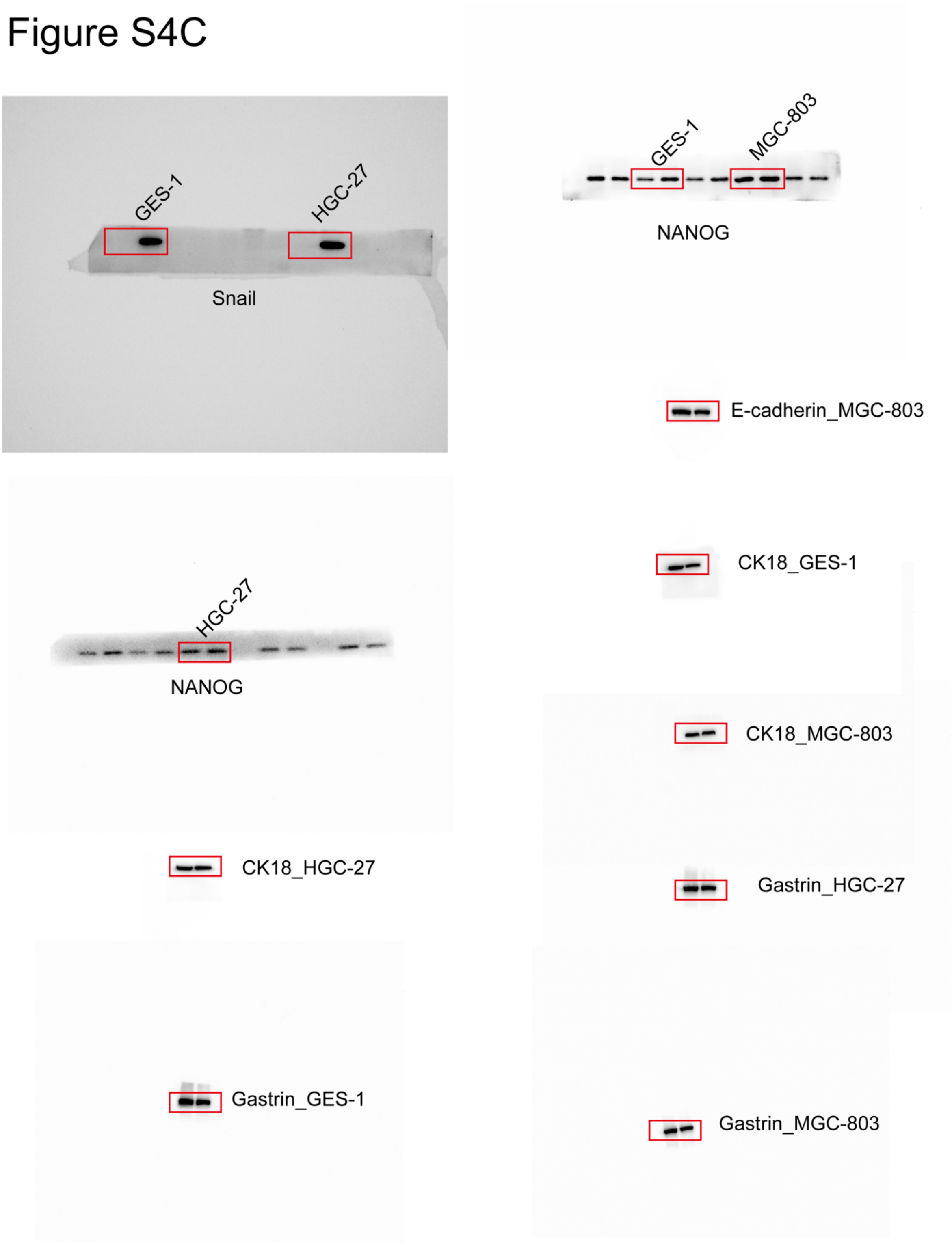
GES-1
HGC-27
Snail
GES-1
MGC-803
NANOG
E-cadherin_MGC-803
CK18_GES-1
HGC-27
NANOG
CK18_MGC-803
CK18_HGC-27
Gastrin_HGC-27
Gastrin_GES-1
Gastrin_MGC-803

Figure S4D

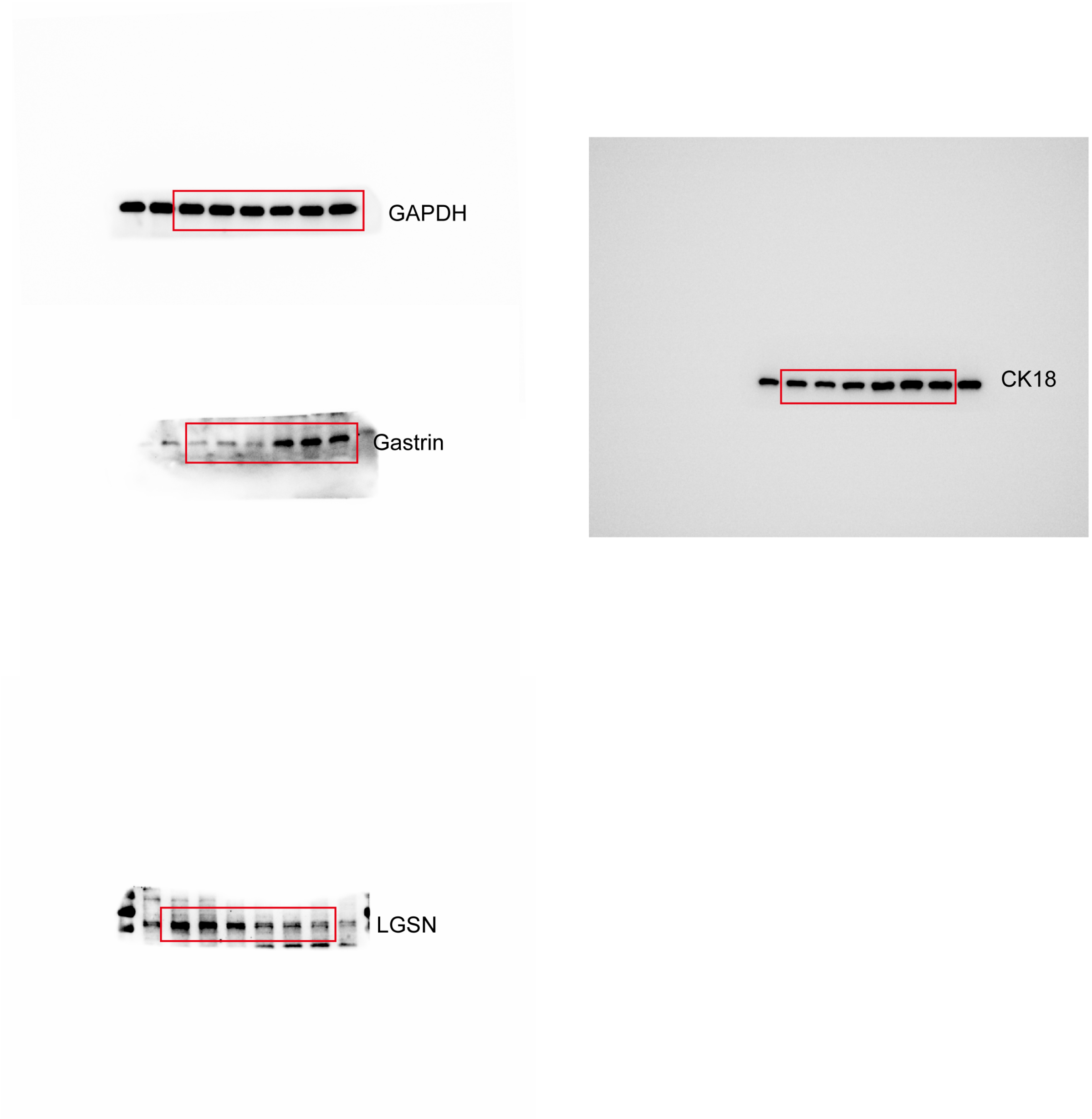

Figure S8A

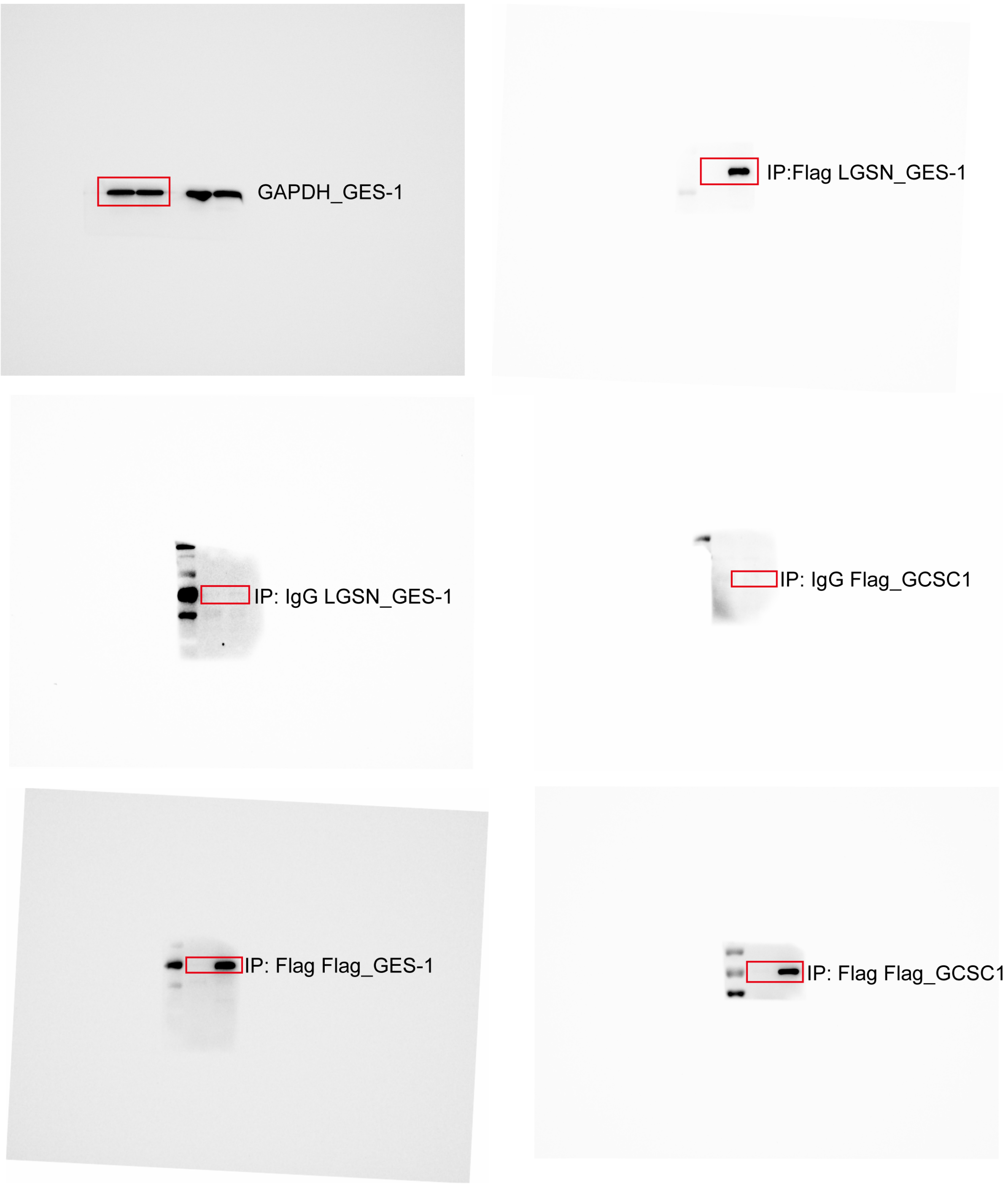

GAPDH_GES-1
IP:Flag LGSN_GES-1
IP: IgG LGSN_GES-1
IP: IgG Flag_GCSC1
IP: Flag Flag_GES-1
IP: Flag Flag_GCSC1

Figure S8A

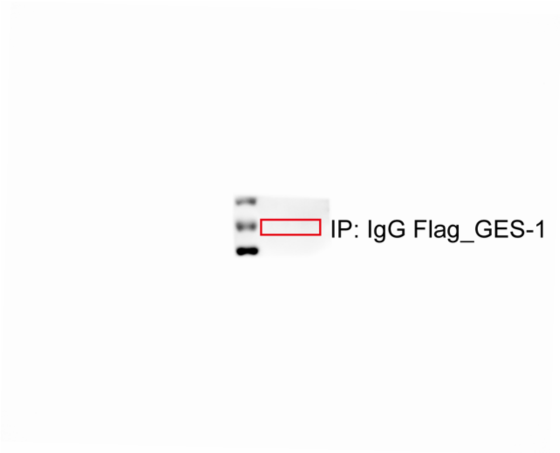
IP: IgG Flag_GES-1

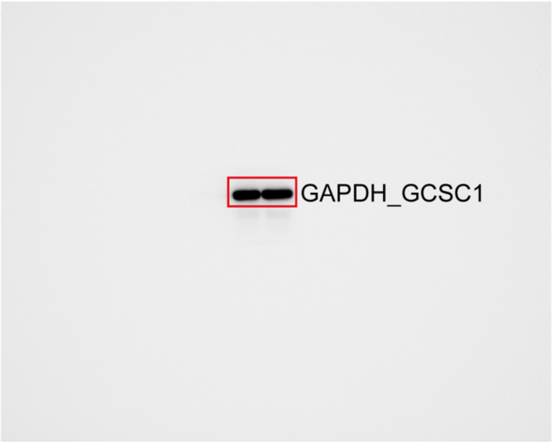
GAPDH_GCSC1

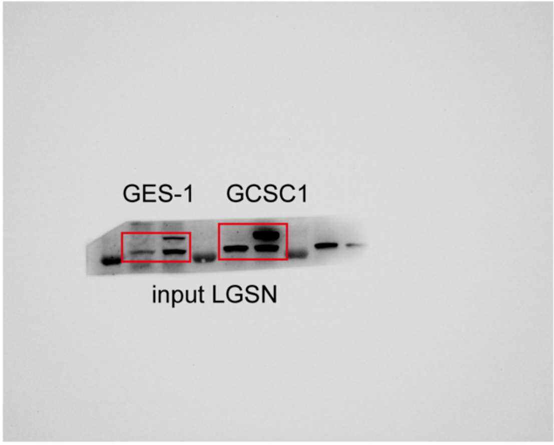
GES-1
GCSC1
input LGSN

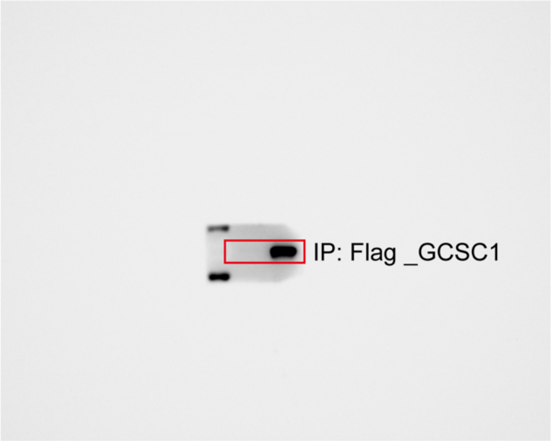
IP: Flag _GCSC1

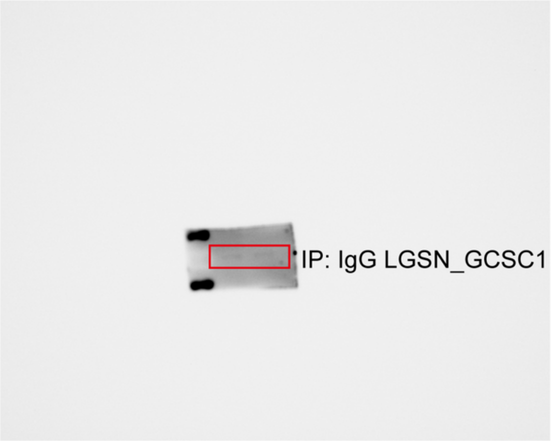
IP: IgG LGSN_GCSC1

Figure S8B

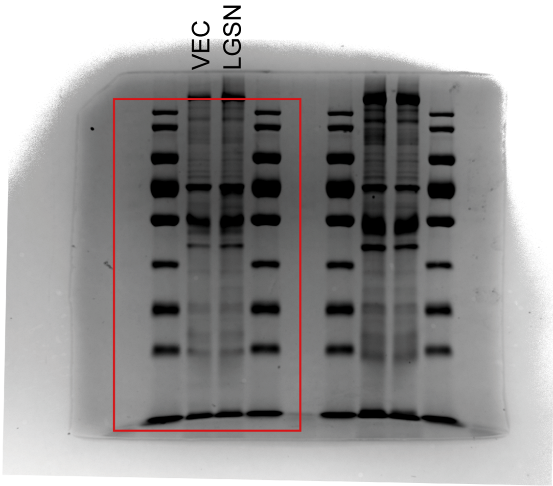

Figure S9D

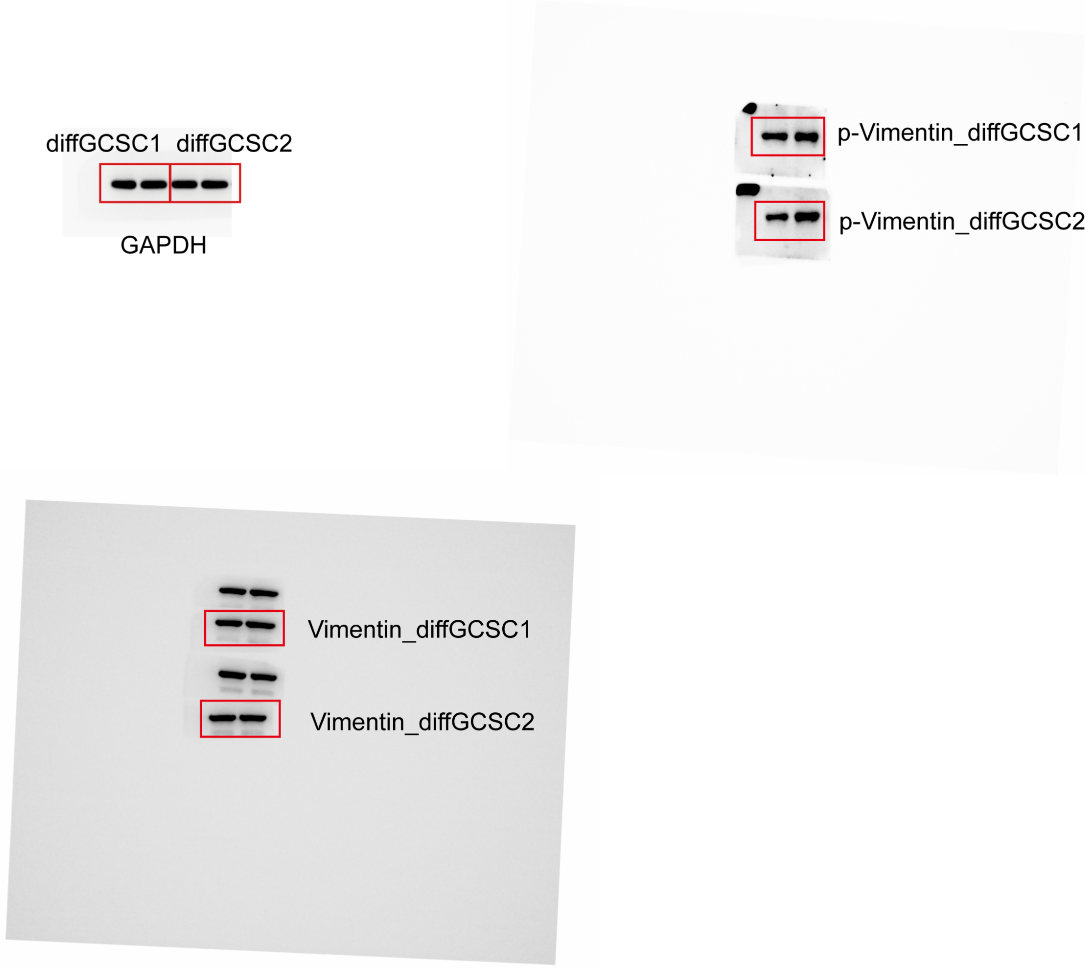
diffGCSC1 diffGCSC2
GAPDH
p-Vimentin_diffGCSC1
p-Vimentin_diffGCSC2
Vimentin_diffGCSC1
Vimentin_diffGCSC2

Figure S9E

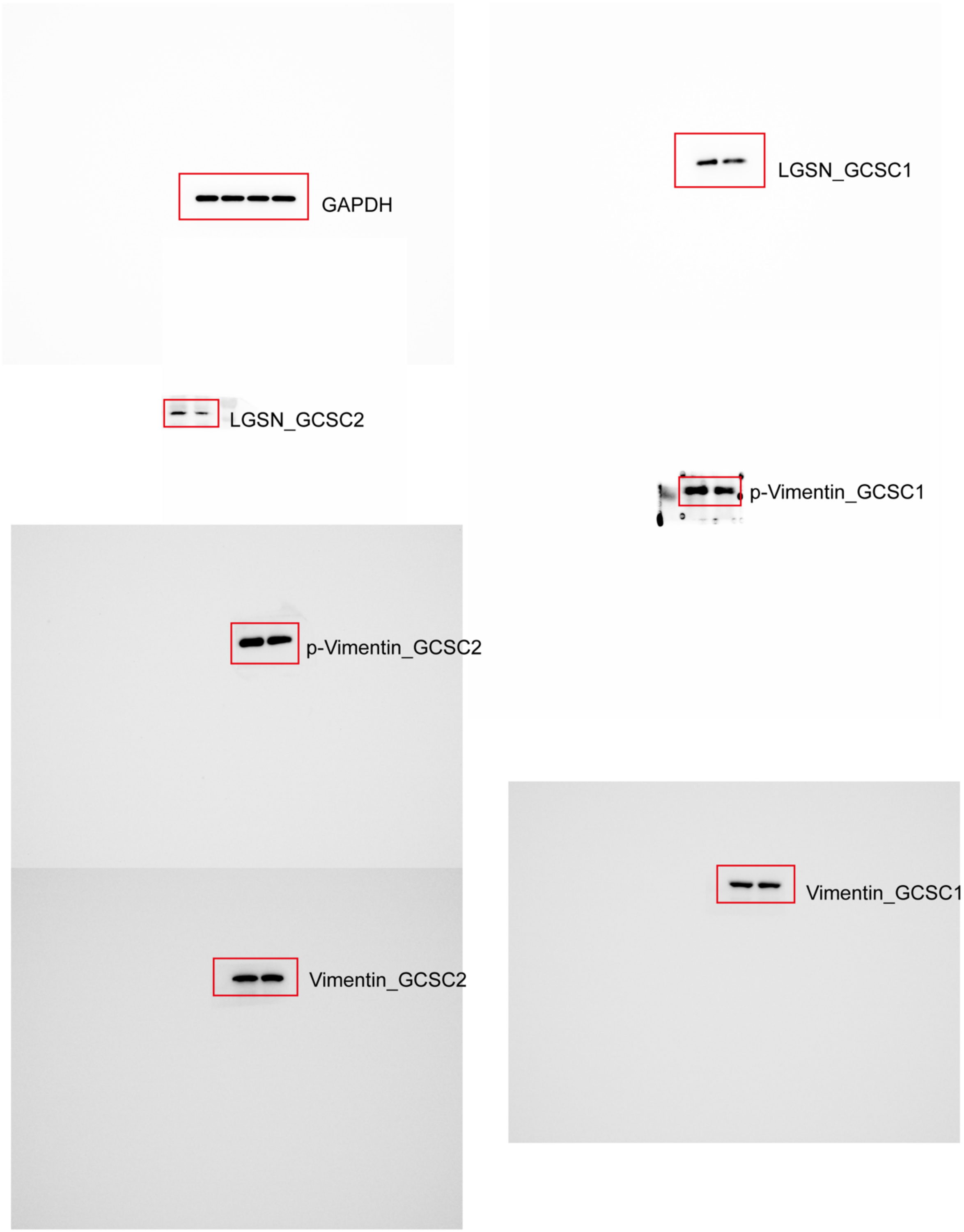
GAPDH
LGSN_GCSC1
LGSN_GCSC2
p-Vimentin_GCSC1
p-Vimentin_GCSC2
Vimentin_GCSC1
Vimentin_GCSC2

Figure S17D

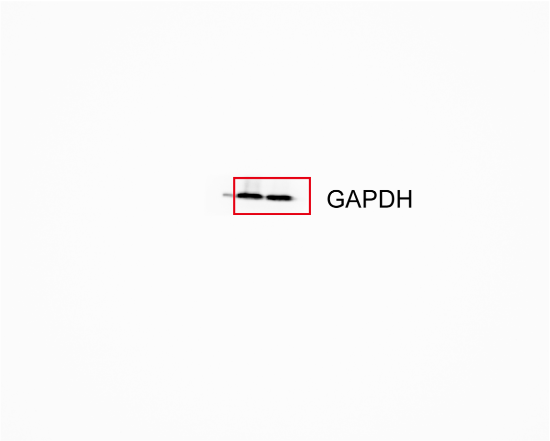

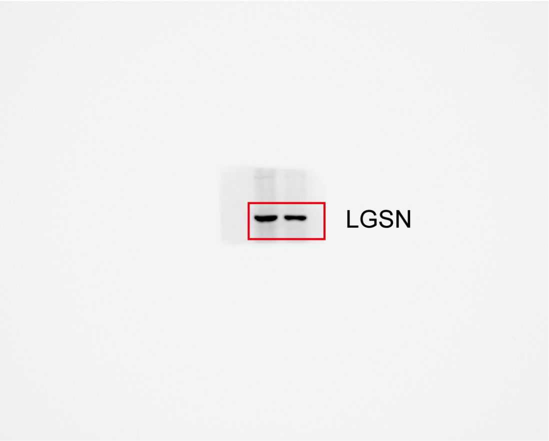

Supplement: Supplementary file 10 — Original Data File [file 41419_2023_6081_MOESM10_ESM.pdf]
